# Isolation of a NHC-stabilized heavier nitrile and its conversion into an isonitrile analogue

**DOI:** 10.1038/s41557-024-01618-6

**Published:** 2024-09-10

**Authors:** Martin E. Doleschal, Arseni Kostenko, Jin Yu Liu, Shigeyoshi Inoue

**Affiliations:** TUM School of Natural Sciences, Department of Chemistry, Catalysis Research Center and Wacker Institute of Silicon Chemistry, Technische Universität Müchen, Garching, Germany

**Keywords:** Chemical bonding, Chemical bonding

## Abstract

Nitriles (R–C≡N) have been investigated since the late eighteenth century and are ubiquitous encounters in organic and inorganic syntheses. In contrast, heavier nitriles, which contain the heavier analogues of carbon and nitrogen, are sparsely investigated species. Here we report the synthesis and isolation of a phosphino-silylene featuring an N-heterocyclic carbene-phosphinidene and a highly sterically demanding silyl group as substituents. Due to its unique structural motif, it can be regarded as a Lewis base-stabilized heavier nitrile. The Si–P bond displays multiple bond character and a bent R–Si–P geometry, the latter indicating fundamental differences between heavier and classical nitriles. In solution, a quantitative unusual rearrangement to a phosphasilenylidene occurs. This rearrangement is consistent with theoretical predictions of rearrangements from heavier nitriles to heavier isonitriles. Our preliminary reactivity studies revealed that both isomers exhibit highly nucleophilic silicon centres capable of oxidative addition and coordination to iron tetracarbonyl.

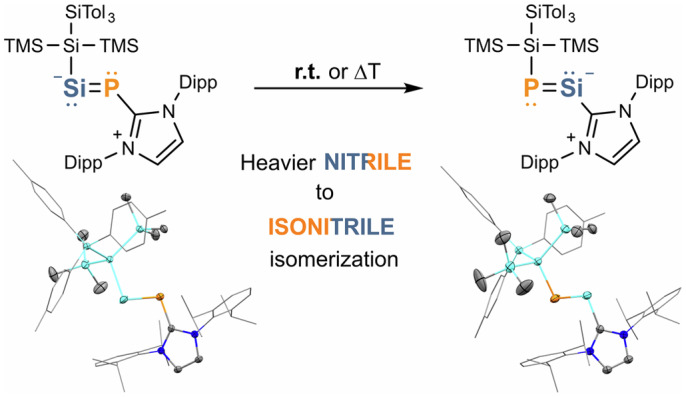

## Abstract (web summary)

Multiple bonds between heavier main-group elements may exhibit different properties and reactivity compared with their second-row counterparts. Now it has been shown that a NHC-stabilized phosphasilyne, a heavier analogue of nitrile, undergoes thermal isomerization to yield a phosphasilenylidene; this represents a rare example of heavier nitrile–isonitrile isomerization.

## Main

The chemical behaviour of main-group elements can be typically substantiated by the count of valence electrons—elements with similar valence electron configurations are expected to exhibit analogous chemical and physical attributes. A key focus in chemistry is to gain an understanding of the similarities and differences of the elements, particularly those in the same periodic group. Compounds bearing low-coordinate carbon are crucial in organic chemistry, constituting a subject of scientific examination for nearly two centuries. Despite their valence isoelectronicity, the heavier homologues of carbon exhibit fundamentally different multiple bonding behaviour due to their relative reluctance towards hybridization^1,2^. In 1981, the hitherto established ‘double bond rule’, which questioned the existence of heavier main-group multiple bonds, was ultimately disproven, after researchers reported the isolation of disilene, diphosphene and silene^3–5^. As of this date, substantial advances have been achieved in the field, with many homo- and heteronuclear diatomic main-group multiple bond compounds being isolable today^6^. Nevertheless, despite the successful isolations of disilynes (Si≡Si)^7^, the synthesis of both phosphasilynes (Si≡P) and diphosphorus (P≡P) remains challenging and has only been observed as intermediates^8,9^. Certain low-valent or low-oxidation-state main-group compounds are found to mimic transition metals in small molecule activation and catalytic activity, presenting conceivable alternatives to costly and environmentally harmful metals^10–12^. Hitherto inaccessible phosphasilyne species have great potential in bond activation, catalysis and in syntheses of Si–P-containing oligomers or polymers^13^.

**Fig. 1:**
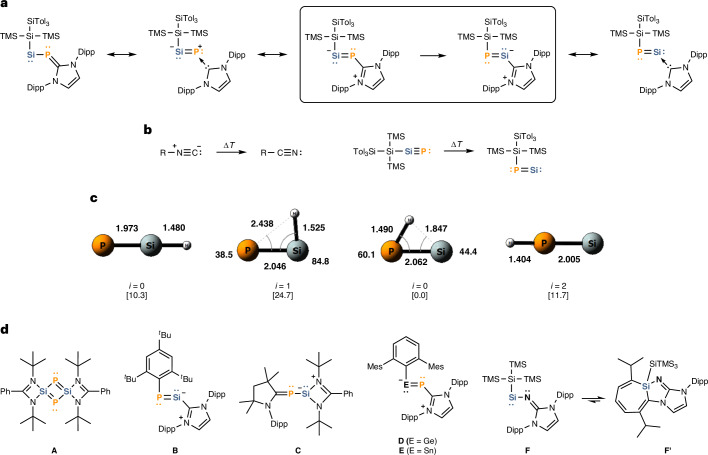
Mesomeric structures of the title compounds, general trends of the isonitrile–nitrile isomerization, theoretical analysis of the HSiP-to-HPSi isomerization, and selected literature compounds related to the elusive heavy nitriles/isonitriles. **a**, Heavier nitrile–isonitrile isomerization shown in this work with selected resonance structures for both structures. **b**, Thermally induced isonitrile–nitrile isomerization known in organic chemistry (left) and the computationally derived isonitrile–nitrile isomerization for the heavier analogues (right). As an example of the latter, the donor-free versions of the title compounds were chosen. Their geometries and bond properties are in good agreement with the parent system (see **c**). For more details and DFT calculations on the donor-free species, see Supplementary Section 7. **c**, Bond lengths and angles of the isomerization of the parent phosphasilyne. The relative energies are given in square brackets. Geometries taken from ref. ^31^. **d**, Selected literature compounds: formal phosphasilyne dimer (**A**); only example of phosphasilenylidene (**B**); carbene-phosphinidene substituted silylene (**C**); NHC-stabilized germylene- (**D**) and stannylene-phosphinidene (**E**); lighter nitrogen congener (**F**) of the title compound and its intramolecular insertion product (**F′**).

Nitriles (R–C≡N) have a vast application in organic syntheses. Observations of isonitrile–nitrile isomerization (Fig. 1b) were made as early as 1873^14^. The kinetics of this rearrangement have been investigated, with computational analyses of isomerization energies documented in recent literature^15,16^. Over the years, researchers have also explored its synthetic potential^17,18^. While isonitrile–nitrile isomerizations typically require elevated temperatures, non-unimolecular rearrangements have been observed at or below ambient temperatures^19,20^. On the other hand, nitrile to isonitrile isomerization remains a scarce finding. The R–CN ⇌ R–NC equilibrium could be observed in compounds featuring more ionic R–CN/R–NC interactions, such as boron and magnesium complexes, and also in Me_3_SiCN (refs. ^21–25^). Heavier nitrile analogues, on the other hand, still pose a difficult synthetic challenge due to their potential di-/oligomerization and low thermodynamic stability^8,26,27^. Regarding this matter, density functional theory (DFT) studies suggest the implementation of electronegative and sterically bulky ligand systems^28,29^. The parent heavier isocyanide HPSi has been generated and investigated in the gas phase. Unlike its lighter congener HNC, which is linear, HPSi adopts a notably bent geometry with H–P interaction^30^.

Computational studies show that the bent heavier isocyanide forms HPE (E = Si, Ge, Sn or Pb) are the global minima on the potential energy surface of those rearrangements (Fig. 1c)^31^. The isomerization of the bent HPSi to the energetically less favoured linear PSiH (at Δ*E* = 10.3 kcal mol^−1^) proceeds via the bent transition state at 24.7 kcal mol^−1^. The linear HPSi geometry corresponds to the second-order saddle point, which is the transition state between the two identical bent HPSi geometries^31^. Whether it is possible to stabilize both stable isomers was investigated in a related study on tin–phosphorus multiple bonds, suggesting that sufficiently bulky substituents potentially favour the PSnR isomer^32^.

Several heavier nitrile intermediates have been proposed by virtue of the synthesis of their formal head-to-tail dimers. As early as 2011, the isolation of **A** (Fig. 1d) was reported independently by two research groups^8,26^. The Si–P bonds within **A** exhibit strong polarization, with bond lengths (2.1701 Å, 2.1717 Å) between those of single and double bonds. In 2016, dimers of phosphagermyne (P≡Ge) were reported, followed by the isolation of an arsagermyne (As≡Ge) dimer 1 year later^33–35^. Phosphasilenylidene **B** featuring a NHC ligand attached to its silicon centre was reported in 2015. **B** can be considered a Lewis base-stabilized heavier isocyanide, exhibiting a silicon–phosphorus double bond (r(Si–P) = 2.119 Å) bearing a two-coordinate silicon atom^36^. These findings, again, underline the impressive capabilities of N-heterocyclic carbenes to stabilize low-valent main-group compounds^37^. Usage of cyclic alkyl amino carbene (cAAC) and an amidinate ligand allowed for the isolation of silylene-phosphinidene **C** in 2017^38^. **C** exhibits a shorter Si–P bond length (2.2572 Å) compared with previous phosphine-functionalized silylenes, which clearly suggests single-bond character^39^. Similar bonding situations were observed after the reduction of bis-cAAC-phosphinidene silicon dichloride^40^. Soon after, the successful synthesis of phosphine- and NHC-stabilized germylene-arsinidienes was achieved, with a phosphorus transfer towards a chlorogermylene to a formal germylene-phophinidene shortly following^35,41^. Our group reported germylene- and stannylene-phosphinidenes **D**/**E**, with two-coordinate tetrylene centres^13^. **D**/**E** exhibit short tetrel-phosphorus bond lengths (2.2364 Å and 2.4562 Å) and undergo thermally reversible [2 + 2] cycloaddition with diphenylketene. Moreover, we demonstrated the catalytic application of **D**/**E** in hydroboration reactions under mild conditions. Notably, germylene-phosphinidene and germylene-arsenidene were isolated using a highly sterically demanding aryl ligand^42,43^. In a recent report, the germanium analogue of heavier isonitriles has been presented^44^. Herein, we report the preparation of a NHC-stabilized silylene-phosphinidene and its thermal isomerization to phosphasilenylidene.

## Results and discussion

### Synthesis and investigation of phosphino-silylene 2

**Fig. 2:**
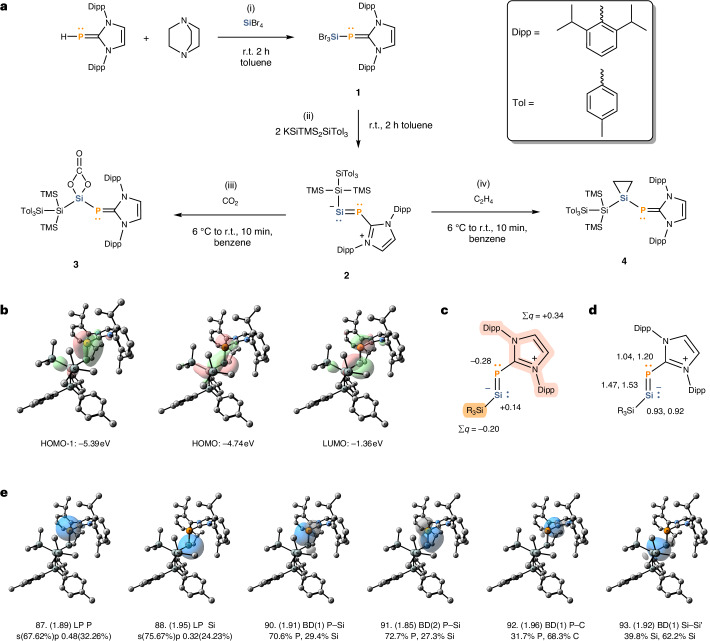
Preparation and theoretical investigation of silylene-phosphinidene 2 as well as some early reactivity studies. **a**, Preparation of **1** via dehydrobromination with parent N-heterocyclic carbene-phosphinidene (NHCP), silicon tetrabromide and 1,4-diazabicyclo[2.2.2]octane (DABCO) (i). Simultaneous metathesis and reduction of **1** with two equivalents of potassium silanide furnished **2** (ii). Exposure of **2** to an excess of carbon dioxide resulted in the formation of silicon carbonate complex **3** (iii). In similar fashion, silirane **4** was obtained in the reaction of **2** with ethylene (iv). **b**, Frontier orbitals of **2**. **c**, Natural population analysis (NPA) charge distribution in **2**. Calculations on bigger fragments in the molecule are highlighted. **d**, WBIs and MBOs for central bonds in **2**. **e**, Selected NBOs of **2** (for a side-by-side comparison of frontier orbitals and NBOs of **2** and **5**, see Supplementary Section 6). r.t., room temperature (18–25 °C); BD, 2-centre bond; LP, 1-centre valence lone pair.

NHC-stabilized silylene-phosphinidene **2** was prepared using a procedure akin to the synthesis of its lighter congener (IDipp)NSi(SiTMS_3_) (IDipp = 1,3-bis(2,6-diisopropylphenyl)-imidazolin-2-ylidene, TMS = trimethylsilyl, **F**), whereby an intramolecular insertion of its silylene centre into the aromatic NHC wingtip forming a silacycloheptatriene (silepin, **F′**) was observed (Fig. 1d)^45^. This, in our case, undesired reactivity was prevented by employing a sterically bulkier silyl ligand. Thus, upon reaction of IDippPSiBr_3_ (**1**) with two equivalents of KSiTMS_2_SiTol_3_ in toluene at ambient temperature, NHC-stabilized silylene-phosphinidene **2** could be isolated in 73% yield (Fig. 2a). Dark green-brown crystals of **2** suitable for single crystal X-ray diffraction (SC-XRD) measurement were obtained from an evaporating diethyl ether solution. Compound **2** has been fully characterized by SC-XRD, nuclear magnetic resonance (NMR), ultraviolet–visible (UV–vis) and mass spectroscopy. It exhibits high solubility in toluene, benzene, diethyl ether and tetrahydrofuran (THF) but barely dissolves in saturated hydrocarbons and acetonitrile.

**Fig. 3:**
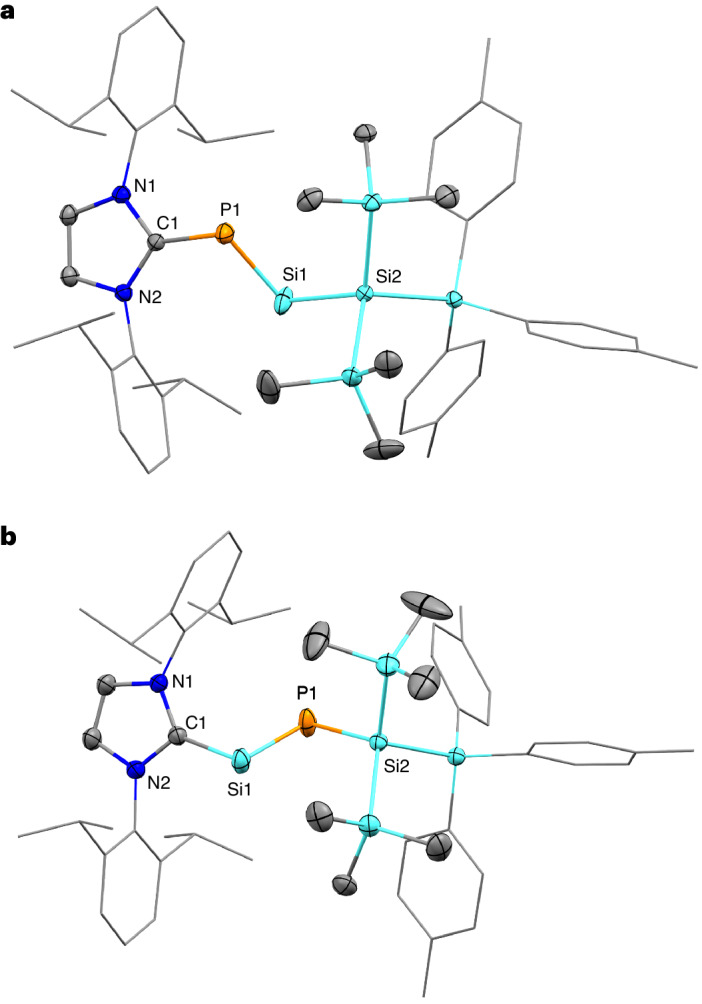
Solid-state plot of the molecular structure of 2 and 5. Thermal ellipsoids are set at 50% probability. Hydrogen atoms are omitted for clarity. Dipp- and tolyl-substituents are depicted as wireframes for simplicity. Selected bond lengths (Å) and angles (°). **a**, Phosphino-silylene **2**: C1–P1: 1.844(2), P1–Si1: 2.1311(7), Si1–Si2: 2.3808(8), C1–P1–Si1: 101.43(6), P1–Si1–Si2: 97.99(3), N1–C1–P1: 124.0(1), N2–C1–P1: 131.2(1). **b**, Phosphasilenylidene **5**: C1–Si1: 1.975(2), Si1–P1: 2.1094(7), P1–Si2: 2.2666(8), C1–Si1–P1: 99.26(5), Si1–P1–Si2: 99.14(3), N1–C1–Si1: 130.3(1), N2–C1–Si1: 124.3(1).

The central silicon and phosphorus atoms in **2** display notably low-field shifted ^29^Si NMR (455.0 ppm, ^1^*J*_Si–P_ = 187.5 Hz) and ^31^P NMR (269.4 ppm) chemical shifts in C_6_D_6_. Even in comparison with other acyclic silylenes, the ^29^Si NMR shift of the low-valent Si atom in **2** appears relatively low-field, which may be attributed to the pronounced P–Si bond polarization^46,47^. Related silylene-phosphinidene **C** exhibits ^29^Si NMR (56.0 ppm) and ^31^P NMR (68.8 ppm) signals in higher-shifted areas, probably due to electron density provided by the amidinate ligand. Compared with **C** (2.257 Å), the Si–P bond length in **2** (2.1311(7) Å) is notably shorter, falling within the range of elongated double bonds (Fig. 3)^48–50^. Additionally, the value is below Si–P bond lengths in heterocyclic 4- (2.1701 Å, 2.1737 Å) and six-membered rings (2.1512 Å) as well as NHC-stabilized hydro-phosphasilene (IMe_4_)(2,6-Tip_2_C_6_H_3_)(H)Si=PTip (2.1585 Å)^8,26,51,52^. The carbene–phosphorus bond length (1.844(2) Å) in **2** is noticeably elongated compared with cAAC-phosphinidene **C** (1.732 Å), parent N-heterocyclic carbene phosphinidene (N(2,6-^*i*^Pr_2_C_6_H_3_)CH)_2_CPH (1.752 Å) or NHC-stabilized diphosphorus ((N(2,6-Pr^*i*^_2_C_6_H_3_)CH)_2_CP)_2_ (1.7504 Å)^53,54^. This indicates weakened bonding interaction between the NHC and the phosphinidene centre. In contrast to the linear geometry of nitriles, the X-ray structure of **2** reveals a *trans*-bent structure with the Si2–Si1–P1 (97.99(3)°) and Si1–P1–C1 (101.43(6)°) angles indicating lone pairs at both silicon and phosphorus. To the best of our knowledge, **2** represents a rare NHC-stabilized phosphasilyne featuring an acyclic silylene with adjacent N-heterocyclic carbene phosphinidene.

To further elucidate the bonding situation and the electronic structure of **2**, quantum chemical calculations were carried out. All compounds have been optimized at the r^2^SCAN-3c level of theory; the properties and the energies of the optimized geometries were calculated at the PBE0/def2-TZVP and ωB97M-V/def2-QZVPP level of theory, respectively. For additional details and references, see Supplementary Section 5. The frontier orbitals of **2** are shown in Fig. 2b. Natural bond orbital (NBO) analysis of the canonical molecular orbitals (MOs) shows that the highest occupied molecular orbital (HOMO) has 40% bonding and 57% non-bonding character, with the largest contribution from the Si lone pair (45%) and additional smaller contributions from σ(Si–Si), phosphorus lone pair, σ(Si–P) and π(Si–P). The HOMO-1 consists mainly of the Si–P π-bond (76%), while the LUMO corresponds to the π*(Si–P) anti-bonding orbital (89%). NBO analysis (Fig. 2e) shows σ-type lone pairs on the central phosphorus (NBO 87) and silicon (NBO 88) atoms. The phosphorus lone pair exhibits a relatively low occupancy (1.89 electrons (el.)) due to the donor–acceptor interactions with the coordinating NHC moiety, mainly with the vicinal anti-bonding C–N orbitals. The two Si–P bonding interactions (NBO 90, 91) are substantially polarized towards the phosphorus (70.6% and 72.7%). Additional interactions of the P–Si fragment are the polarized P–C bond between the phosphorus and the NHC, and a slightly polarized Si–Si bond between the silicon atom and the SiR_3_ substituent. The explicit multiple bond character of the Si–P interaction is reflected in the high Wiberg bond index (WBI 1.47) and Mayer bond order (MBO 1.53) (Fig. 2c,d).

Exposure of **2** to carbon dioxide furnished silicon carbonate complex **3** (Fig. 2a(iii)) in 69% isolated yield. **3** was characterized via XRD, NMR and mass spectroscopy. ^29^Si NMR of its central silicon atom (41.0 ppm) and ^31^P NMR (147.4 ppm) resonances of compound **3** are drastically upfield shifted compared with **2**. The mechanism of such reactivity has been investigated for cyclic silylenes^55^, whereby an analogous reactivity has also been reported for acyclic N-heterocyclic imine silylenes (IDipp)NSi(SiTMS_3_) and (^Me^IDipp)NSi(Si^*t*^Bu_3_)^45,56^. Compared with its silylene precursor, **3** exhibits a shortened C–P bond length (1.796(2) Å), a marginally longer Si–P bond length (2.1448(8) Å) and wider Si2–Si1–P1 (105.05(3)°) and Si1–P1–C1 (115.05(7)°) angles.

In the presence of ethylene, the rapid conversion of **2** to silacyclopropane **4** was observed (Fig. 2a(iv)). **4** was fully characterized by XRD, NMR and mass spectroscopy and isolated in 42% yield. The ^29^Si NMR resonance (−107.1 ppm) of its central silicon atom is high field shifted compared with typical siliranes^57^. However, it is in good agreement with lighter nitrogen congeners^45,56^. ^31^P NMR resonance (166.9 ppm) can be observed at a slightly higher shift. The C–P bond (1.795(2) Å) is contracted, and the Si–P bond (2.2048(6) Å) is substantially elongated, unequivocally representing a single bond. Si2–Si1–P1 (103.77(2)°) and Si1–P1–C1: (115.62(6)°) bond angles in **4** are consistent with those in **3**.

**Fig. 4:**
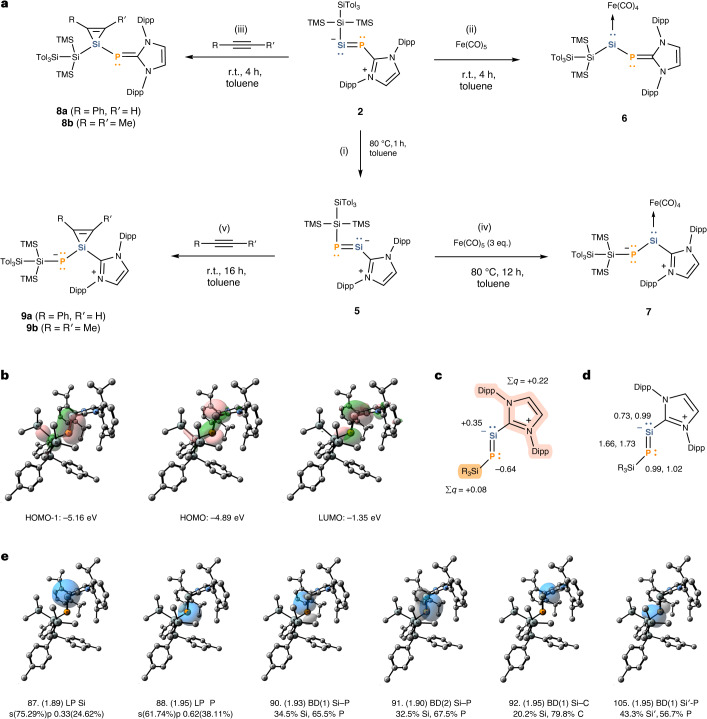
Preparation and theoretical investigation of phosphasilenylidene 5 as well as reactivity comparison with 2. **a**, Thermal isomerization of **2** to **5** (i). Formation of silylene iron complex **6** from the reaction of **2** with FeCO_5_ (ii). Exposure of **2** to phenylacetylene or dimethyl acetylene furnished the respective silirenes **8a** and **8b** (iii). The reaction of **5** with a slight excess of FeCO_5_ affords phosphasilenylidene iron complex **7** (iv). **5** shows the oxidative addition of phenylacetylene and dimethyl acetylene to form NHC-stabilized phosphinilydene silacyclopropenes **9a** and **9b**. **b**, Frontier orbitals of **5**. **c**, Natural population analysis (NPA) charge distribution in **5**. Calculations on bigger fragments in the molecule are highlighted. **d**, WBIs and MBOs for central bonds in **5**. **e**, Selected NBOs of **5**. r.t., room temperature (18–25 °C); BD, 2-centre bond; LP, 1-centre valence lone pair.

The above-described reactivity of **2** towards CO_2_ and ethylene (Fig. 2a), as well as with acetylene (Fig. 4a), is analogous to the reactivity of silylenes^46,56^. To evaluate the donor–acceptor abilities of **2** and compare them with other silylenes, the proton affinity (PA) and relative P–H rotational barrier (RRB) of **2** were calculated using a previously benchmarked methodology^58^. **2** exhibits a high PA of 1,170 kJ mol^−1^, very similar to the ambiphilic (imino)(silyl)silylene (1,176 kJ mol^−1^)^59^. However, the RRB is 0.347, considerably lower than that of the (imino)(silyl)silylene (0.562). Under these considerations, **2** is expected to be a much weaker ambiphile compared with the (imino)(silyl)silylene owing to its diminished π-acceptor properties, despite the predicted strong σ-donor properties.

### Isomerization to phosphasilenylidene 5

In solid state, **2** appears as a green powder and is indefinitely stable under inert conditions. Leaving **2** in benzene, toluene or THF solution for a prolonged period of time results in a colour change from dark green to transparent red. NMR spectroscopy shows that the conversion of **2** to a new species (**5**) proceeds at room temperature, (66% after 14 days) and can be accelerated by heating the reaction mixture to 80 °C (100% after 1 h). While there are only minor changes in the ^1^H and ^31^P NMR (270.5 ppm), the ^29^Si NMR chemical shift of the central silicon in **5** (337.6 ppm, ^1^*J*_Si–P_ = 182.4 Hz) differs greatly from the one observed in **2** (455.0 ppm, ^1^*J*_Si–P_ = 187.5 Hz). Furthermore, the carbene *J*_C–P_ coupling of 17 Hz drastically varies from **2** (*J*_C–P_ = 132.6 Hz), falling within the range of typical ^2^*J*_C–P_ values^60^. These spectroscopic results suggested an isomerization of **2** to the corresponding phosphasilenylidene **5** (Fig. 4a(i)), in a process similar to the previously theoretically studied rearrangement of heavy nitriles^31^. The molecular structure of the proposed phosphasilenylidene **5** is confirmed by SC-XRD analysis. Skeletal rearrangements in heavy-main-group-element-based species have been observed; however, examples of such rearrangements are rare^61–64^. In our case, the P–Si atom exchange occurs between two adjacent positions in a planar geometry. In relation to previous landmark developments, compound **5** is a group 15 analogue of heavier vinylidenes^65,66^. These have also shown to undergo rearrangement/isomerization reactions involving heavy main-group elements^62,67,68^.

The X-ray structure of **5** (Fig. 3) features a Si–P bond (2.1094(7) Å) considerably shorter than the Si–P distance in **2** (2.1311(7) Å) falling in the range of typical Si=P bonds^69^. This indicates a higher silicon–phosphorus multiple bond character. Si2–P1–Si1 (99.14(3)°) and P1–Si1–C1 (99.26(5)°) bond angles are comparable to the respective Si2–Si1–P1 and Si1–P1–C1 angles in **2**. Compared with **B** (402.4 ppm), the ^31^P NMR shift of **5** (270.5 ppm) is high field shifted, presumably due to the more electron-withdrawing aryl substituent in **B**. This, in turn, leads to the ^29^Si NMR chemical shift of the central Si atom in **5** (337.6 ppm) being low field shifted relative to **B** (267.3 ppm). The Si–P (2.1094(7) Å) and Si–C (1.975(2) Å) bond lengths in **5** are very similar to those in **B** (2.119 Å, 1.960 Å), whereas **B** exhibits slightly narrower C_Ligand_–P1–Si1 (95.4°) and P1–Si1–C_NHC_ (96.9°) bond angles^36^.

The frontier orbitals of **5** are shown in Fig. 4b. According to the NBO analysis of canonical MOs, the HOMO of **5** is mostly of non-bonding character (61%), which is attributed to the lone pairs of Si (32%) and P (27%). The 35% bonding character of the HOMO originates predominantly from σ(Si–P) and π(Si–Si). The HOMO-1 is 96% bonding orbital and consists primarily of the Si–P π-bond (76%), while the lowest unoccupied molecular orbital (LUMO) is 85% anti-bonding and composed mainly of π*(Si–P) (57%), with a smaller contribution from the vicinal π*(C–N) and the σ*(Si–P). NBO analysis (Fig. 4e) shows σ-type lone pairs on the central silicon (NBO 87) and the phosphorus atoms (NBO 88). The somewhat low occupancy (1.89 el.) of the Si lone pair NBO, similar to that of the lone pair at the phosphorus centre of **2**, is due to the delocalization to the vicinal anti-bonding C–N orbitals. The two bonding interactions between Si and P (Fig. 4e, NBO 90 and 91) are less polarized (65.5% and 67.5%) towards phosphorus compared to **2**. In contrast, the bond between the silicon centre and the NHC carbon is substantially polarized (79.8%) towards carbon. There is little polarization between the central phosphorus and the SiR_3_ substituent. In **5**, a considerable multiple bond character of the Si–P interaction is suggested by the high WBI (1.66) and MBO (1.73) (Fig. 4d), which both exceed those calculated for **2**. To assess the donor–acceptor abilities of the silicon centre in **5**, PA and RRB were calculated. **5** exhibits PA of 1143 kJ mol^−1^ slightly lower than that of **2** (1,170 kJ mol^−1^), indicating mildly diminished σ-donor abilities. On the other hand, the RRB value of 0.282 is noticeably lower than that of **2** (0.347), indicating that the Si centre in **5** is a poorer π-acceptor.

**Fig. 5:**
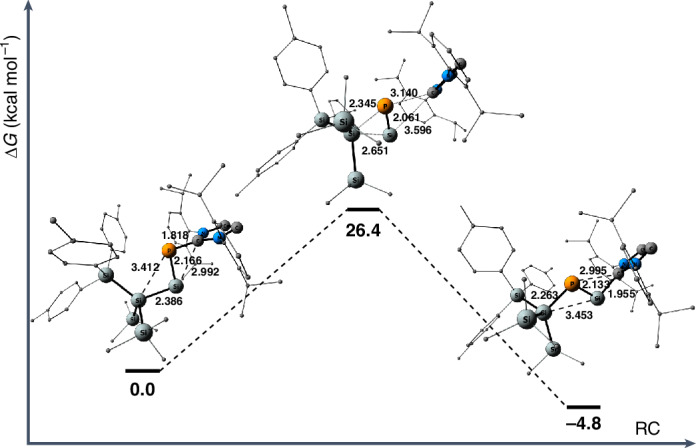
Calculated reaction coordinate for the proposed mechanism of the 2 → 5 isomerization. Phosphino-silylene **2** (0.0 kcal mol^−1^) isomerizes to phosphasilenylidene **5** (−4.8 kcal mol^−1^) with an energy barrier of 26.4 kcal mol^−1^. A bigger version of this figure is provided in Supplementary Section 6. RC, reaction coordinate.

DFT calculations suggest the isomerization of **2** to **5** can proceed in one step, with Δ*G*^‡^ at 26.4 kcal mol^−1^ (Fig. 5), which is in good agreement with the experimentally estimated 25.8 kcal mol^−1^. The driving force of the reaction is the relative stability of isomer **5** compared with **2**, as the reaction is calculated to be exergonic by 4.8 kcal mol^−1^. For a better understanding of how our compounds reflect the properties of heavier (iso)nitriles, we calculated the NHC-free versions of compounds **2** and **5** (Fig. 1b). An in-depth investigation into their geometries, electronic structure and isomerization mechanism can be found in Supplementary Section 7.

### Further reactivity studies of 2 and 5

While **2** reacts with CO_2_ and H_2_CCH_2_ to form the corresponding silicon carbonate and silacyclopropane (Fig. 2a(iii),(iv)), exposure of **5** to these gases led to complex mixtures of product. However, both **2** and **5** react selectively with iron carbonyl, which is a commonly used Lewis acid for stabilization and isolation of low-valent silicon compounds^69–71^. **2** quickly forms iron complex **6** in the presence of Fe(CO)_5_. Its ^31^P NMR (153.2 ppm) and ^29^Si NMR (349.5 ppm at −30 °C) resonances are upfield shifted, with the latter being in good agreement with literature-reported silylene iron complexes. Infrared spectroscopy of **6** revealed CO vibration bands at 1,885, 1,933 and 2,011 cm^−1^, which are within typical ranges known for similar complexes^72^. The reaction of **5** with Fe(CO)_5_ is slower and requires heating with excess iron-carbonyl to form phosphasilenylidene iron complex **7**. Its ^29^Si NMR signal (240.9 ppm, −30 °C) could only be observed at lower temperatures. **7** exhibits a slightly less upfield shifted ^31^P NMR (189.7 ppm) resonance than **6**. A small Si–P bond length (2.0966(7) Å) shorter than Si–P bonds in **2** and **5** suggests the double bond character is maintained. The Si2–P1–Si1 angle (112.08(3)°) at the phosphorus atom is wider, and the sum of angles around the low-coordinate silicon centre (359.7°) shows trigonal planar geometry. Compound **7** exhibits a Si–Fe bond length (2.2693(6) Å) within the typical range (2.15–2.36 Å) of silylene iron complexes^69–71^. CO vibration bands of **7** can be observed at 1,890, 1,936 and 2,013 cm^−1^, indicating similar donor properties of **2** and **5** considering the negligible difference to **6**.

Additional comparison of reactivity between **2** and **5** could be drawn from their reactions with acetylene derivatives (Fig. 4a(iii),(v)). **2** reacts with phenylacetylene and 2-butyne to form the respective silacyclopropenes (silirenes) **8a** and **8b**, which are common reaction products of silylenes with alkynes^73^. **8a** and **8b** share similar spectroscopic properties. As expected, ^29^Si NMR signals (−103.6 ppm and −101.6 ppm) and ^31^P NMR signals (−154.8 ppm and −155.2 ppm) are high field shifted. It should be noted that silirane **4** and silirenes **8a** and **8b** are not indefinitely stable in solution and slowly decompose to unidentifiable species after a few days.

The reaction of phosphasilenylidene **5** with the same alkynes affords the NHC-stabilized phosphinilydene silacyclopropenes **9a** and **9b**. Their respective ^29^Si NMR resonances (−88.8 ppm and −89.2 ppm) are notably high field shifted compared with **5** (337.6 ppm), but in lower fields than siliranes **8a** and **8b** or the diphenyl substituted silirene derived from (^Me^IDipp)NSi(Si^*t*^Bu_3_)^56^. The ^31^P NMR resonances (−372.4 ppm and −376.6 ppm) show a pronounced upfield shift, indicating high polarization at the phosphorus centre, similar to the zwitterionic phosphasilene LSi=PH (L=CH[(C=CH_2_)CMe(*N*-2,6-^*i*^Pr_2_C_6_H_3_)_2_]) (−293.9 ppm), and its transition metal complexes^74^. Compared with **5**, compound **9a** exhibits a slightly elongated Si–P bond length (2.129(1) Å) but a shortened C–Si bond length (1.938(2) Å). In contrast to **4**, **8a** and **8b**, the NHC-stabilized phosphinilydene silacyclopropenes **9a** and **9b** do not show decomposition in benzene or THF solution at room temperature. These reactivity studies demonstrate that the silicon centres in both **2** and **5** exhibit typical silylene reactivity.

## Conclusions

In this study, we successfully synthesized and fully characterized the heavier silicon/phosphorus analogue of nitrile (**2**). XRD analysis and DFT calculations indicate that **2** features substantial Si–P multiple bond character. In view of the linear structure of nitriles, the bent geometry of **2** stands out and suggests different electronic properties, which is also reflected by the experimentally determined acyclic silylene character. Thermal isomerization of **2** to heavier isonitrile **5** was observed in solution, aligning with computational predictions, and providing experimental validation for **2** as a silicon analogue of heavier nitriles. Such nitrile–isonitrile isomerization demonstrates the intrinsic difference between organic compounds and their heavy analogues. The highly reactive silicon centre in **2** allows for oxidative addition to various unsaturated small molecules. A potential application of **2** and **5** in coordination chemistry was demonstrated with the isolation of iron complexes **6** and **7**. Additionally, with the isolation of NHC-stabilized phosphinilydene silacyclopropenes **9a** and **9b**, we unveiled some reactivity of heavier isonitrile **5** towards acetylene derivatives.

## Methods

### Synthetic methods

All experiments were performed under a dry argon (≥99.996%) atmosphere using standard Schlenk techniques or in a glovebox (MBraun GmbH). Glassware was heat-dried under vacuum before use. Standard chemicals were purchased from commercial distributors ABCR GmbH, Carl Roth, Merck KGaA, Sigma-Aldrich and TCI Co. Ltd. and used as received. Non-deuterated solvents were distilled over elemental sodium/benzophenone and stored over molecular sieve. All deuterated solvents were stored over molecular sieve. IDipp∙HCl^75^ and IDipp-PH^54^ were synthesized exactly as described in the literature. KSiTMS_2_SiTol_3_ (ref. ^76^) was synthesized analogue to literature procedures with slight modifications (ClSiPh_3_ was replaced with ClSiTol_3_; ref. ^77^). See Supplementary Information for further details.

### Spectroscopic and analytical methods

NMR samples were prepared under an argon atmosphere and measured in J. Young polytetrafluoroethylene (Teflon) valve NMR tubes. NMR spectra were recorded on Bruker AV-400 or AV-500C spectrometers at ambient temperature (300 K). ^1^H, ^13^C and ^29^Si chemical shifts *δ* are reported in parts per million (ppm) relative to tetramethylsilane. *δ*(^1^H) and *δ*(^13^C) were referenced internally to the relevant residual solvent resonances. *δ*(^29^Si) was referenced to the signal of tetramethylsilane (*δ* = 0 ppm) as external standard. For reported signals the following abbreviations are used: s, singlet; d, doublet; t, triplet; hept, heptet; m, multiplet/signal overlap; br., broad signal.

Liquid injection field desorption ionization mass spectrometry was measured directly from an inert atmosphere glovebox with a Thermo Fisher Scientific Exactive Plus Orbitrap equipped with an ion source from Linden CMS^78^.

Fourier-transform infrared spectra were recorded on a Vertex 70 from Bruker with a platinum attenuated total reflectance (ATR) unit. A solution of the sample in pentane was drop-casted onto the ATR crystal and dried under a stream of nitrogen.

UV–vis spectra were recorded on an Agilent Cary 60 UV–vis spectrometer in benzene at room temperature.

Melting points were determined in sealed glass capillaries under inert gas atmosphere using a Büchi B-540 melting point apparatus.

The **2** → **5** rearrangement was monitored via ^31^P NMR spectroscopy in deuterated toluene at ambient and elevated temperatures. Δ*G*^‡^ was calculated using the Eyring equation assuming a first-order reaction. *t*_1/2_ was determined to be 44,210 min/30 days (18 °C), 164 min (60 °C) and 42 min (70 °C), respectively. These results give Δ*G*^‡^ values of 107.8 kJ mol^−1^, 108.4 kJ mol^−1^ and 107.9 kJ mol^−1^ and, therefore, an average of 108.0 kJ mol^−1^ or 25.8 kcal mol^−1^. Stacked spectra and conversion tables can be found in ‘NMR spectra’ section of Supplementary Information.

### Crystallographic methods

Data were collected on a single-crystal X-ray diffractometer equipped with a Charge-Integrating Pixel Array Detector (Brucker Photon-II), a Microfocus X-Ray Source microsource with a CuKα (*λ* = 1.54178) or a Turbo X-Ray Source rotating anode with MoKα radiation (*λ* = 0.71073 Å) and a Helios optic using the APEX4 software package^79^. The crystal was fixed on the top of a Kapton micro sampler with perfluorinated ether and transferred to the diffractometer and frozen under a stream of cold nitrogen. A matrix scan was used to determine the initial lattice parameters. Reflections were corrected for Lorentz and polarization effects, scan speed and background using SAINT^80,81^. Absorption correction, including odd- and even-ordered spherical harmonics, was performed using SADABS^80,81^. Space group assignment was based upon systematic absences, *E* statistics and successful refinement of the structure. The structures were solved using SHELXT with the aid of successive difference Fourier maps and were refined against all data using SHELXL in conjunction with SHELXLE^82–84^. Hydrogen atoms (except on heteroatoms) were calculated in ideal positions as follows: methyl hydrogen atoms were refined as part of rigid rotating groups, with a C–H distance of 0.98 Å and Uiso(H) = 1.5 × Ueq(C). Non-methyl H atoms were placed in calculated positions and refined using a riding model with methylene, aromatic and other C–H distances of 0.99 Å, 0.95 Å and 1.00 Å, respectively, and Uiso(H) = 1.2 × Ueq(C). Non-hydrogen atoms were refined with anisotropic displacement parameters. Full-matrix least-squares refinements were carried out by minimizing Σ*w*(Fo2 Fc2)2 with the SHELXL weighting scheme. Neutral atom scattering factors for all atoms and anomalous dispersion corrections for the non-hydrogen atoms were taken from International Tables for Crystallography^85^. Co-crystallized pentane and phenyl groups of the silirane were disordered and modelled using free variables in conjunction with ISOR, SIMU, RIGU, SADI and SAME restraints as implemented in the Disordered Structure Refinement plugin in SHELXLE^86,87^. The unit cell of structure **9a** contained disordered toluene molecules that could not be modelled reasonably and were treated as a diffuse contribution to the overall scattering without specific atom positions using the SQUEEZE routine in PLATON^88^. Images of the crystal structure were generated with Mercury and PLATON^87,89^. Deposition number 2325629-2325634 contains the supplementary crystallographic data for this paper. These data are provided free of charge by the joint Cambridge Crystallographic Data Centre and Fachinformationszentrum Karlsruhe Access Structures service at www.ccdc.cam.ac.uk/structures.

### Computational methods

Calculations were carried out using ORCA 5.0.4 software^90^.

Geometry optimizations were carried out using the r^2^SCAN-3c composite method, utilizing the regularized and restored SCAN (r^2^SCAN) functional^91,92^, geometrical counterpoise correction gCP^93^, the atom-pairwise dispersion correction based on tight binding partial charges (D4)^94–96^, the def2-mTZVPP basis set and the def2-mTZVPP/J auxiliary basis set^97^.

The optimized geometries were verified as minima or transition states by analytical frequency calculations. The transition states were additionally verified by intrinsic reaction coordinate calculations. Single-point calculations of the optimized geometries were carried out at the r^2^SCAN-3c level using the SMD solvation module^98^ to obtain electrostatic contribution and the cavity term to account for the solvent effects. For more accurate electronic energies, single-point calculations of the r^2^SCAN-3c optimized geometries were carried using the ωB97M-V^99^ functional, the def2-QZVP^100^ basis set and the def2/J^101^ auxiliary basis set. The method is denoted as ωB97M-V(SMD)/def2-QZVP//r^2^SCAN-3c. The NBO analysis was done using the NBO7 software^102^, at the PBE0^103^/def2-TZVP^100^//r^2^SCAN-3c level of theory.

## Online content

Any methods, additional references, Nature Portfolio reporting summaries, source data, extended data, supplementary information, acknowledgements, peer review information; details of author contributions and competing interests; and statements of data and code availability are available at 10.1038/s41557-024-01618-6.

## Supplementary information

Supplementary Information Supplementary Figs. 1–79, Tables 1–4, synthetic procedures for all compounds, crystallographic and computational details.

Supplementary Data 1 Cartesian coordinates for all calculated structures.

Supplementary Data 2 Crystallographic data for compound **2**, CCDC 2325629.

Supplementary Data 3 Crystallographic data for compound **3**, CCDC 2325631.

Supplementary Data 4 Crystallographic data for compound **4**, CCDC 2325630.

Supplementary Data 5 Crystallographic data for compound **5**, CCDC 2325632.

Supplementary Data 6 Crystallographic data for compound **7**, CCDC 2325633.

Supplementary Data 7 Crystallographic data for compound **9a**, CCDC 2325634.

## Data Availability

All data generated or analysed during this study are included in this published Article and its Supplementary Information files. The structures of compounds **2**, **3**, **4**, **5**, **7** and **9a** were determined by single-crystal XRD. Crystallographic data for the structures reported in this Article have been deposited at the Cambridge Crystallographic Data Centre, under deposition numbers CCDC 2325629 (**2**), 2325631 (**3**), 2325630 (**4**), 2325632 (**5**), 2325633 (**7**) and 2325634 (**9a**). Copies of the data can be obtained free of charge via https://www.ccdc.cam.ac.uk/structures/. The electronic energies of all optimized and calculated structures are summarized in Supplementary Information. All non-default parameters for the computational studies are given in Supplementary Information together with the corresponding references of the methods used. The Cartesian coordinates of the structures are provided in a supplementary data file. These files comprise all the necessary data for reproducing the values.
