# Supplementary material for: Isolation of a NHC-stabilized heavier nitrile and its conversion into an isonitrile analogue
Source: Nat Chem. 2024 Sep 10;16(12):2009–16. doi: 10.1038/s41557-024-01618-6 (PMC11611736; doi:10.1038/s41557-024-01618-6)
Supplement: Supplementary file 1 — Supplementary Figs. 1–79, Tables 1–4, synthetic procedures for all compounds, crystallographic and computational details. [file 41557_2024_1618_MOESM1_ESM.pdf]

nature chemistry

Supplementary information https://doi.org/10.1038/s41557-024-01618-6

Isolation of a NHC-stabilized heavier nitrile and its conversion into an isonitrile analogue

In the format provided by the authors and unedited

Table of content

1. Experimental Section **2**
General Procedures 2
Chemicals & Materials 2
NMR Spectroscopy 2
Mass Spectroscopy 2
IR Spectroscopy 3
UV-VIS Spectroscopy 3
Melting Points 3
Determination of $\Delta G^{\ddagger}$ for the Rearrangement 3
XRD 3
2. Synthetic Procedures **5**
Synthesis of IDipp-PSiBr$_3$ (**1**) 5
Synthesis of (IDipp)PSi(SiTMS$_2$SiTol$_3$) (**2**) 6
Synthesis of (IDipp)PSiCO$_3$(SiTMS$_2$SiTol$_3$) (**3**) 8
Synthesis of (IDipp)PSiC$_2$H$_4$(SiTMS$_2$SiTol$_3$) (**4**) 9
Synthesis of (IDipp)SiP(SiTMS$_2$SiTol$_3$) (**5**) 10
Synthesis of (IDipp)PSi[Fe(CO$_4$)](SiTMS$_2$SiTol$_3$) (**6**) 11
Synthesis of (IDipp)Si[Fe(CO$_4$)]P(SiTMS$_2$SiTol$_3$) (**7**) 12
Synthesis of (IDipp)PSi[PhC$_2$H](SiTMS$_2$SiTol$_3$) / (IDipp)PSi[C$_4$H$_6$](SiTMS$_2$SiTol$_3$) (**8a/8b**) .. 13
Synthesis of (IDipp)Si[PhC$_2$H]P(SiTMS$_2$SiTol$_3$) / (IDipp)Si[C$_4$H$_6$]P(SiTMS$_2$SiTol$_3$) (**9a/9b**) .. 15
3. Spectra **17**
NMR 17
LIFDI-MS 43
IR 48
UV-VIS 49
Additional Spectra of Precursors 50
4. SC-XRD Analysis **56**
5. Computational Details **73**
6. Side-by-side comparison of MOs and NBOs in 2/5 .... **75**
7. Calculations of NHC-Free Derivatives of 2 (10) and 5 (11) **80**
8. References **82**

1. Experimental Section

1.1. General Procedures

Chemicals & Materials

All experiments were performed under dry argon (≥99.996%) atmosphere using standard *Schlenk* techniques or in a glovebox (*MBraun GmbH*). Glassware was heat-dried under vacuum prior to use. Standard chemicals were purchased from commercial distributors *ABCR GmbH*, *Carl Roth*®, *Merck KGaA*, *Sigma-Aldrich*® and *TCI Co. Ltd*. and used as received. Non-deuterated solvents were distilled over elemental sodium/benzophenone and stored over molecular sieve. Deuterated solvents were stored over molecular sieve. IDipp · HCl[1], IDipp-PH[2] were synthesized as described in literature. $KSiTMS_2SiTol_3$[3] was synthesised analogue to literature procedures with slight modifications ($ClSiPh_3$ was replaced with $ClSiTol_3$).

NMR Spectroscopy

NMR samples were prepared under an argon atmosphere and measured in *J. Young* PTFE valve NMR tubes. NMR spectra were recorded on *Bruker* AV-400 or AV-500C spectrometers at ambient temperature (300 K). ^{1}H, ^{13}C and ^{29}Si chemical shifts δ are reported in parts per million (ppm) relative to tetramethylsilane. δ(^{1}H) and δ(^{13}C) were referenced internally to the relevant residual solvent resonances. δ(^{29}Si) was referenced to the signal of tetramethylsilane (δ = 0 ppm) as external standard. For reported signals the following abbreviations are used: s = singlet, d = doublet, t = triplet, hept = heptet, m = multiplet/signal overlap, br. = broad signal.

Mass Spectroscopy

Liquid Injection Field Desorption Ionization Mass Spectrometry (LIFDI-MS) was measured directly from an inert atmosphere glovebox with a Thermo Fisher Scientific Exactive Plus Orbitrap equipped with an ion source from Linden CMS.[4]

IR-Spectroscopy

FT-IR spectra were recorded on a Vertex 70 from Bruker with a Platinum ATR unit. A solution of the sample in pentane was drop-casted onto the ATR crystal and dried under a stream of nitrogen.

UV-VIS Spectroscopy

UV-VIS spectra were recorded on an Agilent Cary 60 UV-Vis spectrometer in benzene at room temperature.

Melting points

Melting Points (m.p.) were determined in sealed glass capillaries under inert gas atmosphere using a *Büchi B-540* melting point apparatus.

Determination of $\Delta G^{‡}$ for the Rearrangement

The **2** → **5** rearrangement was monitored *via* ^{31}P NMR spectroscopy in deuterated toluene at ambient and elevated temperatures. $\Delta G^{‡}$ was calculated using the *Eyring* equation assuming a first-order reaction. $t_{1/2}$ was determined to be 44210 min / 30 d (18°C), 164 min (60°C) and 42 min (70°C) respectively. These results give $\Delta G^{‡}$ values of 107.8 $kJmol^{-1}$, 108.4 $kJmol^{-1}$, and 107.9 $kJmol^{-1}$, therefore an average of 108.0 $kJmol^{-1}$ or 25.8 $kcalmol^{-1}$. Stacked spectra and conversion tables can be found in the NMR-Spectra section.

SC-XRD

Data were collected on a single crystal X-ray diffractometer equipped with a CPAD detector (Brucker Photon-II), an IMS microsource with a CuKα (λ = 1.54178) or a TXS rotating anode with MoKα radiation (λ = 0.71073 Å) and a Helios optic using the APEX4

software package.[5] The crystal was fixed on the top of a kapton micro sampler with perfluorinated ether and transferred to the diffractometer and frozen under a stream of cold nitrogen. A matrix scan was used to determine the initial lattice parameters. Reflections were corrected for Lorentz and polarisation effects, scan speed, and background using SAINT.[6] Absorption correction, including odd and even ordered spherical harmonics was performed using SADABS.[6] Space group assignment was based upon systematic absences, E statistics, and successful refinement of the structure. The structures were solved using SHELXT with the aid of successive difference Fourier maps and were refined against all data using SHELXL in conjunction with SHELXLE.[7-9] Hydrogen atoms (except on heteroatoms) were calculated in ideal positions as follows: Methyl hydrogen atoms were refined as part of rigid rotating groups, with a C–H distance of 0.98 Å and Uiso(H) = 1.5·Ueq(C). Non-methyl H atoms were placed in calculated positions and refined using a riding model with methylene, aromatic, and other C–H distances of 0.99 Å, 0.95 Å, and 1.00 Å, respectively, and Uiso(H) = 1.2·Ueq(C). Non-hydrogen atoms were refined with anisotropic displacement parameters. Full-matrix least-squares refinements were carried out by minimizing Σw(Fo2 Fc2)2 with the SHELXL weighting scheme. Neutral atom scattering factors for all atoms and anomalous dispersion corrections for the non-hydrogen atoms were taken from International Tables for Crystallography.[10] Co-crystallized pentane and phenyl groups of the silirane were disordered and modelled using free variables in conjunction with ISOR, SIMU, RIGU, SADI, and SAME restraints as implemented in the DSR plugin in SHELXLE.[11,12] The unit cell of structure **9a** contained disordered toluene molecules which could not be modelled reasonably and were treated as a diffuse contribution to the overall scattering without specific atom positions using the SQUEEZE routine in PLATON.[13] Images of the crystal structure were generated with Mercury and PLATON.[12,14] Deposition Number 2325629-2325634 contains the supplementary crystallographic data for this paper. These data are provided free of charge by the joint Cambridge Crystallographic Data Centre and Fachinformationszentrum Karlsruhe Access Structures service www.ccdc.cam.ac.uk/structures.

2. Synthetic Procedures

Synthesis of IDippPSiBr$_3$ (1):

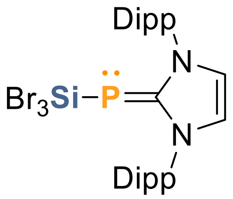

1

IDipp-PH (5.00 g, 11.94 mmol, 1.00 eq.) and 1,4-Diaza-bicyclo[2.2.2]octane (1.37 g, 12.18 mmol, 1.02 eq.) were dissolved in toluene and under vigorous stirring a mixture of $SiBr_4$ (1.51 mL, 12.18 mmol, 1.02 eq.) in toluene (10 mL) was added dropwise. The solution immediately turned yellow/orange, and the formation of a white precipitate was observed. After stirring for two hours the reaction mixture was filtered. By removing the remaining solvent *in vacuo*, IDipp-PSiBr$_3$ (7.89 g, 96%) could be obtained in excellent yield. With minimal remaining DABCO its purity satisfied the requirements for direct use in follow-up reactions.

^{1}H NMR (400 MHz, C_6D_6): δ 7.20 (t, 2H, *p*-C*H*-Ar), 7.09 (d, J = 7.8 Hz, 4H, *m*-C*H*-Ar), 6.33 (s, 2H, N-C*H*), 2.87 (hept, J = 6.9 Hz, 4H, C*H*), 1.46 (d, J = 6.7 Hz, 12H, CH_3), 0.99 (d, J = 6.9 Hz, 12H, CH_3).

^{13}C NMR (126 MHz, C_6D_6): δ 168.52 (d, J = 115.5 Hz, *C*=P), 146.15 (^{Dipp}Ar-*C*H), 133.93 (^{Dipp}Ar-*C*H), 131.34 (^{Dipp}Ar-*C*H), 125.32 (^{Dipp}Ar-*C*H), 123.64 (d, J = 3.2 Hz, N-*C*H), 29.20 (*i*Pr-*C*H), 25.52 (*C*H_3), 23.49 (d, J = 2.3 Hz, *C*H_3).

^{29}Si NMR (99 MHz, C_6D_6): δ -41.01 (d, J = 196.8 Hz).

^{31}P NMR (162 MHz, C_6D_6): δ -77.65.

m.p.: 189.0 – 191 °C (decomposition, gradually turns dark orange and melts, no colour change back to yellow)

Synthesis of (IDipp)PSi(SiTMS$_2$SiTol$_3$) (2):

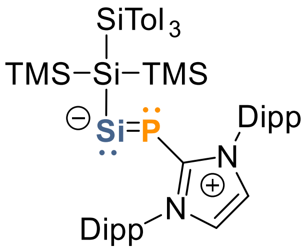

2

IDipp-PSiBr$_3$ (**1**; 1.00 g, 1.45 mmol, 1.00 eq.) was dissolved in toluene, and under vigorous stirring, a solution of KSiTMS$_2$SiTol$_3$ (1.54g, 2.98 mmol, 2.05 eq.) in toluene was added. The reaction mixture turned dark brown and was stirred for two more hours. Subsequently, potassium bromide was removed *via* filtration (facilitated by diluting with little toluene). Solvent from the filtrate was thoroughly removed over several hours until green crystalline areas were observed in the otherwise slimy residue. Most of the by-product BrSiTMS$_2$SiTol$_3$ and all non-solid material could be removed by washing with hexane multiple times. The dark-green residue was dried *in vacuo* to yield 0.98 g (73%) of **2** as a pale-green solid. Crystals of **2** suitable for XRD analysis could be obtained from an evaporating diethyl ether solution at room temperature.

^{1}H NMR (400 MHz, C$_6$D$_6$): δ 7.83 (d, J = 7.6 Hz, 6H, ^{Tol}*o*-C*H*), 7.23 (t, J = 7.8 Hz, 2H, ^{Dipp}*p*-C*H*), 7.11 – 7.02 (m, 10H, ^{Tol}*m-CH* + ^{Dipp}*m*-C*H*), 6.48 (s, 2H, N-C*H*), 2.92 (hept, J = 6.9 Hz, 4H, C*H*), 2.17 (s, 9H, ^{Tol}C*H*$_3$), 1.31 (d, J = 6.8 Hz, 12H, ^{Dipp}C*H*$_3$), 1.06 (d, J = 6.9 Hz, 12H, ^{Dipp}C*H*$_3$), 0.27 (s, 18H, *TMS*).

^{13}C NMR (126 MHz, C$_6$D$_6$): δ 172.95 (d, J = 132.6 Hz, C=P), 145.96 (^{Dipp}Ar*C*), 137.46 (^{Tol}*o*-*C*H), 136.87 (^{Tol}*C*-Me), 136.31 (^{Tol}Si-*C*), 134.08 (^{Dipp}N-*C*), 130.82 (^{Dipp}*p*-*C*H), 128.63 (^{Tol}*m*-*C*H*)*, 124.47 (^{Dipp}*m*-*C*H), 122.86 (N-*C*H), 29.50 (*C*H), 25.46 (^{Dipp}*C*H$_3$), 22.84 (^{Dipp}*C*H$_3$), 21.58 (^{Tol}*C*H$_3$), 3.93 (*TMS*).

^{29}Si NMR (99 MHz, C$_6$D$_6$): δ 455.01 (d, J = 187.5 Hz, P-*Si*), -9.59 (d, J = 8.4 Hz, *TMS*), -10.73 (d, J = 19.1 Hz, *Si*Tol$_3$), -110.09 (d, J = 43.2 Hz, *Si*TMS$_2$SiTol$_3$).

^{31}P NMR (162 MHz, C$_6$D$_6$): δ 269.40.

m.p.: crystalline product: 139.3 – 141.4 °C decomposition (color change to brown)

with 15% Br-SiTMS$_2$SiTol$_3$: 138.0 – 143.9 °C (similar to pure compound)

LIFDI-MS (*m/z*): calcd.: 923.4536 [M +H]$^+$

found: 923.4565

Elemental Analysis (%):

calcd. for **2**: C: 72.43, N: 3.13, H: 8.44

found for **2**: C: 68.69, N: 3.00, H: 8.43

calcd. for **bulk 2 with 20% byproduct**: C: 70.08, N: 2.61, H: 8.21

found for **bulk 2 with 20% byproduct**: C: 66.27, N: 2.73, H: 7.94

Note: All samples containing **2/5** analyzed low for carbon (very likely due to minimal contamination with silicon grease).

Synthesis of (IDipp)PSiCO$_3$(SiTMS$_2$SiTol$_3$) (3):

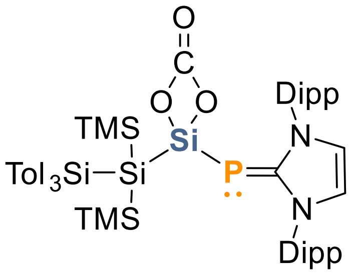

3

In a *J. Young* NMR tube (IDipp)PSi(SiTMS$_2$SiTol$_3$) (**2**, 50.0 mg, 54.14 µmol) was dissolved in benzene. The reaction mixture was frozen in liquid nitrogen and the vessel was evacuated briefly to remove the argon atmosphere. Without further cooling 1.0 bar of carbon dioxide (≥99.999%) was pressurized into the vessel. After closing it, the mixture was slowly warmed to room temperature. A colour change from dark brown to yellow was observed. All solvent was removed *in vacuo* after full conversion was confirmed *via* NMR spectroscopy and the residue was washed with hexane to give **3** as a yellow solid (36.6 mg, 69%). Storage of the hexane washing solution at -35 °C gave pale-orange crystals of **3** suitable for XRD analysis. Alternatively, crystals of **3** can be obtained from a diethyl ether solution slowly evaporating at -35 °C.

^{1}H NMR (400 MHz, C$_6$D$_6$): δ 7.73 (d, J = 7.6 Hz, 6H, ^{Tol}*o*-C*H*), 7.38 (t, J = 7.8 Hz, 2H, ^{Dipp}*p*-C*H*), 7.22 (d, J = 7.8 Hz, 4H, ^{Dipp}*m*-C*H*), 7.02 (d, J = 7.5 Hz, 6H, ^{Tol}*m*-C*H*), 6.26 (s, 2H, N-C*H*), 2.72 (hept, J = 6.9 Hz, 4H, C*H*), 2.14 (s, 9H, ^{Tol}C*H*$_3$), 1.50 (d, J = 6.8 Hz, 12H, *i*Pr-C*H*$_3$), 1.01 (d, J = 6.9 Hz, 12H, *i*Pr-C*H*$_3$), 0.24 (s, 18H, *TMS*).

^{13}C NMR (126 MHz, C$_6$D$_6$): δ 165.45 (d, J = 126.4 Hz, *C*=P), 149.57 (O=*C*OO), 145.65 (^{Dipp}Ar-*C*), 138.25 (^{Tol}*C*-Me), 136.86 (d, J = 2.3 Hz, ^{Tol}Ar-*C*H), 133.52 (^{Dipp}N-*C*), 133.23 (^{Tol}Si-*C*), 131.10 (^{Dipp}Ar-*C*H), 128.55 (^{Tol}Ar-*C*H), 124.67 (^{Dipp}Ar-*C*H), 122.71 (d, J = 3.4 Hz, N-*C*H), 29.06 (*C*H), 25.08 (^{Dipp}*C*H$_3$), 22.68 (^{Dipp}*C*H$_3$), 21.19 (^{Tol}*C*H$_3$), 2.68 (d, J = 2.9 Hz, *TMS*).

^{29}Si NMR (99 MHz, C$_6$D$_6$): δ 41.01 (d, J = 201.6 Hz, P-*Si*), -8.87 (d, J = 7.8 Hz, *TMS*), -12.66 (d, J = 5.0 Hz, *Si*Tol$_3$), -125.15 (d, J = 39.2 Hz, *Si*TMS$_2$SiTol$_3$).

^{31}P NMR (162 MHz, C$_6$D$_6$): δ -147.35.

m.p.: 85.3 – 87.5 °C decomposition (dark-brown residue, no visible gas evolution)

LIFDI-MS: calcd.: 983.4383 [M +H]$^+$

found: 983.4311

Synthesis of (IDipp)PSiC_2H_4(SiTMS$_2$SiTol$_3$) (4):

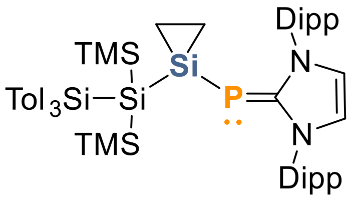

4

In a *J. Young* NMR tube (IDipp)PSi(SiTMS$_2$SiTol$_3$) (**2**, 50.0 mg, 54.14 µmol) was dissolved in benzene. The reaction mixture was frozen in liquid nitrogen and the vessel was evacuated briefly to remove the argon atmosphere. Without further cooling 1.0 bar of ethylene (≥99.95%) was pressurized into the vessel. After closing it, the mixture was slowly warmed to room temperature. A colour change from dark brown to orange was observed. Full conversion was confirmed *via* NMR spectroscopy. Subsequently, benzene was removed *in vacuo* and the residue was washed with hexane to give **4** as an orange solid (21.5 mg, 42%). Storage of the hexane washing solution at -35 °C gave brown crystals of **4** suitable for XRD analysis. Crystals of **4** could also be obtained from a diethyl ether solution slowly evaporating at -35 °C instead.

Note: 4 is not indefinitely stable in solution and slowly decomposes into multiple unidentified species after few days.

^{1}H NMR (500 MHz, C_6D_6): δ 7.76 (d, J = 7.9 Hz, 6H, ^{Tol}*o*-C*H*), 7.23 (t, 2H, ^{Dipp}*p*-C*H*), 7.13 (d, J = 7.7 Hz, 4H, ^{Dipp}*m*-C*H*), 7.02 (d, J = 7.6 Hz, 6H, ^{Tol}*m*-C*H*), 6.02 (s, 2H, N-C*H*), 3.00 (hept, J = 6.8 Hz, 4H, C*H*), 2.08 (s, 9H, ^{Tol}CH_3), 1.50 (d, J = 6.9 Hz, 12H, *i*Pr-CH_3), 1.08 (d, J = 6.9 Hz, 12H, *i*Pr-CH_3),), 0.37 (s, 18H, *TMS*), 0.29 – 0.23 (m, 4H, CH_2-CH_2).

^{13}C NMR (126 MHz, C_6D_6): δ 169.61 (d, J = 134.3 Hz, *C*=P), 146.26 (^{Dipp}Ar-*C*), 138.078(^{Tol}*C*-Me), 136.94 (^{Tol}Ar-*C*H), 134.97 (^{Dipp}N-*C*), 134.36 (^{Tol}Si-*C*), 130.18 (^{Dipp}Ar-*C*H), 128.37 (^{Tol}Ar-*C*H), 124.58 (^{Dipp}Ar-*C*H), 120.48 (d, J = 3.4 Hz, N-*C*H), 28.74 (*C*H), 24.40 (^{Dipp}*C*H_3), 23.14 (^{Dipp}*C*H_3), 21.12 (^{Tol}*C*H_3), 3.71 (d, J = 4.8 Hz, *TMS*).

^{29}Si NMR (99 MHz, C_6D_6): δ -8.94 (d, J = 4.3 Hz, *TMS*), -11.33 (d, J = 15.5 Hz, *Si*Tol$_3$), -107.10 (d, J = 192.0 Hz, P-*Si*), -124.29 (d, J = 51.5 Hz, *Si*TMS$_2$SiTol$_3$).

^{31}P NMR (162 MHz, C_6D_6): δ -166.98.

m.p.: 68.0 – 70.8 °C decomposition (little change of colour)

LIFDI-MS (*m/z*): calcd.: 950.4827 [M]$^+$

found: 950.4745

Synthesis of (IDipp)SiP($SiTMS_2SiTol_3$) (5):

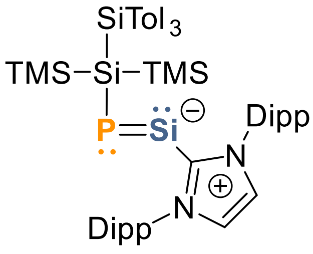

5

(IDipp)PSi($SiTMS_2SiTol_3$) (**2**, 50.0 mg, 54.14 µmol) was dissolved in toluene and heated to 80 °C for one hour. The conversion also takes place at room temperature but is very slow (66% after 14 days according to NMR). After the complete formation of **5**, the former brown and non-transparent solution appears clear and red. Evaporating the solvent *in vacuo* yields **5** as a red-brown solid containing small amounts of unidentified impurities visible in the ^{31}P NMR (Impurity formation can be partially avoided with longer reaction time and lower temperature). $BrSiTMS_2SiTol_3$ within the starting material remains in the product. Crystals of **5** suitable for XRD analysis could be obtained from an evaporating diethyl ether solution (crystalline yield: 32%) at room temperature similar to compound **2**.

^{1}H NMR (500 MHz, C_6D_6): δ 7.82 (d, J = 8.0 Hz, 6H, ^{Tol}*o*-C*H*), 7.24 (t, J = 7.8 Hz, 2H, ^{Dipp}*p*-C*H*), 7.08 (d, J = 7.8 Hz, 4H, ^{Dipp}*m*-C*H*), 7.05 (d, J = 7.3 Hz, 6H, ^{Tol}*m*-C*H*), 6.53 (s, 2H, N-C*H*), 3.00 (hept, 4H, C*H*), 2.17 (s, 9H, ^{Tol}C*H*$_3$), 1.35 (d, J = 6.8 Hz, 12H, *i*Pr-C*H*$_3$), 1.06 (d, J = 6.9 Hz, 12H, *i*Pr-C*H*$_3$), 0.27 (s, 18H, *TMS*).

^{13}C NMR (126 MHz, C_6D_6): δ 181.05 (d, J = 17.0 Hz, *C*-Si), 145.87 (^{Dipp}Ar*C*), 137.62 (^{Tol}*o*-*C*H), 136.86 (^{Tol}*C*-Me), 135.74 (^{Tol}Si-*C*), 133.45 (^{Dipp}N-*C*), 130.97 (^{Dipp}*p*-*C*H), 128.52 (^{Tol}*m*-*C*H), 124.44 (^{Dipp}*m*-*C*H), 123.80 (N-*C*H), 29.46 (*C*H), 25.79 (^{Dipp}*C*H$_3$), 22.87 (^{Dipp}*C*H$_3$), 21.63 (^{Tol}*C*H$_3$), 2.76 (*TMS*).

^{29}Si NMR (99 MHz, C_6D_6): δ 337.64 (d, J = 182.4 Hz, P-*Si*), -11.51 (*TMS*), -14.93 (*Si*Tol$_3$), -87.93 (d, J = 97.8 Hz, *Si*TMS$_2$SiTol$_3$).

^{31}P NMR (162 MHz, C_6D_6): δ 270.43.

m.p.: with 15% Br-$SiTMS_2SiTol_3$: 139.7 – 142.2 °C decomposition (gradually turns dark brown)

LIFDI-MS (*m/z*): calcd.: 922.4514 $[M]^+$

found: 922.4447

Synthesis of (IDipp)PSi[Fe(CO$_4$)](SiTMS$_2$SiTol$_3$) (6):

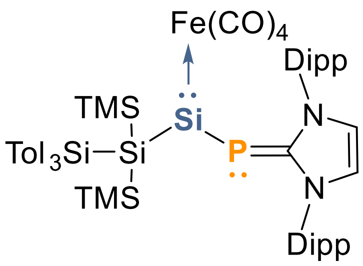

6

In a *Schlenk* tube (IDipp)PSi(SiTMS$_2$SiTol$_3$) (**2**, 50.0 mg, 54.14 µmol, 1.0 eq.) was dissolved in toluene and iron pentacarbonyl (7.68 µL, 56.84 µmol, 1.05 eq.) was added. A colour change from dark brown to orange was observed and the reaction mixture was stirred for about four hours. Full conversion was confirmed *via* ^{31}P NMR spectroscopy. The solvent was removed *in vacuo* and the residue was washed with pentane to give **6** as a yellow solid (29.0 mg, 48%). Crystals of **6** suitable for XRD analysis were obtained from a saturated diethyl ether solution after prolonged storage at -35 °C.

^{1}H NMR (500 MHz, C$_6$D$_6$): δ 7.75 (d, J = 7.5 Hz, 6H, ^{Tol}*o*-C*H*), 7.23 (t, J = 7.7 Hz, 2H, ^{Dipp}*p*-C*H*), 7.02 (d, J = 7.7 Hz, 6H, ^{Tol}*m*-C*H*), 6.48 (s, 2H, N-C*H*), 3.65 (br, s, 2H, C*H*), 2.26 (br, s, 2H, C*H*), 2.14 (s, 9H, ^{Tol}C*H*$_3$), 1.59 (br, s, 6H, *i*Pr-C*H*$_3$), 1.09 (s, 12H, *i*Pr-C*H*$_3$), 0.79 (s, 6H, *i*Pr-C*H*$_3$), 0.36 (s, 19H, *TMS*).

Note: Signal of ^{Dipp}*m*-CH is not visible due to broadening and overlap with the solvent signal.

^{13}C NMR (126 MHz, C$_6$D$_6$): δ 215.13 (*C*O), 166.91 (d, J = 133.8 Hz, *C*=P), 145.40 (br, d, J = 74.5 Hz, ^{Dipp}Ar*C*), 138.08 (^{Tol}*C*-Me), 137.61 (^{Tol}*o*-*C*H), 134.10 (^{Tol}Si-*C*), 133.45 (^{Dipp}N-*C*), 130.77 (^{Dipp}*p*-*C*H), 128.31 (^{Tol}*m*-*C*H*)*, 124.59 (N-*C*H), 124.53 (br, d, J = 73.2 Hz, ^{Dipp}*m*-*C*H), 28.49 (br, d, J = 73.6 Hz, *C*H), 25.84 (br, d, J = 114.0 Hz, ^{Dipp}*C*H$_3$), 22.65 (br, d, J = 155.5 Hz, ^{Dipp}*C*H$_3$), 21.22 (^{Tol}*C*H$_3$), 3.75 (*TMS*).

^{29}Si NMR (99 MHz, C$_6$D$_6$): δ 349.47 (d, J = 193.0 Hz, P-*Si*), -8.38 (d, J = 14.2 Hz, *Si*Tol$_3$), -9.24 (d, J = 11.3 Hz, *TMS*), -96.97 (d, J = 56.2 Hz, *Si*TMS$_2$SiTol$_3$).

^{31}P NMR (203 MHz, C$_6$D$_6$): δ 153.18.

m.p.: 179.9 – 183.6 °C decomposition (gas evolution)

LIFDI-MS (*m/z*): calcd.: 1062.3049 [M -CO]$^+$

found: 1062.3712

Synthesis of (IDipp)Si[Fe(CO_4)]P(SiTMS$_2$SiTol$_3$) (7):

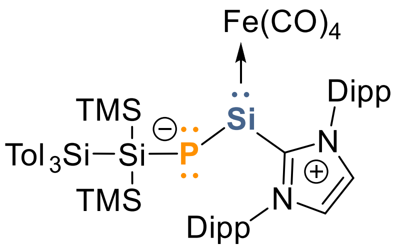

7

In a *Schlenk* tube (IDipp)SiP(SiTMS$_2$SiTol$_3$) (**5**, 50.0 mg, 54.14 µmol, 1.0 eq.) was dissolved in toluene. Iron pentacarbonyl (21.94 µL, 162.4 µmol, 3.0 eq.) was added. No colour change was observed. Subsequently, the reaction mixture was stirred at 80 °C for twelve hours until full conversion was confirmed *via* ^{31}P NMR spectroscopy. The solvent and excess iron pentacarbonyl were removed *in vacuo* and the residue was washed with pentane to give **7** as an orange solid (35.8 mg, 59%). Crystals of **7** suitable for XRD analysis were obtained from pentane washing solutions stored at -35 °C.

^{1}H NMR (500 MHz, C_6D_6): δ 7.73 (d, J = 8.0 Hz, 6H, ^{Tol}*o*-C*H*), 7.31 (t, J = 7.8 Hz, 2H, ^{Dipp}*p*-C*H*), 7.10 (d, J = 7.6 Hz, 6H, ^{Tol}*m*-C*H*), 6.45 (s, 2H, N-C*H*), 3.41 (br, s, 2H, C*H*), 2.80 (br, s, 2H, C*H*), 2.20 (s, 9H, ^{Tol}C*H*$_3$), 1.59 (br, s, 6H, *i*Pr-C*H*$_3$), 1.20 (br, s, 6H, *i*Pr-C*H*$_3$), 1.03 (br, s, 6H, *i*Pr-C*H*$_3$), 0.83 (br, s, 6H, *i*Pr-C*H*$_3$), 0.37 (s, 18H, *TMS*).

^{13}C NMR (126 MHz, C_6D_6): δ 215.28 (*C*O), 165.64 (d, J = 42.4 Hz, Si-*C*), 145.92 (d, J = 116.4 Hz, ^{Dipp}Ar*C*), 138.38 (^{Tol}*C*-Me), 137.65 (^{Tol}*o*-*C*H), 135.16(^{Tol}Si-*C*), 133.47(^{Dipp}N-*C*), 131.71(^{Dipp}*p*-*C*H), 128.78 (^{Tol}*m*-*C*H*)*, 125.10 (^{Dipp}*m*-*C*H), 124.73 (N-*C*H), 29.27 (br, d, J = 89.0 Hz, *C*H), 26.67 (^{Dipp}*C*H$_3$), 22.47 (^{Dipp}*C*H$_3$), 21.60 (^{Tol}*C*H$_3$), 3.61 (*TMS*).

^{29}Si NMR (99 MHz, C_6D_6): δ -9.58 (*TMS*), -86.07 (d, J = 117.4 Hz, *Si*TMS$_2$SiTol$_3$).

^{29}Si NMR (80 MHz, Tol-*d*8, 243K): δ 240.90 (d, *J* = 160.5 Hz, *Si*-P), -9.66 (*TMS*), -12.71 (d, *J* = 21.6 Hz, *Si*Tol$_3$), -87.99 (d, *J* = 113.9 Hz, *Si*TMS$_2$SiTol$_3$).

^{31}P NMR (203 MHz, C_6D_6): δ 189.71.

m.p.: 202.7 – 205.2 °C decomposition (gas evolution)

LIFDI-MS (*m/z*): calcd.: 1062.3049 [M -CO]$^+$

found: 1062.3712

Synthesis of (IDipp)PSi[PhC$_2$H](SiTMS$_2$SiTol$_3$) / (IDipp)PSi[C$_4$H$_6$](SiTMS$_2$SiTol$_3$) (8a)(8b):

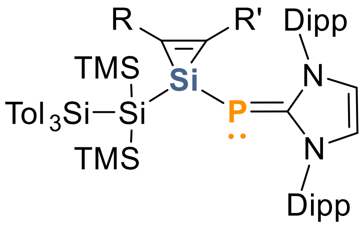

8a (R = Ph, R' = H)
8b (R = R' = Me)

In a *Schlenk* tube (IDipp)PSi(SiTMS$_2$SiTol$_3$) (**2**, 50.0 mg, 54.14 µmol) was dissolved in toluene. Phenyl acetylene (6.06 µL, 55.2 mmol, 1.02 eq.) or dimethyl acetylene (4.33 µL, 55.2 mmol, 1.02 eq.) was added. A rapid color change from dark brown to orange was observed and the reaction mixture was stirred for about four hours. Full conversion was confirmed *via* ^{31}P NMR spectroscopy. The solvent was removed *in vacuo*.

8a: Isolation of **8a** is more difficult due to its higher solubility in hexane/pentane. The residue can be washed with pentane after thorough drying to give **8a** as a yellow solid (16.8 mg, 31%) in smaller yield. Crystals of **8a** suitable for XRD analysis were obtained from a toluene solution after prolonged storage at -35 °C.

Note: Compound **8a** is not indefinitely stable in solution and starts to slowly decompose into multiple unidentified species after a few days.

^{1}H NMR (500 MHz, C$_6$D$_6$): δ 7.66 (d, J = 7.8 Hz, 6H, ^{Tol}*o*-C*H*), 7.52 (s, 1H, Si-C*H*), 7.33 – 7.10 (m, 9H, *p*-Ph*H, m*-Ph*H,* ^{Dipp}*p-CH,* ^{Dipp}*m-CH*), 6.97 (d, J = 7.6 Hz, 6H, ^{Tol}*m*-C*H*), 6.86 (dd, J = 6.8, 2.4 Hz, 2H, *m*-Ph*H*), 5.99 (s, 2H, N-C*H*), 3.16 (hept, J = 6.9 Hz, 2H, C*H*), 2.70 (hept, J = 6.9 Hz, 2H, C*H*), 2.13 (s, 9H, ^{Tol}C*H*$_3$), 1.64 (d, J = 6.8 Hz, 6H, *i*Pr-C*H*$_3$), 1.28 (d, J = 7.0 Hz, 6H, *i*Pr-C*H*$_3$), 1.09 (d, J = 6.9 Hz, 6H, *i*Pr-C*H*$_3$), 1.01 (d, J = 6.9 Hz, 6H, *i*Pr-C*H*$_3$), 0.40 (s, 9H, *TMS*), 0.28 (s, 9H, *TMS*).

^{13}C NMR (126 MHz, C$_6$D$_6$): δ 169.79 (d, J = 135.5 Hz, *C*=P), 162.13 (Si*C*Ph), 146.26 (^{Dipp}Ar*C*), 145.98 (^{Dipp}Ar*C*), 145.43 (Si*C*H), 138.08 (^{Tol}*C*-Me), 137.41 (^{Tol}*o*-*C*H), 135.09 (^{Dipp}N-*C*), 134.85 (^{Tol}Si-*C*), 134.22 (^{Ph}*i-C*), 132.39, 130.20, 130.11, 128.35 (^{Tol}*m*-*C*H), 125.15 (^{Ph}*o*-*C*H), 124.81 (^{Dipp}*m*-*C*H), 120.81 (d, J = 3.4 Hz, ^{Dipp}N-*C*), 29.30 (*C*H), 28.94 (*C*H), 24.89 (^{Dipp}*C*H$_3$), 24.17 (^{Dipp}*C*H$_3$), 24.02 (^{Dipp}*C*H$_3$), 23.19 (^{Dipp}*C*H$_3$), 21.55 (Tol-*C*H$_3$), 3.95 (d, J = 4.3 Hz, *TMS*), 3.80 (d, J = 4.7 Hz, *TMS*).

^{29}Si NMR (99 MHz, C_6D_6): δ -8.85 (d, J = 8.0 Hz, *TMS*), -9.90 (d, J = 2.3 Hz, *TMS*), -311.05 (d, J = 15.1 Hz, *Si*Tol_3), -103.62 (d, J = 202.8 Hz, P-*Si*), -121.50 (d, J = 2.3 Hz, *Si*TMS_2SiTol_3).

^{31}P NMR (162 MHz, C_6D_6): δ -154.78.

m.p.: 70.5 – 71.8 °C (**NOTE**: sample contained 10% $BrSiTMS_2SiTol_3$)

LIFDI-MS (*m/z*): calcd.: 1024.4983 $[M]^+$

found: 1024.4950

8b: The residue was washed with pentane to give **8b** as a pale yellow solid (31.0 mg, 58%). Crystals of **8b** suitable for XRD analysis were obtained from a saturated diethyl ether solution after prolonged storage at -35 °C.

Note: Compound **8b** is not indefinitely stable in solution and starts to slowly decompose into multiple unidentified species after a few days.

^{1}H NMR (500 MHz, C_6D_6): δ 7.69 (d, J = 7.9 Hz, 6H, ^{Tol}*o*-C*H*), 7.18 (t, J = 7.8 Hz, 2H, ^{Dipp}*p*-C*H*), 7.07 (dd, J = 7.6, 1.2 Hz, 10H, ^{Dipp}*m*-C*H*, ^{Tol}*m*-C*H*), 6.00 (s, 2H, N-C*H*), 2.95 (hept, J = 6.9 Hz, 4H, C*H*), 2.15 (s, 9H, ^{Tol}CH_3), 1.68 (s, 6H, SiC-CH_3), 1.59 (d, J = 6.9 Hz, 12H, *i*Pr-CH_3), 1.12 (d, J = 6.9 Hz, 12H, *i*Pr-CH_3), 0.31 (s, 18H, *TMS*).

^{13}C NMR (126 MHz, C_6D_6): δ 169.71 (d, J = 141.6 Hz, *C*=P), 156.91 (Si*C*-CH_3), 146.29 (^{Dipp}Ar-*C*), 138.06 (^{Tol}*C*-Me), 137.43 (^{Tol}*o*-*C*H), 135.70 (^{Tol}Si-*C*), 135.14 (^{Dipp}N-*C*), 129.83 (^{Dipp}Ar-*C*H), 128.60 (^{Tol}*m*-*C*H), 125.27 (^{Dipp}Ar-*C*H), 120.36 (N-*C*H), 29.30 (*C*H), 24.48 (^{Dipp}*C*H_3), 23.44 (^{Dipp}*C*H_3), 21.52 (^{Tol}*C*H_3), 14.50 (SiC-*C*H_3), 4.06 (d, J = 5.1 Hz, *TMS*).

^{29}Si NMR (99 MHz, C_6D_6): δ -9.62 (d, J = 6.4 Hz, *TMS*), -10.19 (d, J = 21.4 Hz, *Si*Tol_3), -101.60 (d, J = 208.8 Hz, P-*Si*), -122.01 (d, J = 90.0 Hz, *Si*TMS_2SiTol_3).

^{31}P NMR (162 MHz, C_6D_6): δ -155.24.

m.p.: 158.8 – 161.1 °C (little change of colour)

LIFDI-MS (*m/z*): calcd.: 976.4984 $[M]^+$

found: 950.4903

Synthesis of (IDipp)Si[PhC$_2$H]P(SiTMS$_2$SiTol$_3$) / (IDipp)Si[C$_4$H$_6$]P(SiTMS$_2$SiTol$_3$) (9a)(9b):

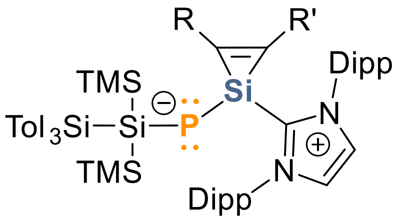

9a (R = Ph, R' = H)
9b (R = R' = Me)

In a *Schlenk* tube (IDipp)SiP(SiTMS$_2$SiTol$_3$) (**5**, 50.0 mg, 54.14 µmol) was dissolved in toluene. Phenyl acetylene (6.06 µL, 55.2 mmol, 1.02 eq.) or dimethyl acetylene (4.33 µL, 55.2 mmol, 1.02 eq.) was added. No color change was observed and the reaction mixture was stirred overnight (16h). Full conversion was confirmed *via* ^{31}P NMR spectroscopy. Subsequently, the solvent was removed *in vacuo*.

9a: The residue was washed with pentane to give **9a** as an orange-red solid (22.2 mg, 37%). Crystals of **9a** suitable for XRD analysis were obtained from a saturated diethyl ether solution after prolonged storage at -35 °C.

^{1}H NMR (500 MHz, C$_6$D$_6$): δ 8.23 (d, J = 2.4 Hz, 1H, SiC-*H*), 7.84 (d, J = 7.9 Hz, 6H, ^{Tol}*o*-C*H*), 7.32 (t, J = 7.8 Hz, 2H, ^{Dipp}*p*-C*H*), 7.25 (dd, J = 7.8, 1.5 Hz, 2H, ^{Dipp}*m*-C*H*), 7.09 – 7.03 (m, 9H, ^{Tol}*o*-C*H*, Ph*H*), 7.02 (dd, J = 7.8, 1.5 Hz, 2H, ^{Dipp}*m*-C*H*), 6.83 (dd, J = 7.5, 1.9 Hz, 2H, Ph*H*), 6.25 (s, 2H, N-C*H*), 3.09 (hept, J = 6.7 Hz, 2H, C*H*), 2.49 (hept, J = 6.8 Hz, 2H, C*H*), 2.19 (s, 9H, ^{Tol}C*H*$_3$), 1.57 (d, J = 6.6 Hz, 6H, *i*Pr-C*H*$_3$), 1.11 (d, J = 6.7 Hz, 6H, *i*Pr-C*H*$_3$), 0.89 (dd, J = 6.9, 2.0 Hz, 12H, *i*Pr-C*H*$_3$), 0.25 (s, 9H, *TMS*), 0.18 (s, 9H, *TMS*).

^{13}C NMR (126 MHz, C$_6$D$_6$): δ 169.94 (d, J = 4.9 Hz, Si-*C*Ph), 162.20 (d, J = 37.6 Hz, Si-*C*N), 150.77 (d, J = 4.9 Hz, Si-*C*H), 146.16 (^{Dipp}Ar-*C*), 145.80 (^{Dipp}Ar-*C*), 137.73 (^{Tol}*o*-CH), 137.45 (^{Tol}*C*-Me), 136.37 (^{Tol}Si-*C*), 134.51 (*Ph*), 134.04 (^{Dipp}N-*C*), 131.23 (^{Dipp}*p*-CH), 129.36 (*Ph*), 128.33 (^{Tol}*m*-CH), 125.03 (^{Dipp}*m*-CH), 124.60 (^{Dipp}*m*-CH), 124.00 (*Ph*), 123.70 (N-*C*H), 29.28 (t, J = 3.1 Hz, *C*H), 26.37 (^{Tol}*C*H$_3$), 25.98 (^{Tol}*C*H$_3$), 23.05 (d, J = 2.9 Hz, ^{Tol}*C*H$_3$), 22.52 (d, J = 5.4 Hz, ^{Tol}*C*H$_3$), 21.63, 2.51 (*TMS*), 2.38 (*TMS*).

^{29}Si NMR (99 MHz, C$_6$D$_6$): δ -12.06 (d, J = 11.9 Hz, *TMS*), -12.97 (d, J = 3.6 Hz, *TMS*), -15.70 (d, J = 19.8 Hz, *Si*Tol$_3$), -88.78 (d, J = 182.4 Hz, P-*Si*), -92.87 (d, J = 114.7 Hz, *Si*TMS$_2$SiTol$_3$).

^{31}P NMR (162 MHz, C_6D_6): δ -372.38.

m.p.: 184.2 – 186.4 °C decomposition (colour change to dark brown)

LIFDI-MS (*m/z*): calcd.: 1025.5062 $[M+H]^+$

found: 1025.4989

9b: The residue was washed with pentane to give **9b** as an orange-red solid (29.9 mg, 56%). Crystals of **9b** suitable for XRD analysis were obtained from a pentane washing solution stored at -35 °C.

^{1}H NMR (500 MHz, C_6D_6): δ 7.92 (d, J = 7.9 Hz, 6H, ^{Tol}*o*-C*H*), 7.30 (t, J = 7.7 Hz, 2H, ^{Dipp}*p*-C*H*), 7.20 – 7.11 (m, 10H, ^{Dipp}*m*-C*H*, ^{Tol}*m*-C*H*), 6.17 (s, 2H, N-C*H*), 2.80 (hept, J = 6.8 Hz, 4H, C*H*), 2.21 (s, 9H, ^{Tol}CH_3), 1.52 (s, 6H, SiC-CH_3), 1.41 (d, J = 6.7 Hz, 12H, *i*Pr-CH_3), 0.93 (d, J = 6.9 Hz, 12H, *i*Pr-CH_3), 0.30 (s, 18H, *TMS*).

^{13}C NMR (126 MHz, C_6D_6): δ 163.35 (d, J = 40.6 Hz, Si-*C*), 160.00 (d, J = 5.0 Hz, Si*C*-CH_3), 145.35 (^{Dipp}Ar-*C*), 137.79 (d, J = 2.3 Hz, ^{Tol}*o*-*C*H), 137.44 (^{Tol}*C*-Me), 136.68 (^{Tol}Si-*C*), 134.70 (^{Dipp}N-*C*), 130.81 (^{Dipp}*p*-*C*H), 124.68 (^{Dipp}Ar-*C*H), 123.81 (N-*C*H), 29.12 (d, J = 2.0 Hz, *C*H), 25.69 (^{Dipp}*C*H_3), 23.01 (d, J = 4.4 Hz, ^{Dipp}*C*H_3), 21.66 (^{Tol}*C*H_3), 13.83 (SiC-*C*H_3), 2.42 (*TMS*).

^{29}Si NMR (99 MHz, C_6D_6): δ -12.74 (d, J = 4.7 Hz, *TMS*), -15.66 (d, J = 25.8 Hz, *Si*Tol_3), -89.16 (d, J = 180.4 Hz, P-*Si*), -93.39 (d, J = 117.9 Hz, *Si*TMS_2SiTol_3).

^{31}P NMR (162 MHz, C_6D_6): δ -376.55.

m.p.: 195.8 – 197.9 °C

LIFDI-MS (*m/z*): calcd.: 977.5062 $[M+H]^+$

found: 977.4982

3. Spectra

NMR

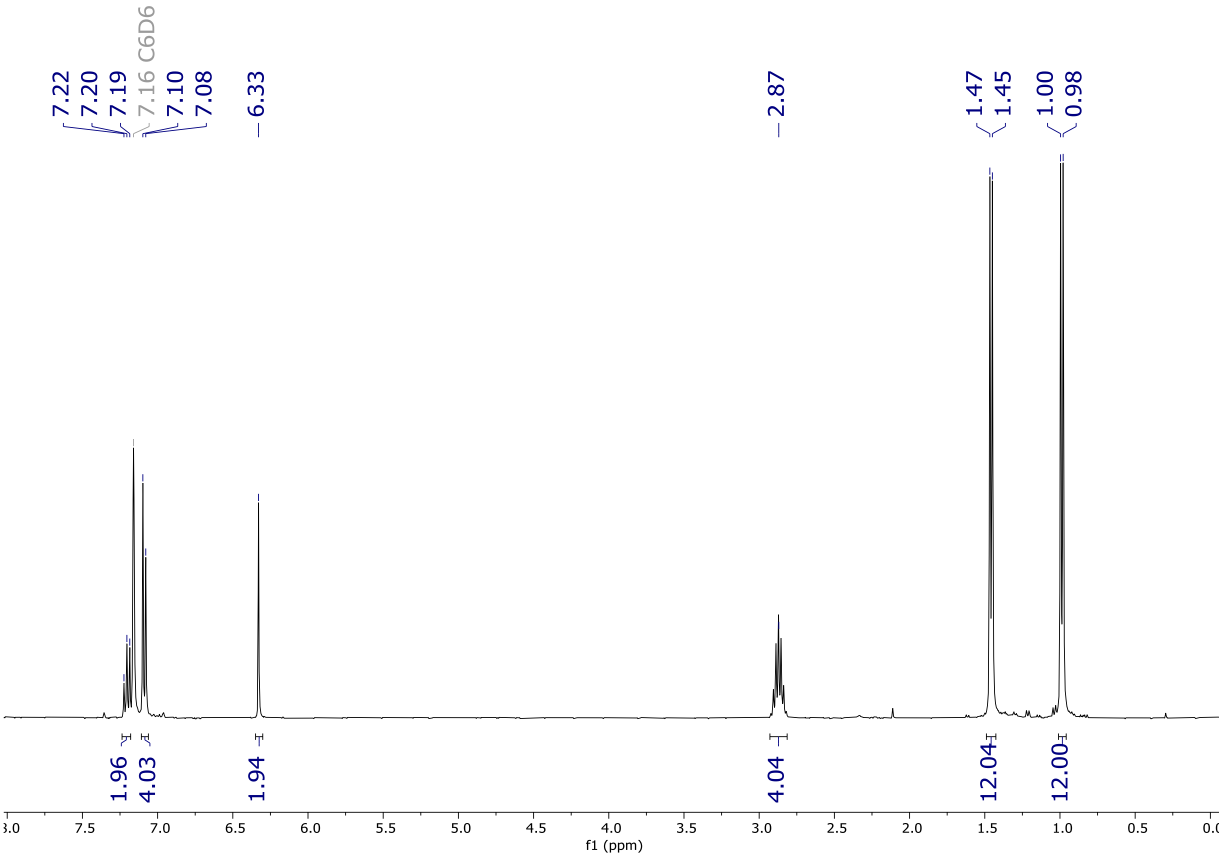

Supplementary Figure 1: ^{1}H NMR spectrum of **1** (C_6D_6).

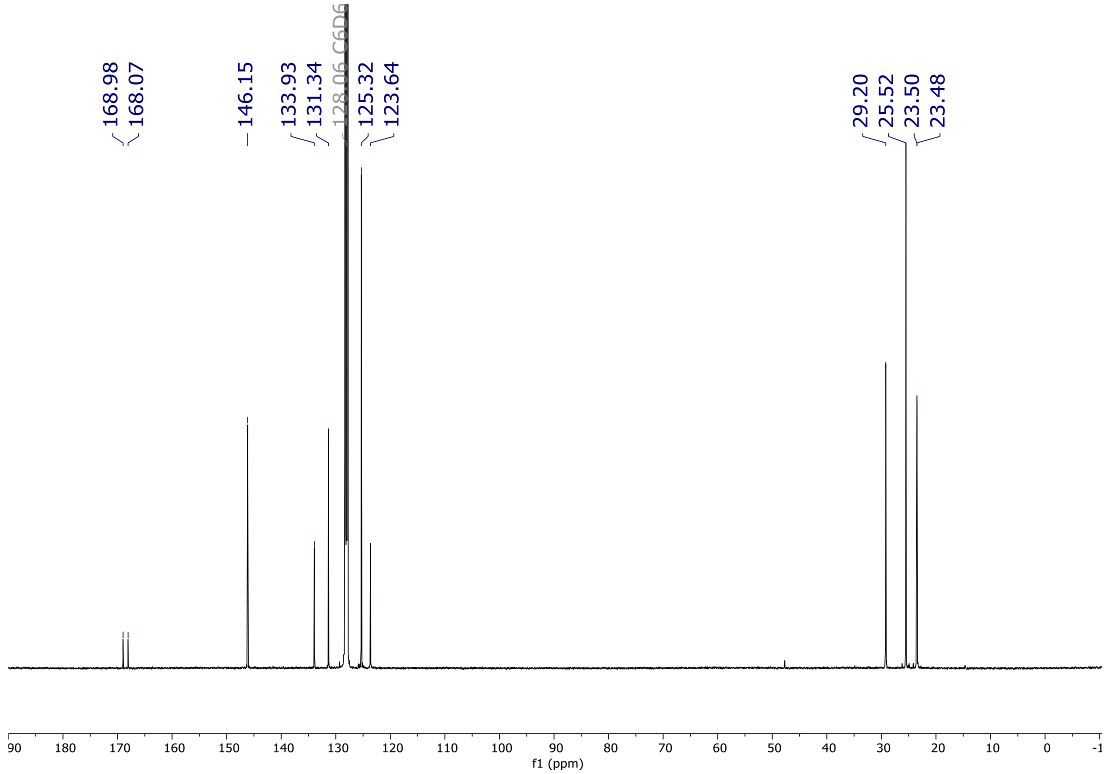

Supplementary Figure 2: $^{13}C\{^{1}H\}$ NMR spectrum of **1** (C_6D_6).

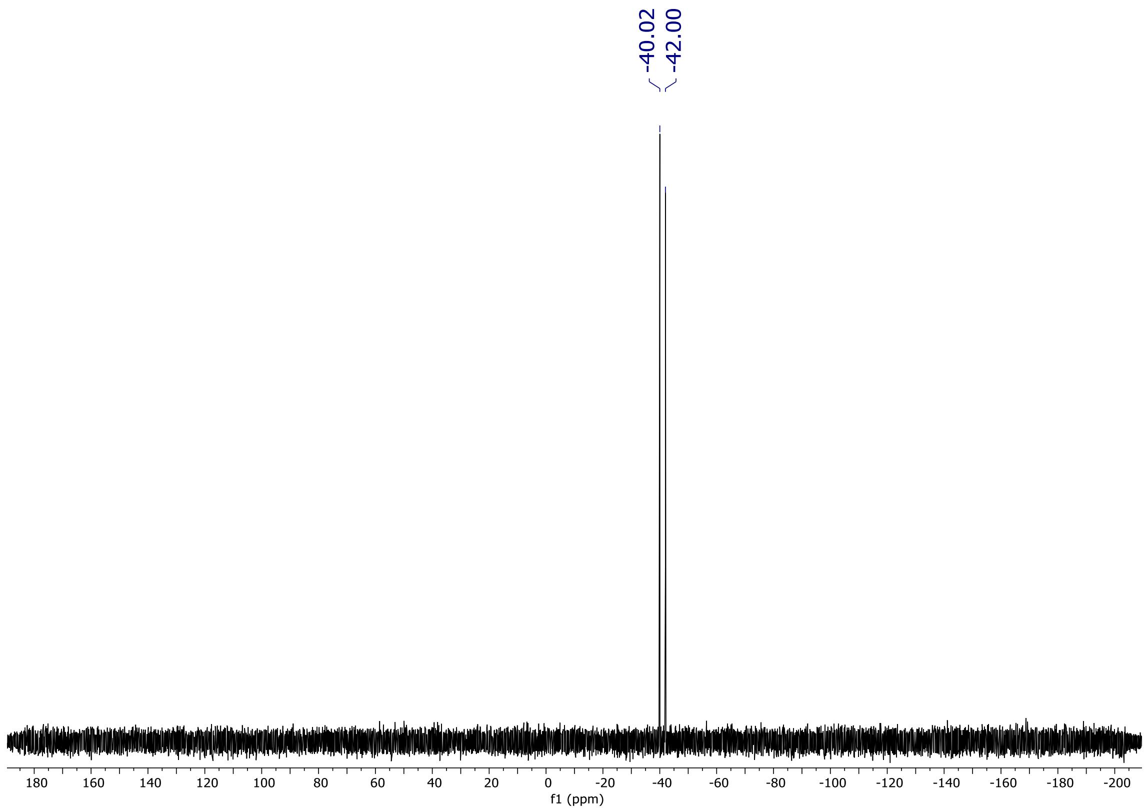

Supplementary Figure 3: ^{29}Si spectrum of **1** (C_6D_6).

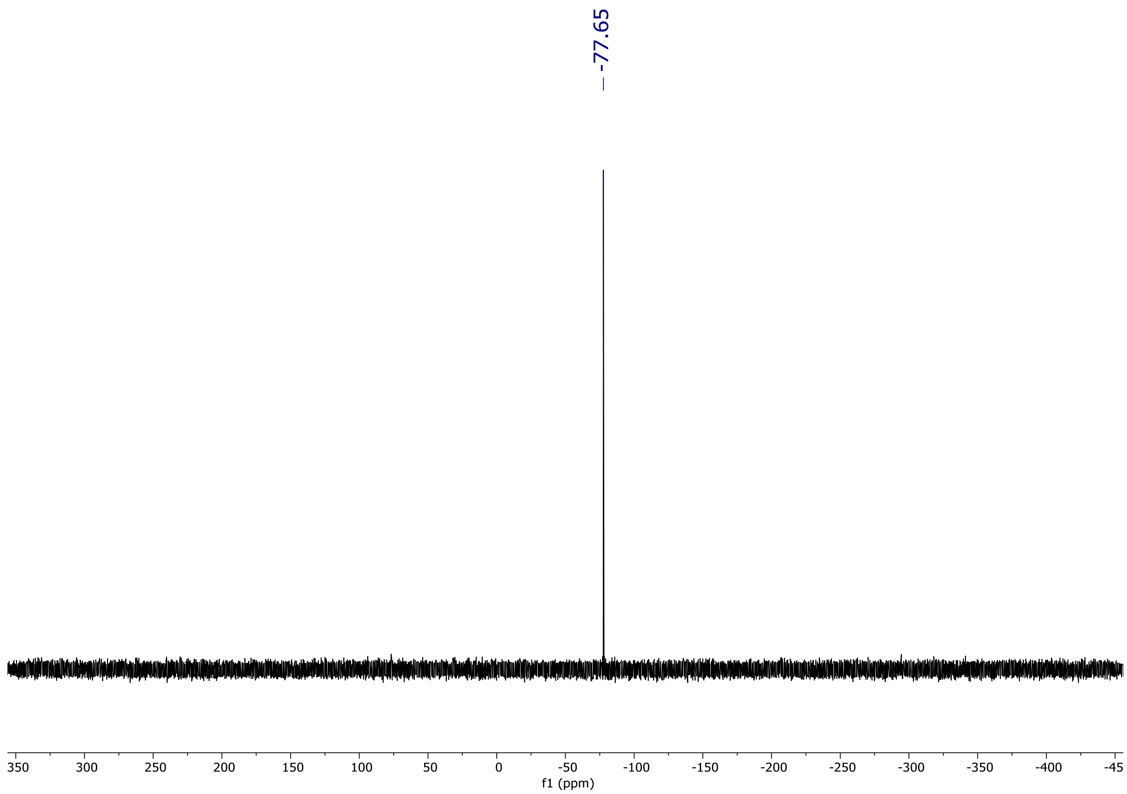

Supplementary Figure 4: $^{31}P\{^{1}H\}$ NMR spectrum of **1** (C_6D_6).

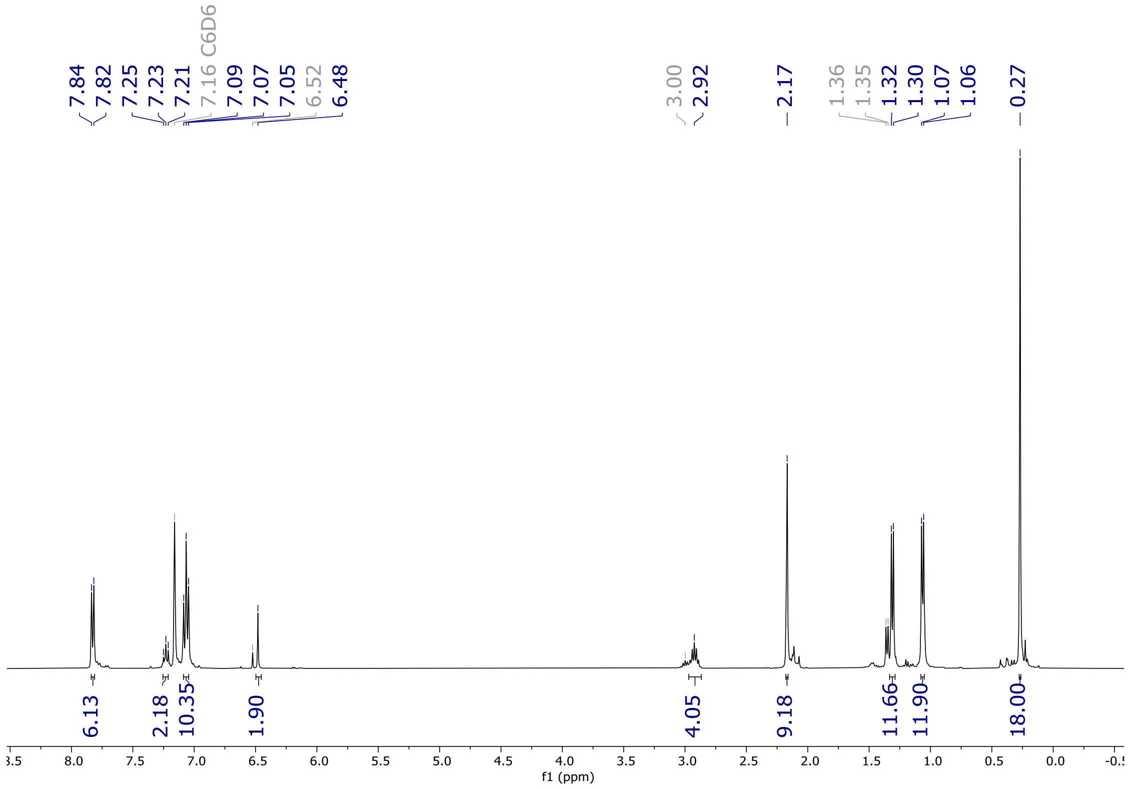

Supplementary Figure 5: ^{1}H NMR spectrum of **2** (C_6D_6). Little **5** has already formed (grey).

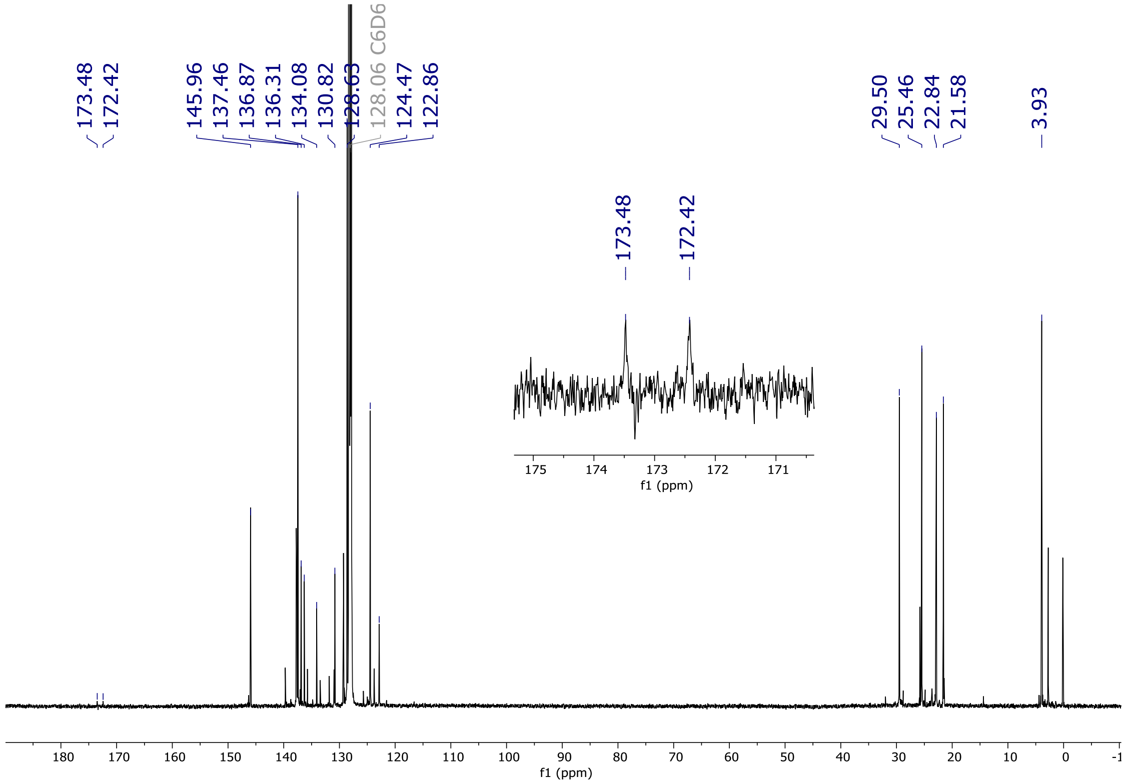

Supplementary Figure 6: ^{13}C NMR spectrum of **2** (bulk with side product due to low sensitivity!) (C_6D_6).

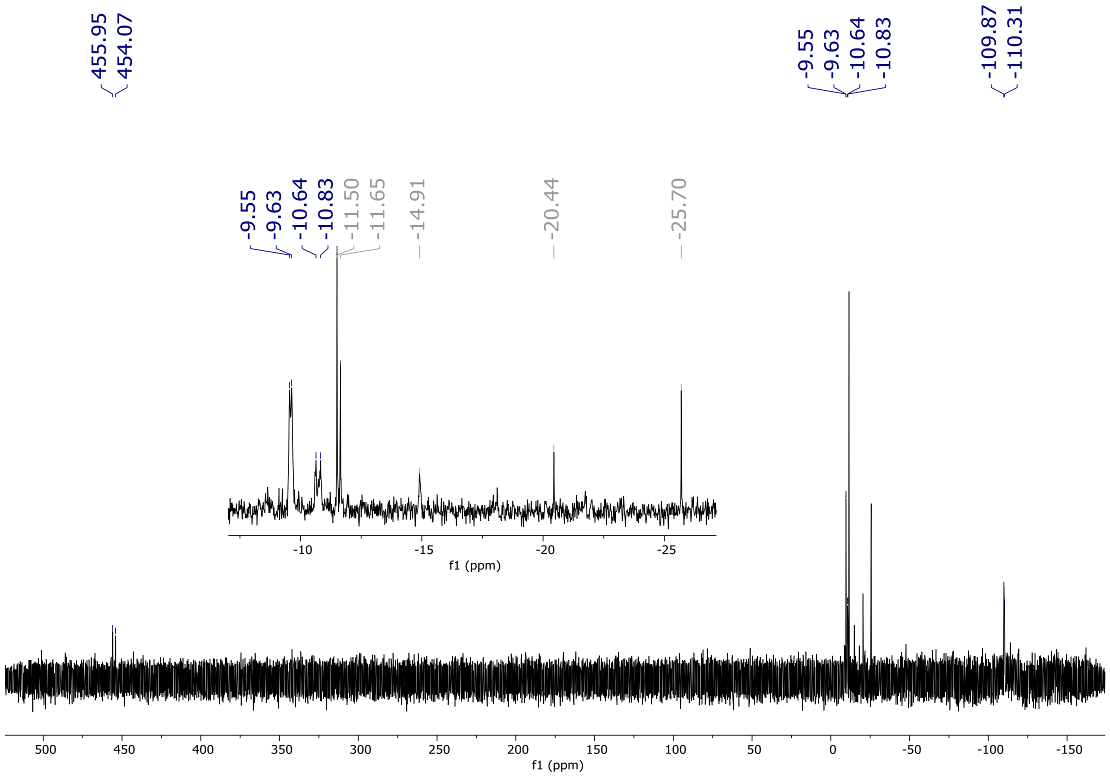

Supplementary Figure 7: ^{29}Si NMR spectrum of **2** (bulk with side product due to low sensitivity!) (C_6D_6).

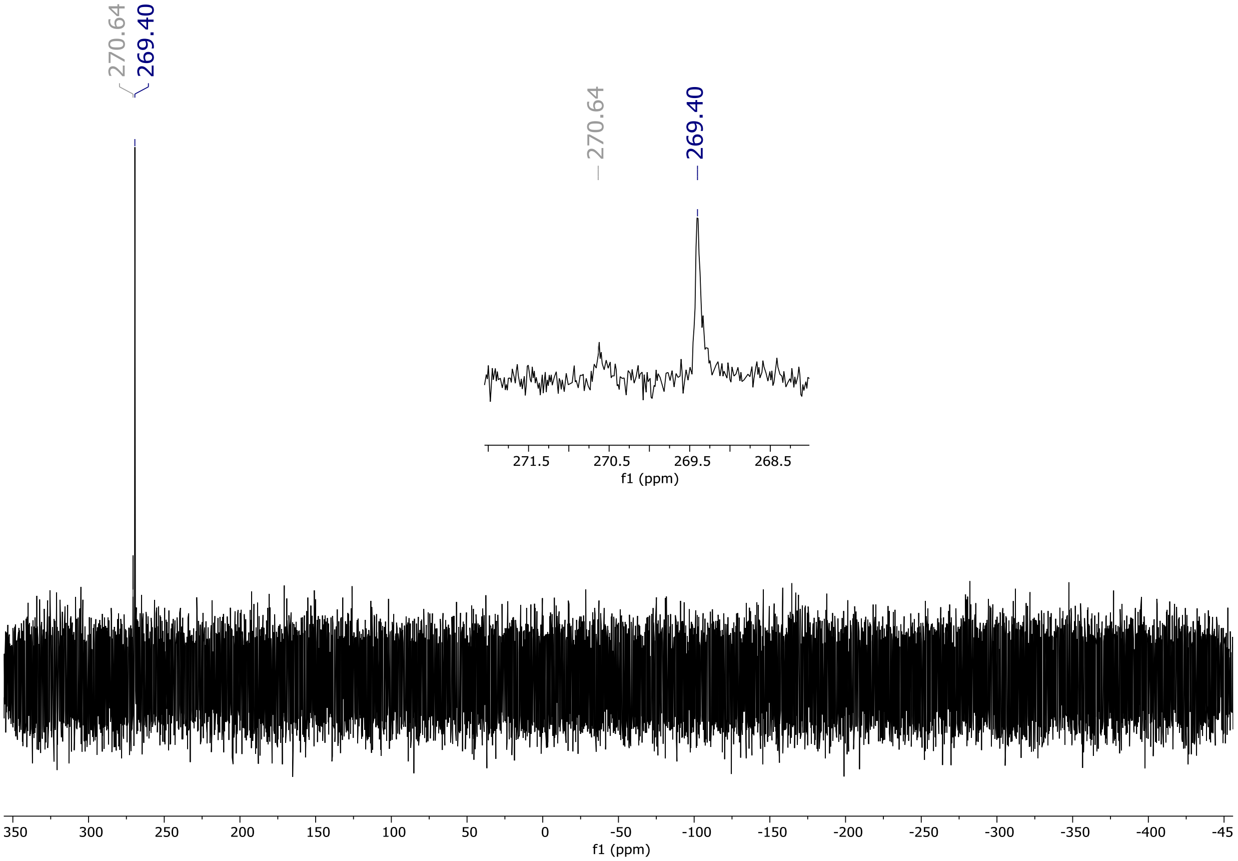

Supplementary Figure 8: ^{31}P NMR spectrum of **2** (C_6D_6). Little **5** has already formed (grey).

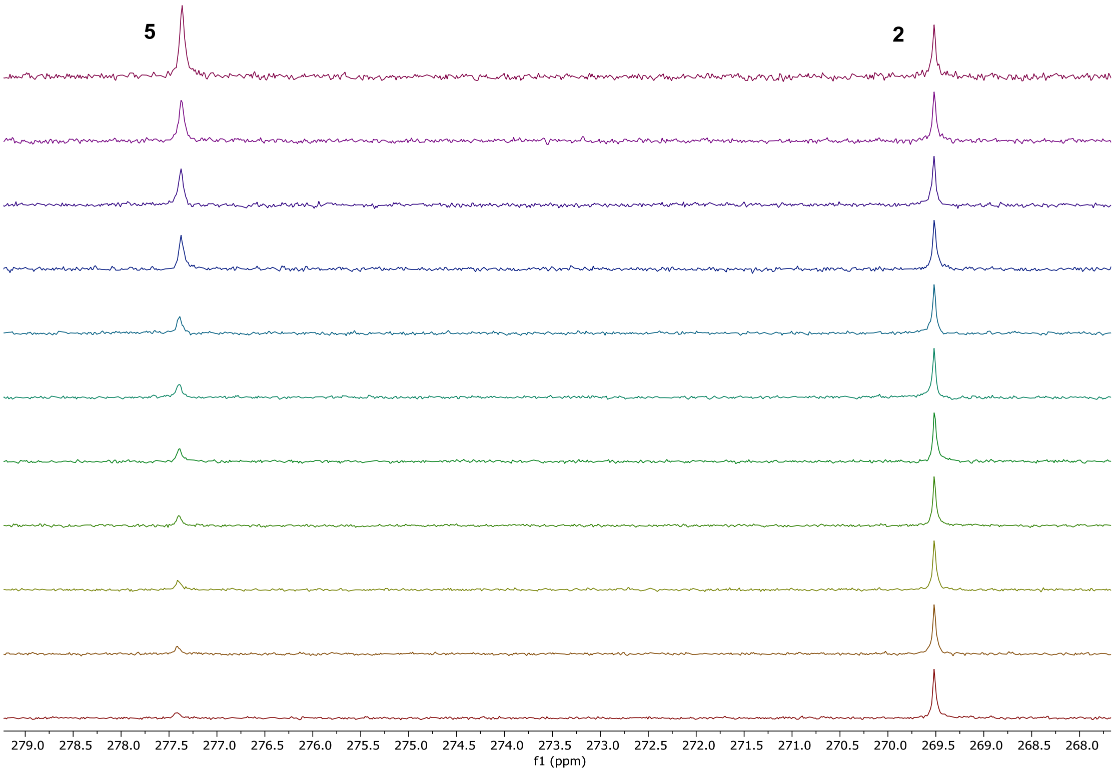

Supplementary Figure 9: Conversion of **2** to **5** monitored at 70°C *via* ^{31}P NMR.

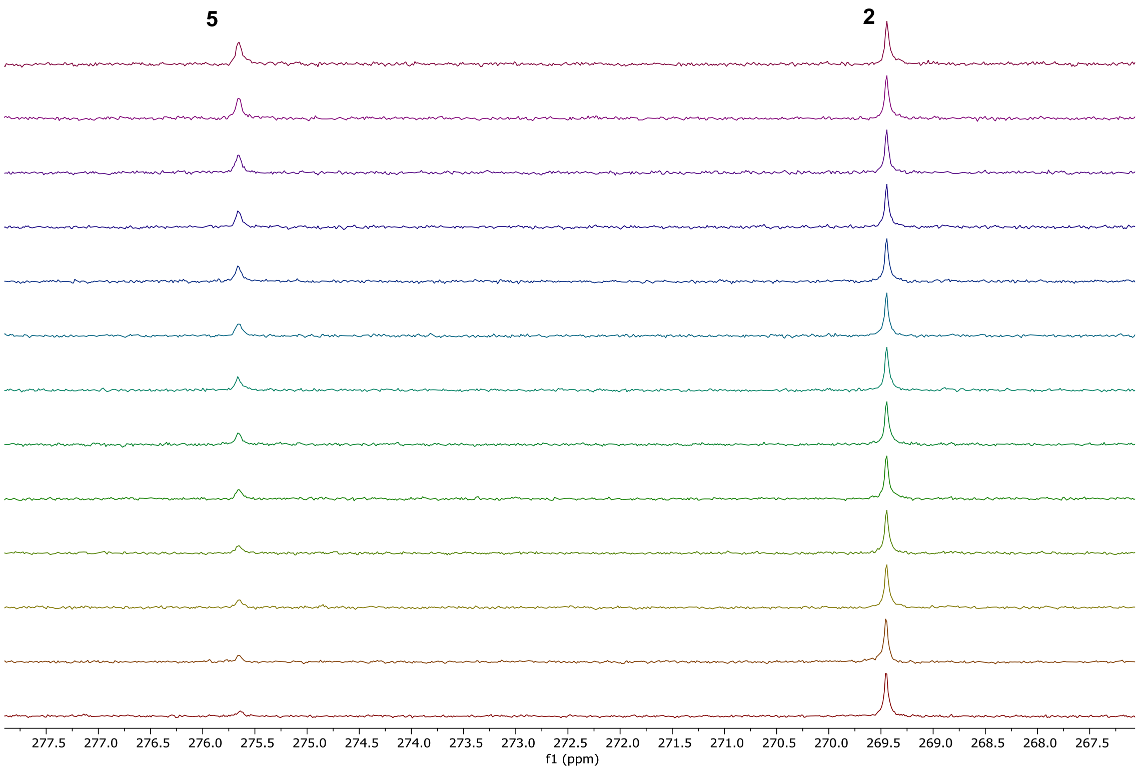

Supplementary Figure 10: Conversion of **2** to **5** monitored at 60°C *via* ^{31}P NMR.

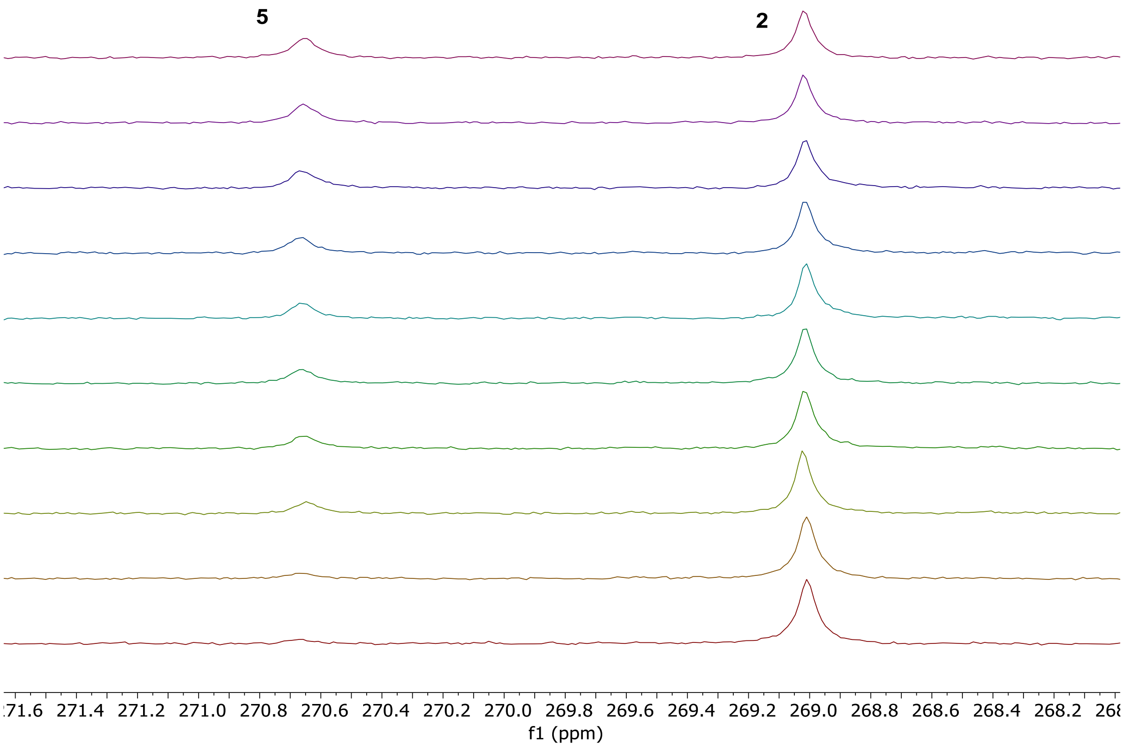

Supplementary Figure 11: Conversion of **2** to **5** monitored at 18°C *via* ^{31}P NMR.

Supplementary Table 1: Conversions of **2** to **5** at 70°C. **2** (30 mg) was dissolved in toluene-d8 and placed in a VT-NMR machine preheated to 70°C. ^{31}P NMR spectra were recorded consecutively in the beginning and in timed intervals later on. The small amount of **5** present at the beginning is included in the conversion values since a correction would not influence the rate determination.

70 °C	
time [min]	conv. []
**5**	**0,1318**
**7**	**0,1558**
**9**	**0,2079**
**11**	**0,2123**
**13**	**0,2273**
**15**	**0,2890**
**19**	**0,3175**
**34**	**0,4587**
**39**	**0,4695**
**44**	**0,5255**
**61**	**0,6389**

Supplementary Table 2: Conversions of **2** to **5** determined at 60°C. **2** (30 mg) was dissolved in toluene-d8 and placed in a VT-NMR machine preheated to 60°C. ^{31}P NMR spectra were recorded in timed intervals. The small amount of **5** present at the beginning is included in the conversion values since a correction would not influence the rate determination.

60 °C	
time [min]	conv. []
**5**	**0,1232**
**13**	**0,1621**
**23**	**0,1812**
**33**	**0,1942**
**43**	**0,2292**
**53**	**0,2417**
**63**	**0,2778**
**73**	**0,2994**
**83**	**0,3012**
**93**	**0,3463**
**103**	**0,3507**
**113**	**0,3903**
**123**	**0,4117**

Supplementary Table 3: Conversions of **2** to **5** determined at 18°C. **2** (30 mg) was dissolved in toluene-d8 and placed in an NMR machine. ^{31}P NMR spectra were recorded in timed intervals. The small amount of **5** present at the beginning is included in the conversion values since a correction would not influence the rate determination.

**18°C**	
time [min]	conv. []
**1440**	**0,0654**
**2880**	**0,0909**
**8640**	**0,1667**
**10080**	**0,1935**
**11520**	**0,2188**
**12960**	**0,2308**
**14400**	**0,2481**
**18720**	**0,2908**
**20160**	**0,3056**
**21600**	**0,3289**

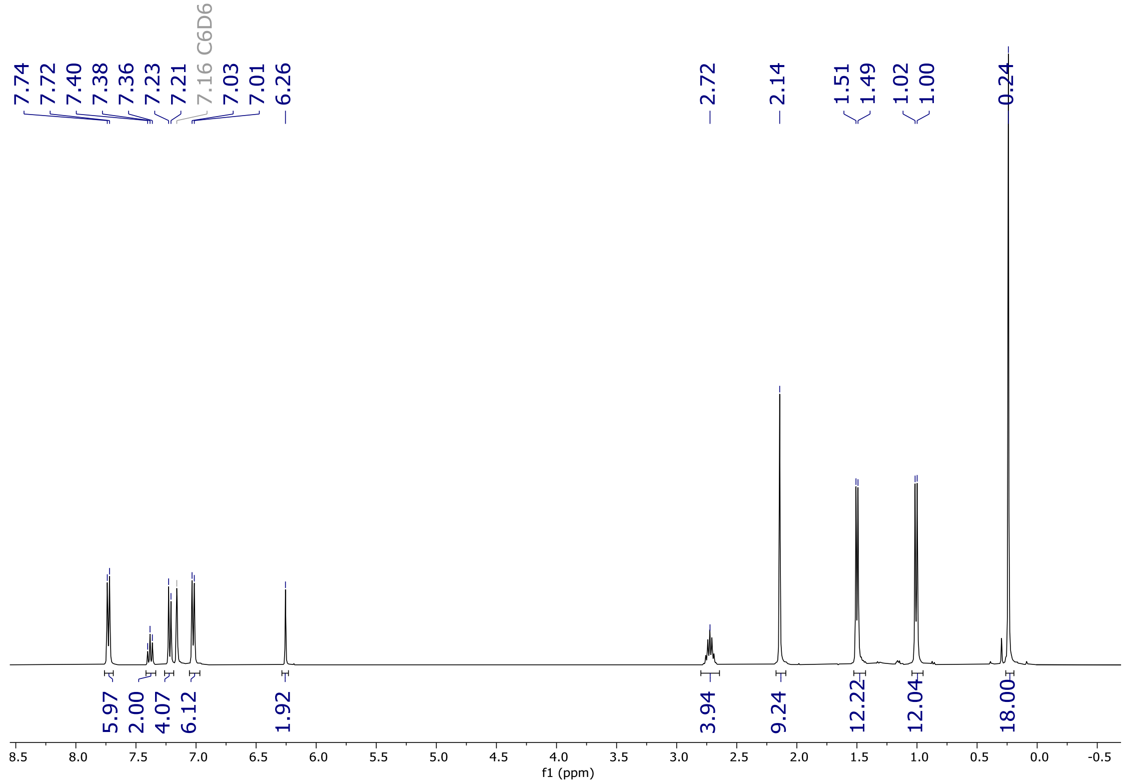

Supplementary Figure 12: ^{1}H NMR of CO_2 activation product **3** (C_6D_6).

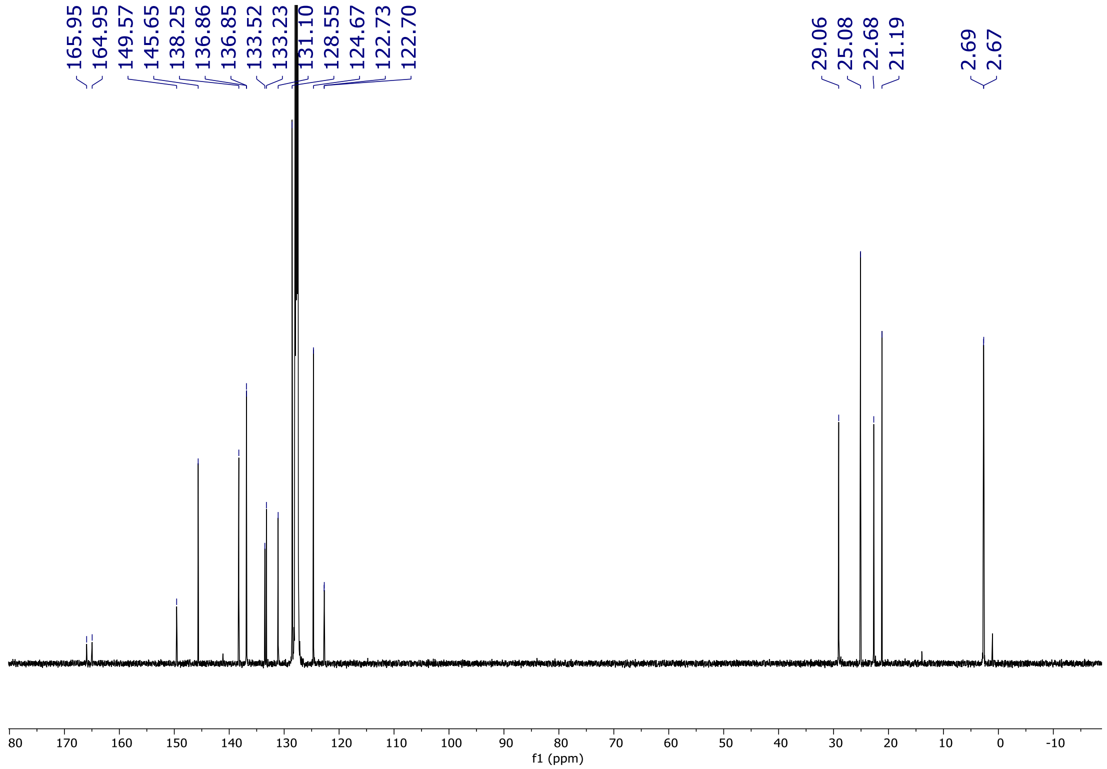

Supplementary Figure 13: ^{13}C NMR spectrum of CO_2 activation product **3** (C_6D_6).

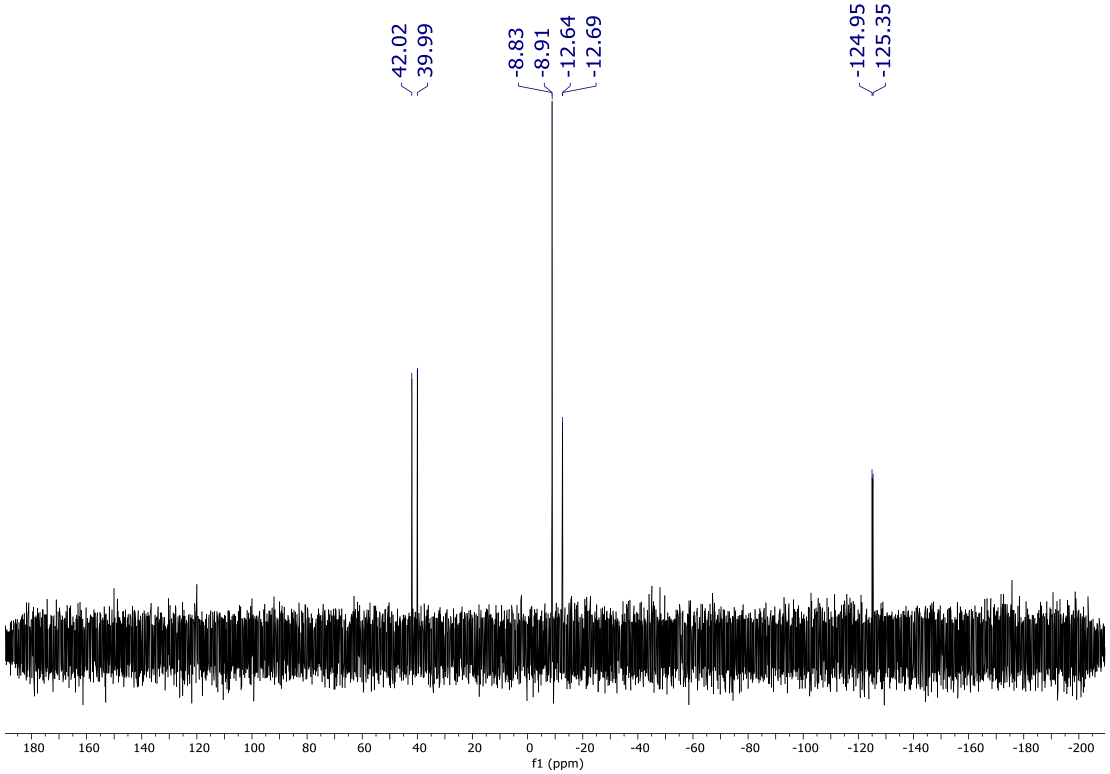

Supplementary Figure 14: ^{29}Si NMR spectrum of CO_2 activation product **3** (C_6D_6).

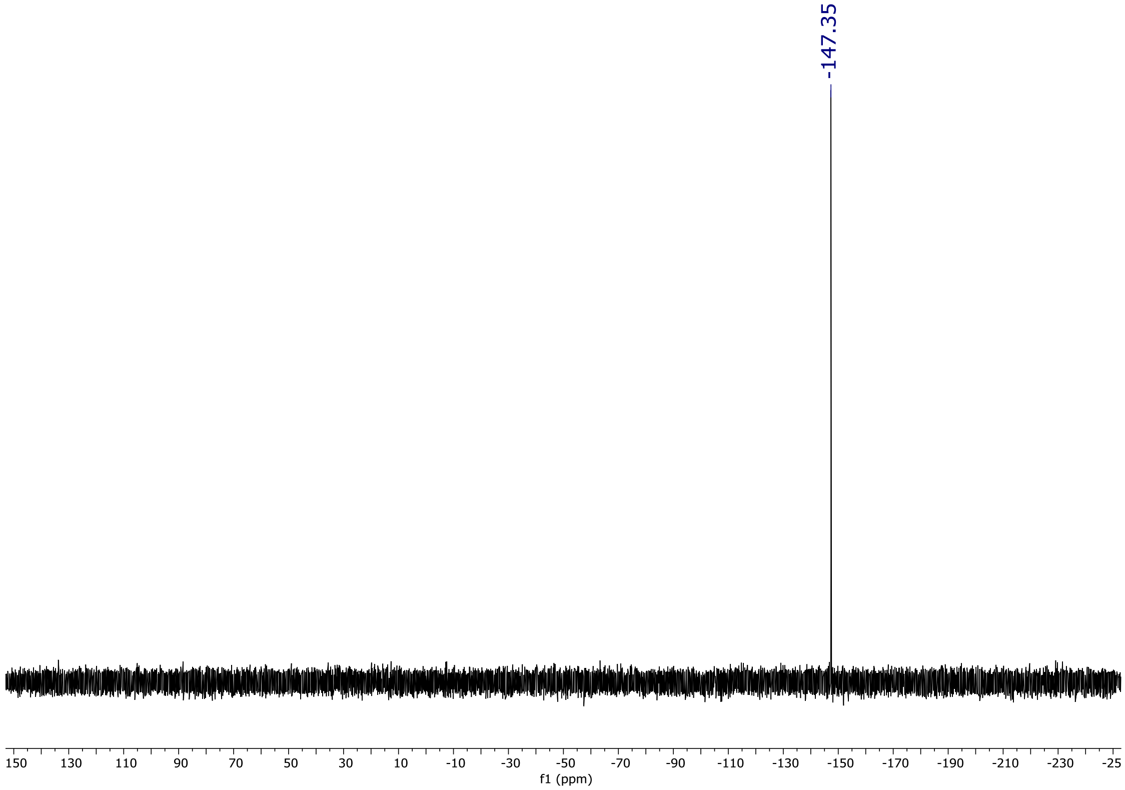

Supplementary Figure 15: $^{31}P\{^1H\}$ NMR spectrum of CO_2 activation product **3** (C_6D_6).

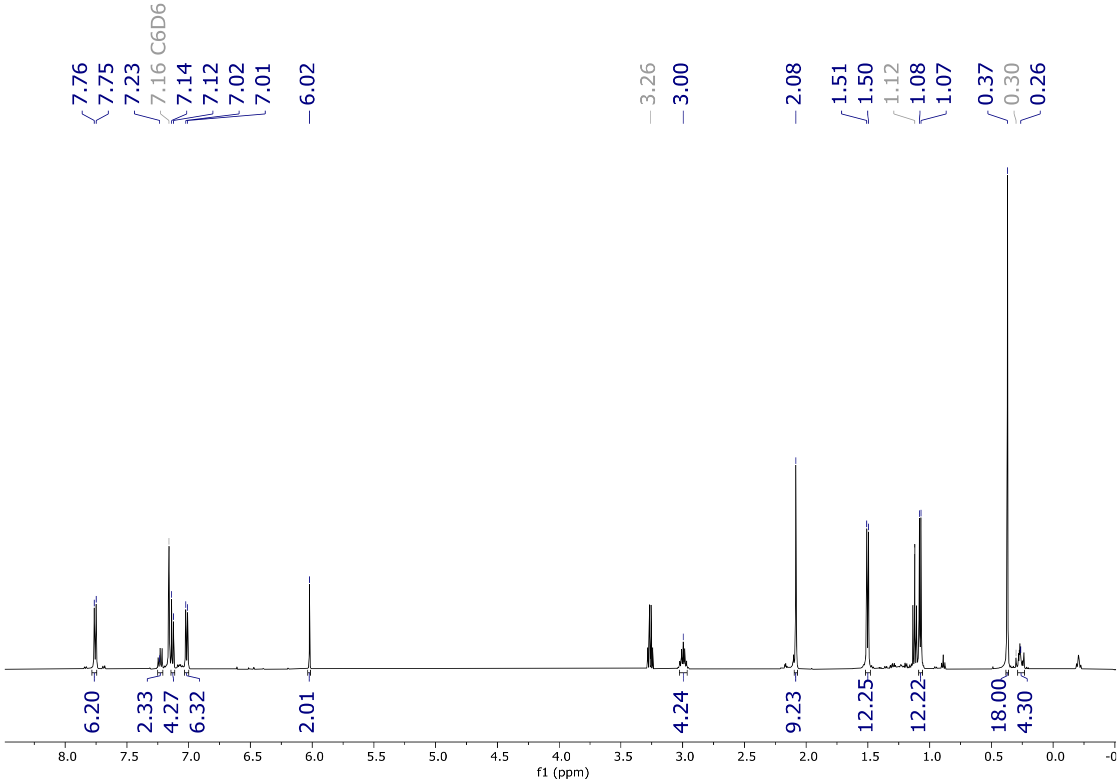

Supplementary Figure 16: ^{1}H NMR of compound **4** (C_6D_6) including co-crystallized Et_2O.

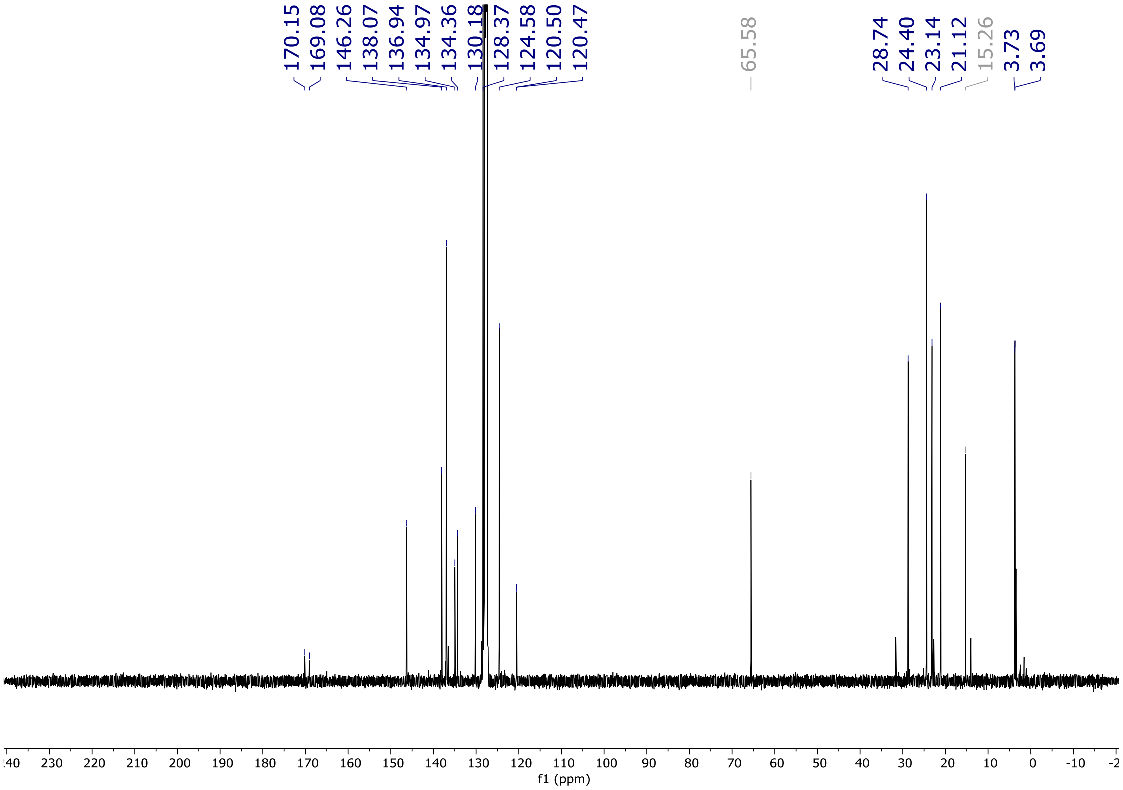

Supplementary Figure 17: ^{13}C NMR of ethylene activation product **4** (C_6D_6) including co-crystallized Et_2O.

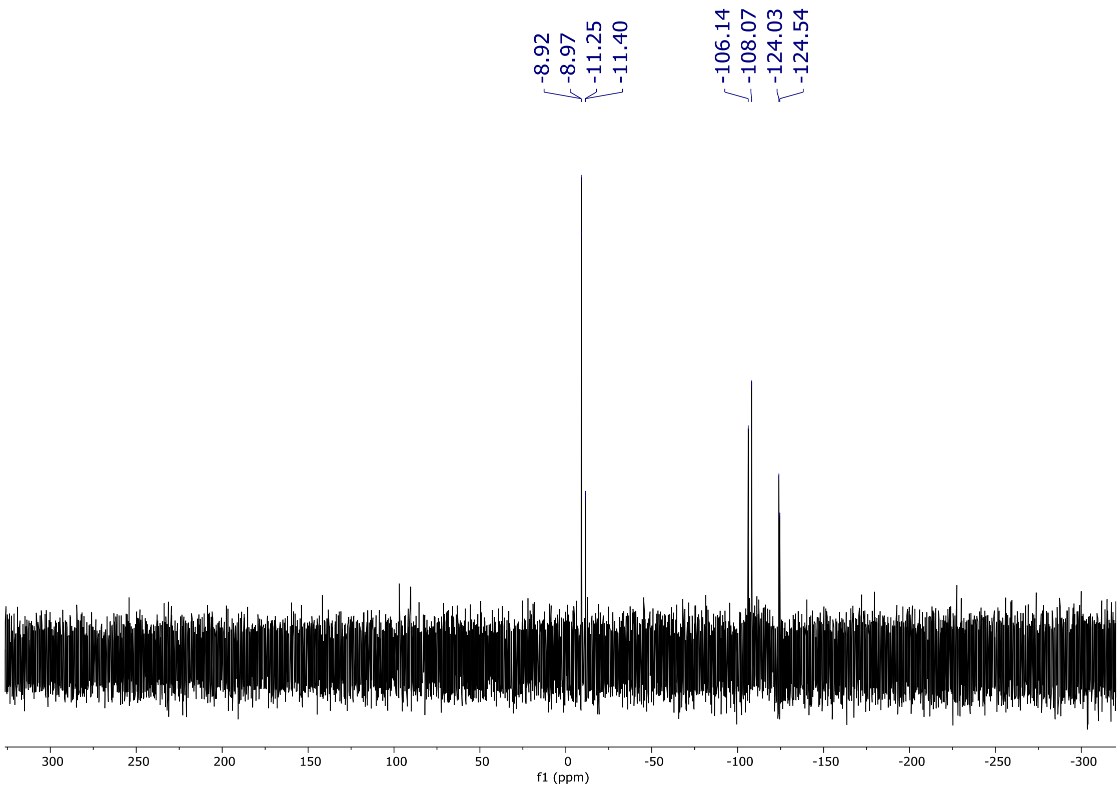

Supplementary Figure 18: ^{29}Si NMR of compound **4** (C_6D_6) including co-crystallized Et_2O.

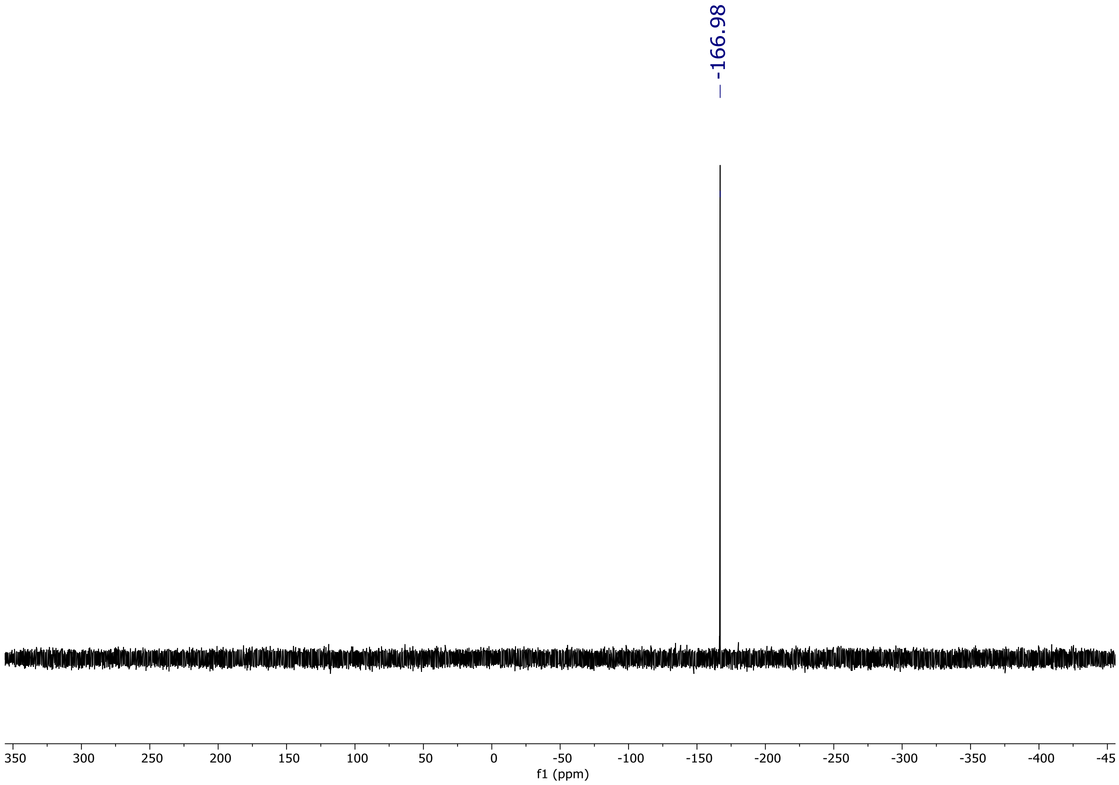

Supplementary Figure 19: ^{31}P NMR of ethylene activation product **4** (C_6D_6) including co-crystallized Et_2O.

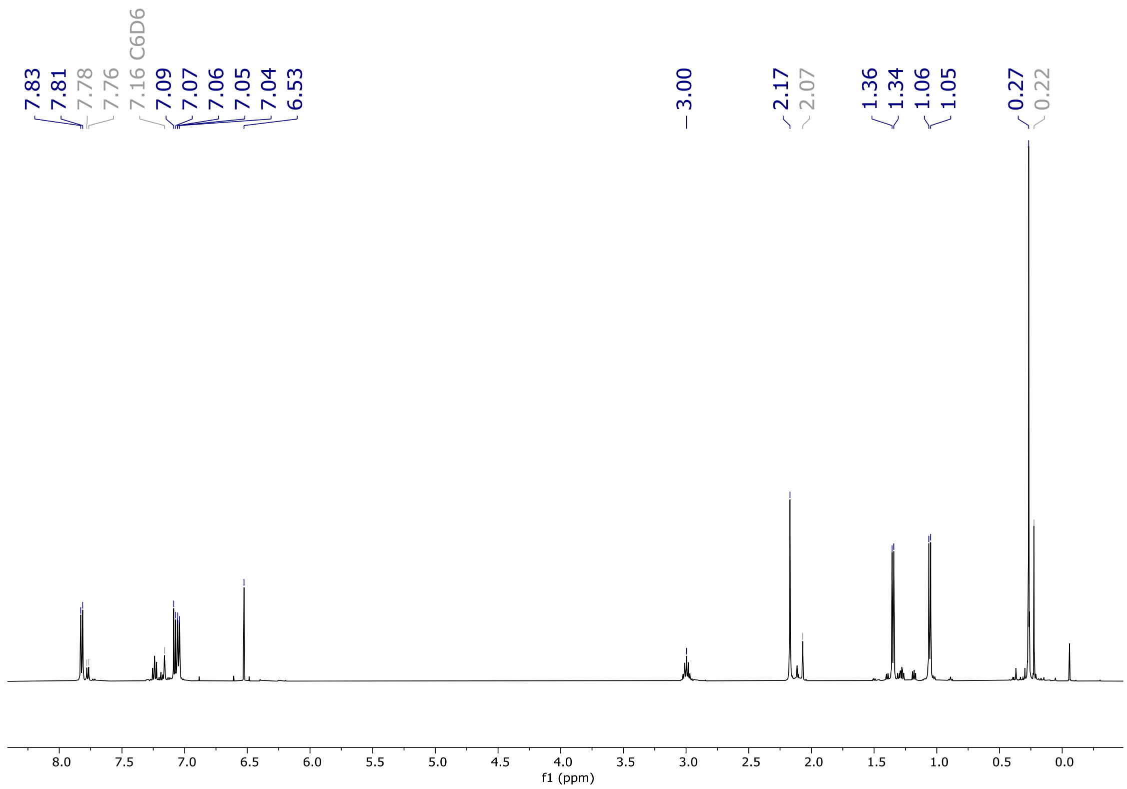

Supplementary Figure 20: ^{1}H NMR spectrum of **5** (C_6D_6).

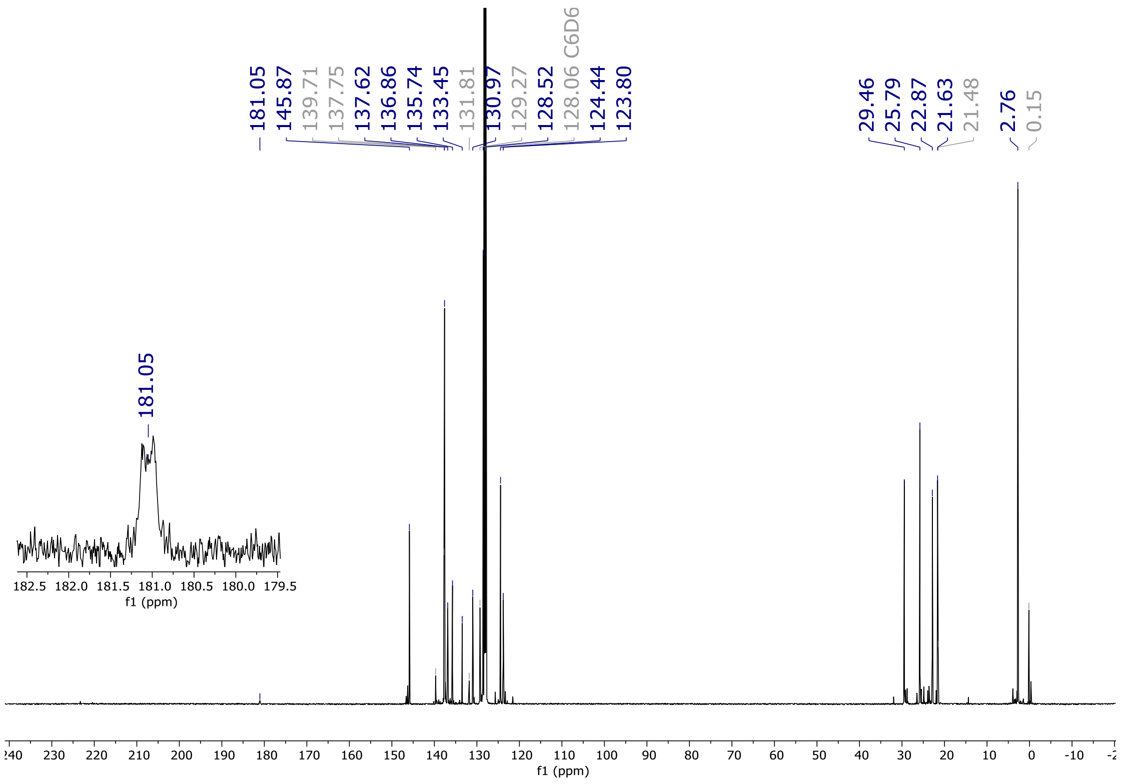

Supplementary Figure 21: ^{13}C NMR spectrum of **5** (C_6D_6).

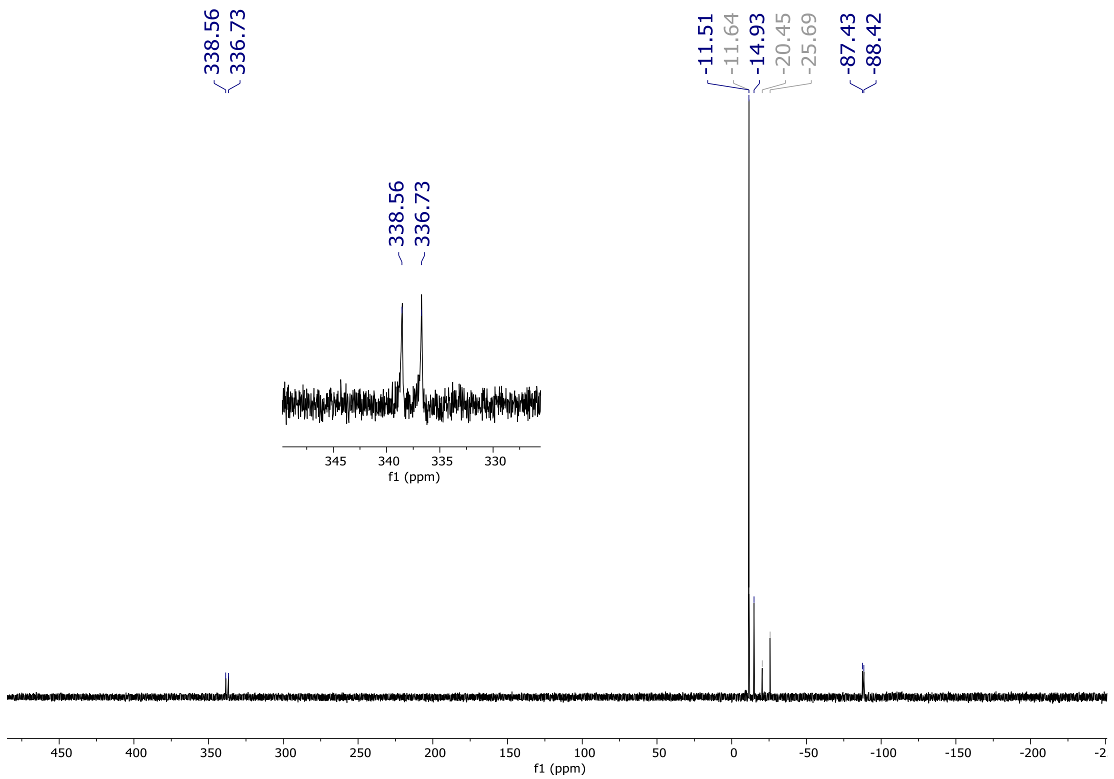

Supplementary Figure 22: ^{29}Si NMR spectrum of **5** (C_6D_6).

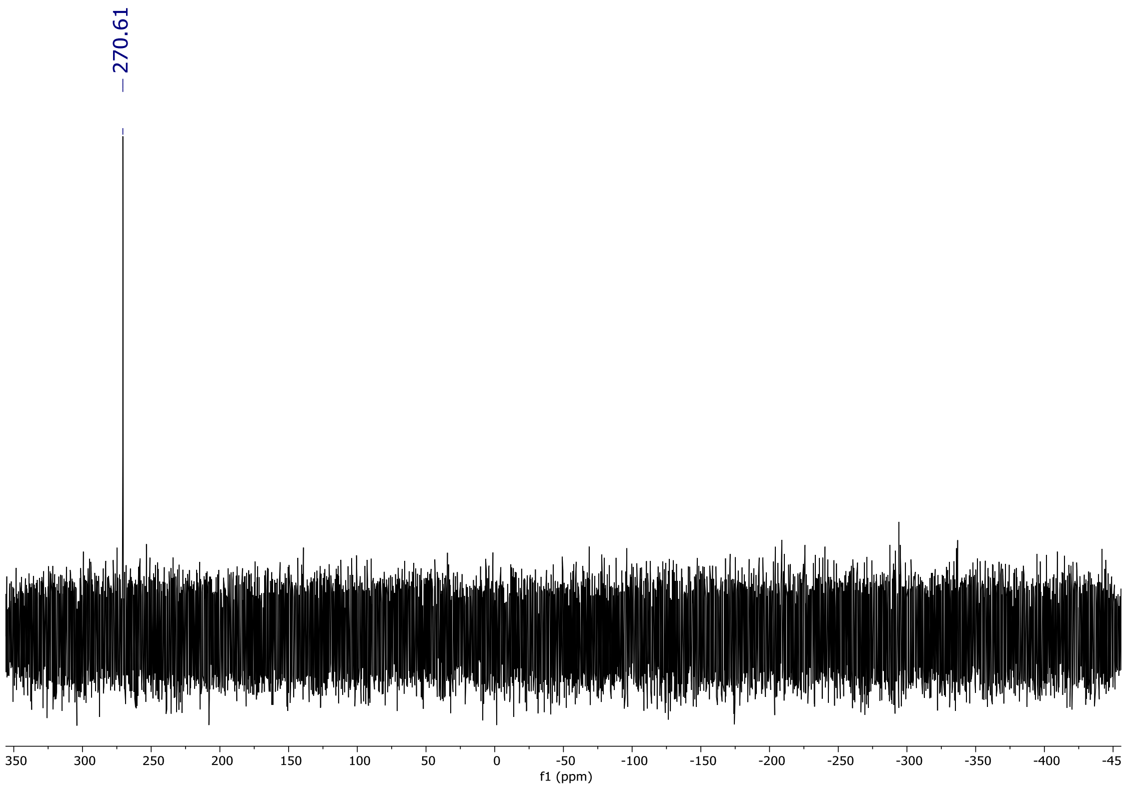

Supplementary Figure 23: ^{31}P NMR spectrum of **5** (C_6D_6).

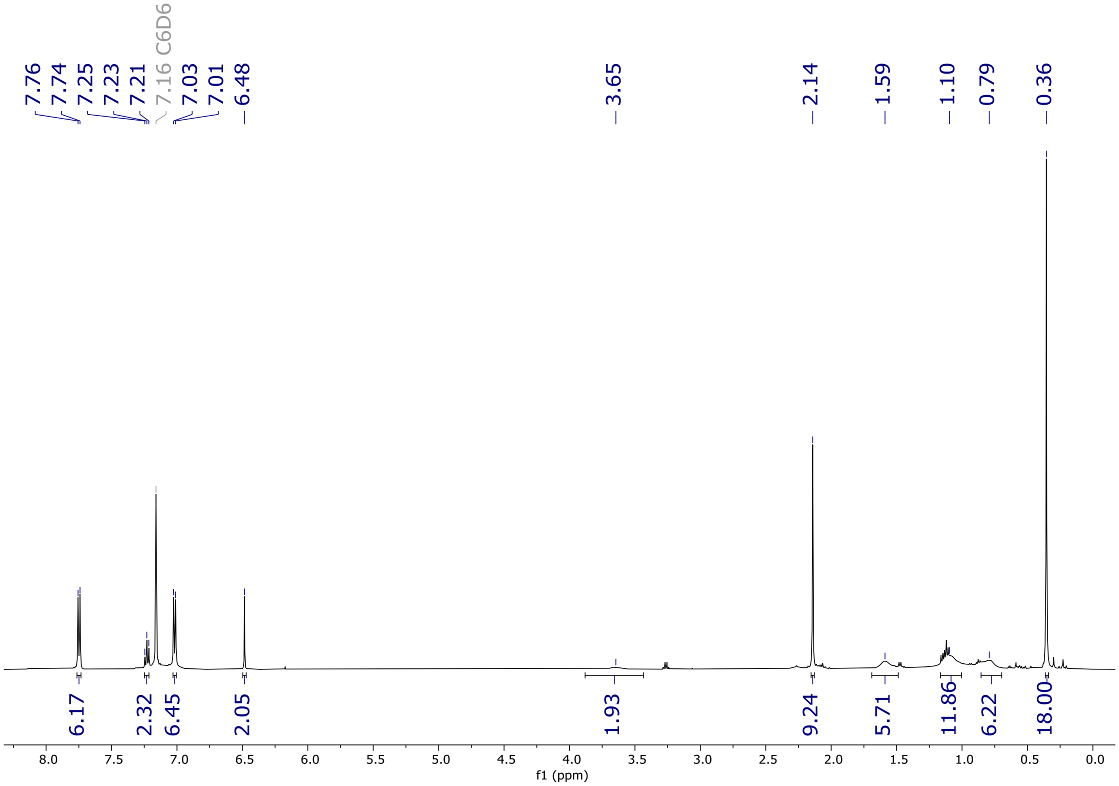

Supplementary Figure 24: ^{1}H NMR spectrum of **6** (C_6D_6).

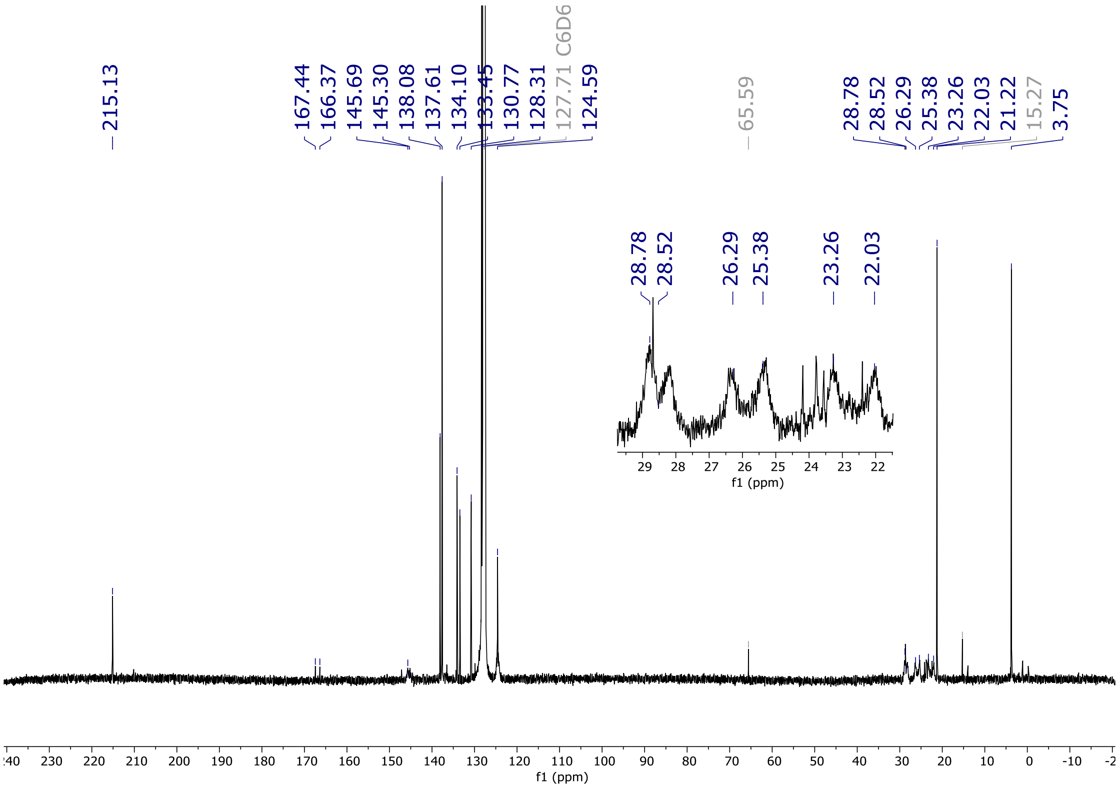

Supplementary Figure 25: ^{13}C NMR spectrum of **6** (C_6D_6).

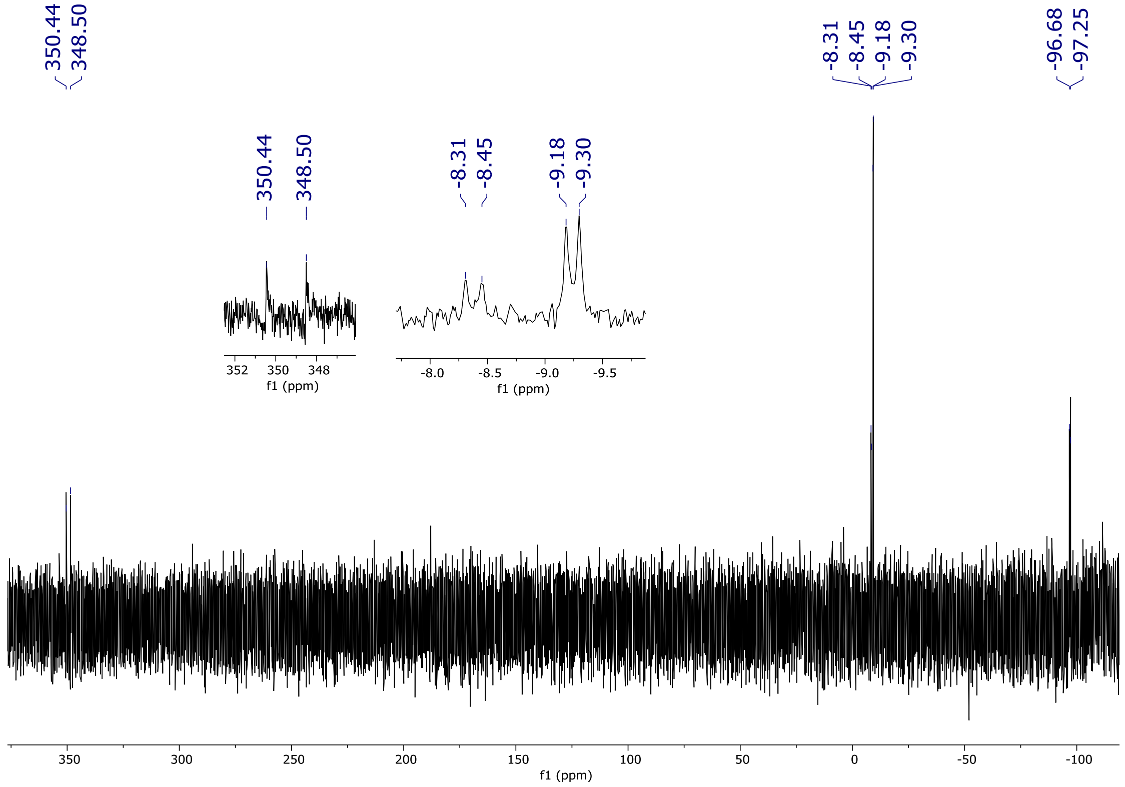

Supplementary Figure 26: ^{29}Si NMR spectrum of **6** (C_6D_6).

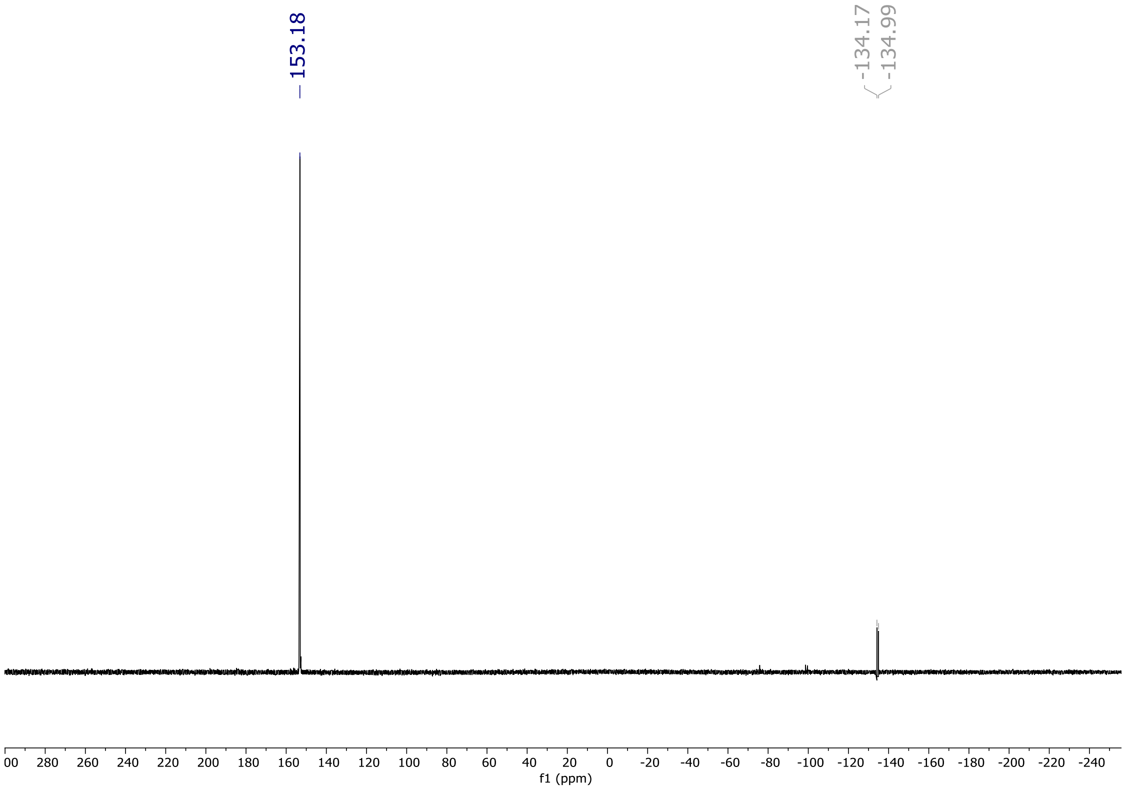

Supplementary Figure 27: ^{31}P NMR spectrum of **6** (C_6D_6) The visible impurity is IDippPH.

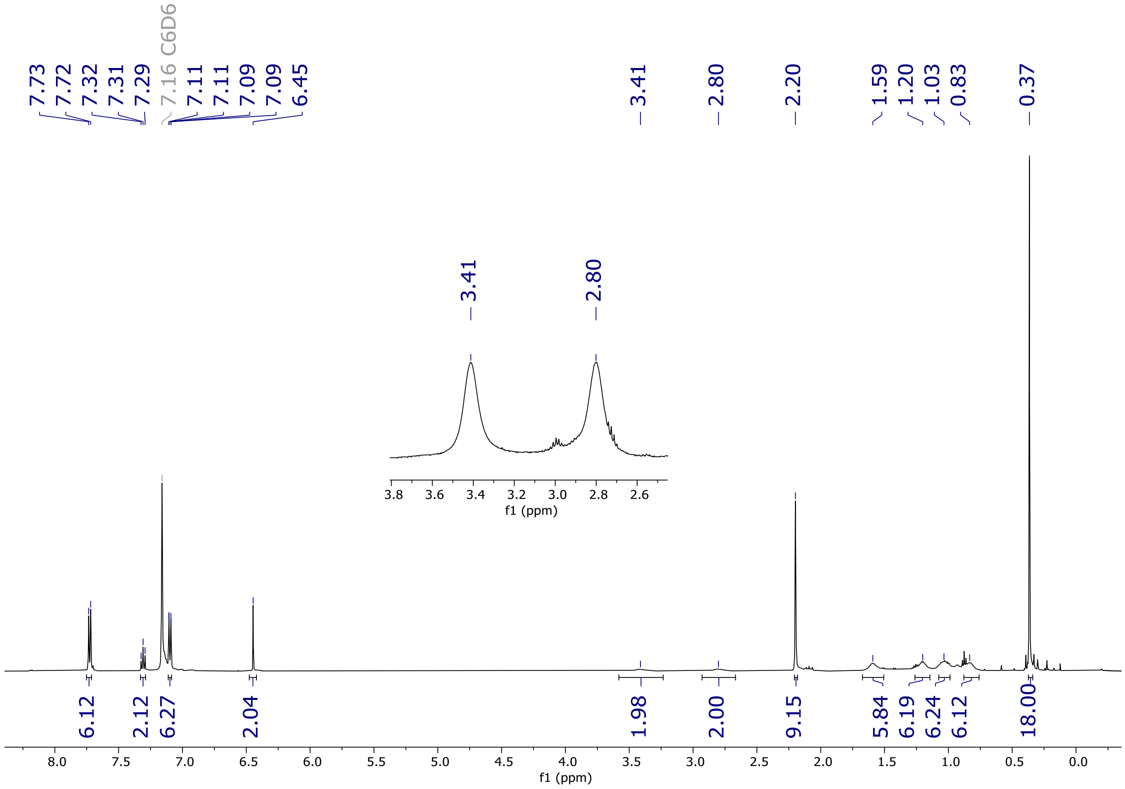

Supplementary Figure 28: ^{1}H NMR spectrum of **7** (C_6D_6).

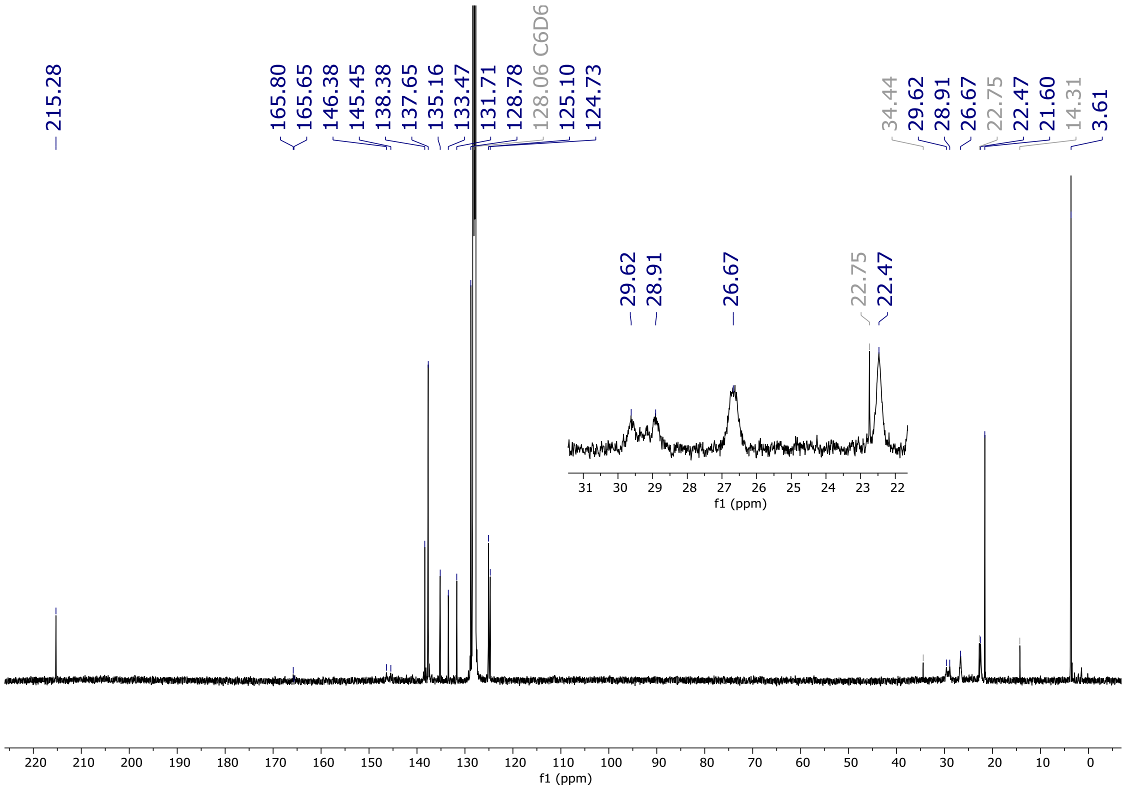

Supplementary Figure 29: ^{13}C NMR spectrum of **7** (C_6D_6).

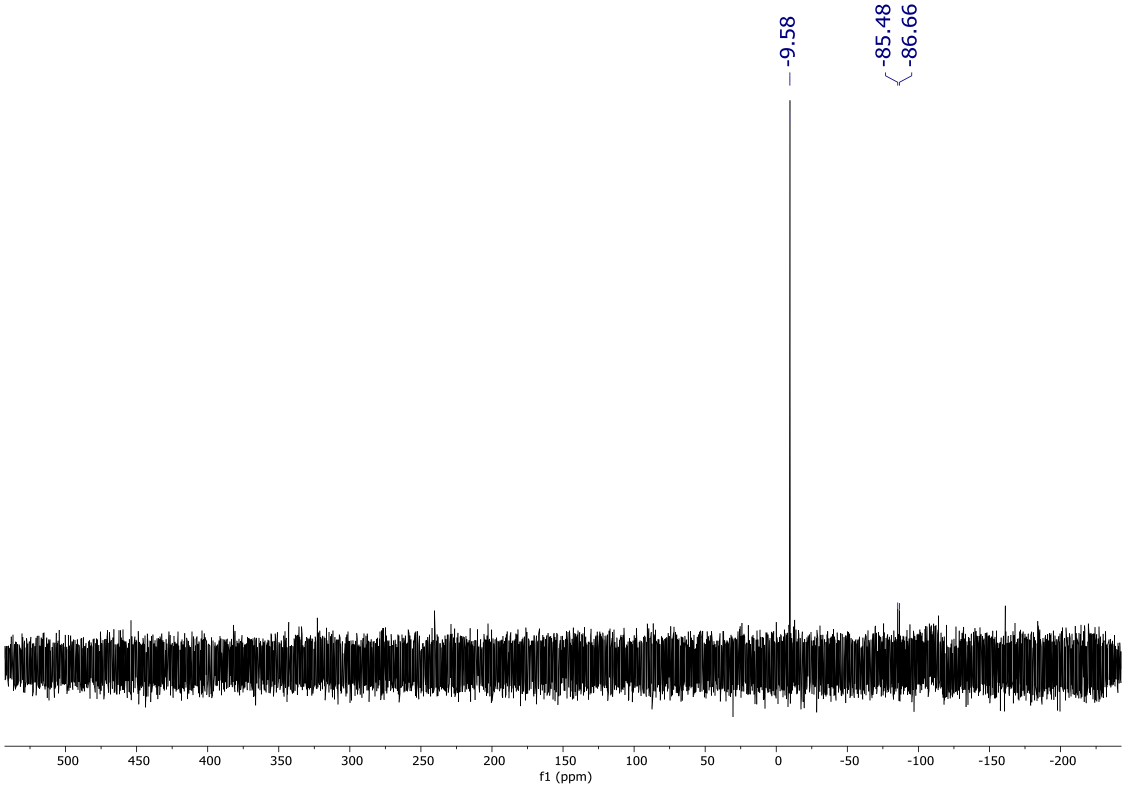

Supplementary Figure 30: ^{29}Si NMR spectrum of **7** (C_6D_6).

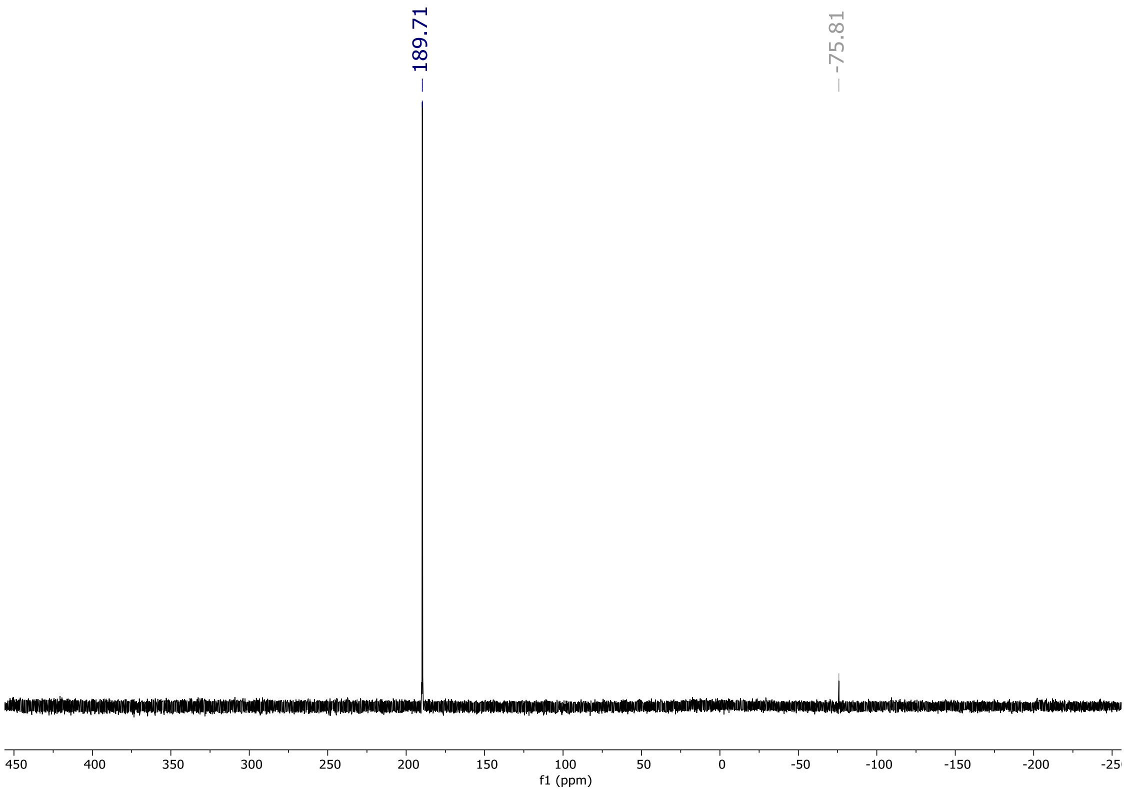

Supplementary Figure 31: ^{31}P NMR spectrum of **7** (C_6D_6).

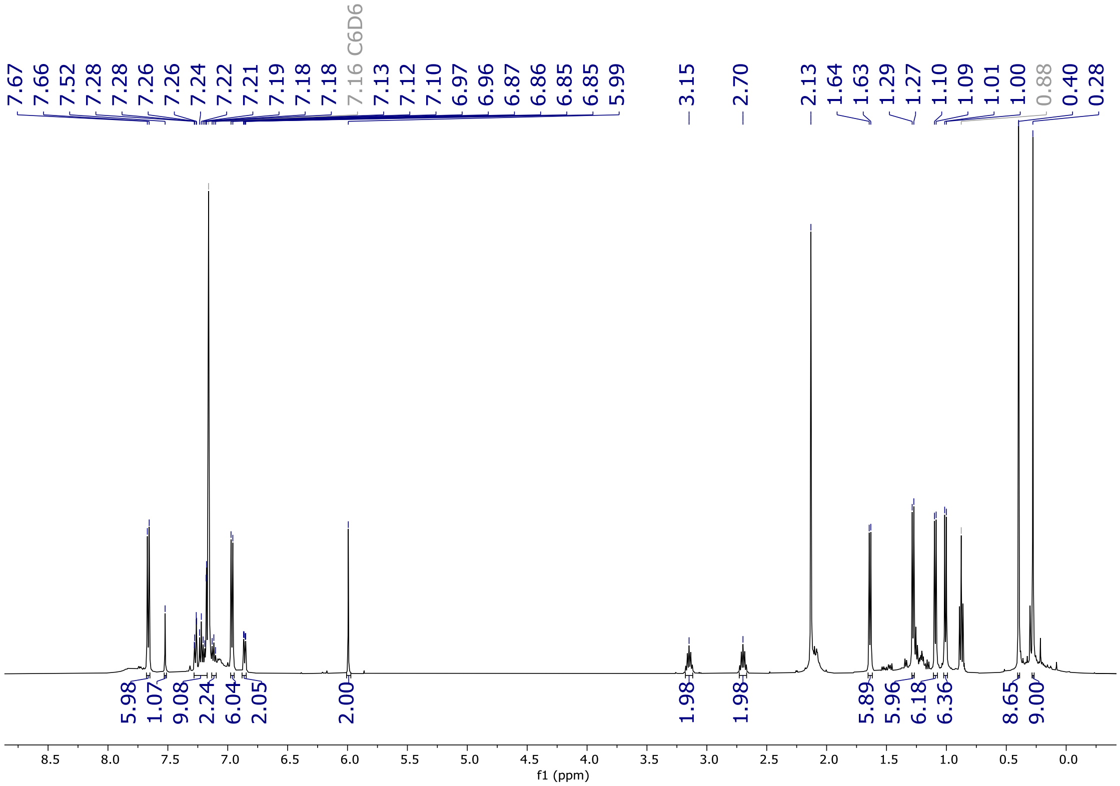

Supplementary Figure 32: ^{1}H NMR spectrum of **8a** (C_6D_6).

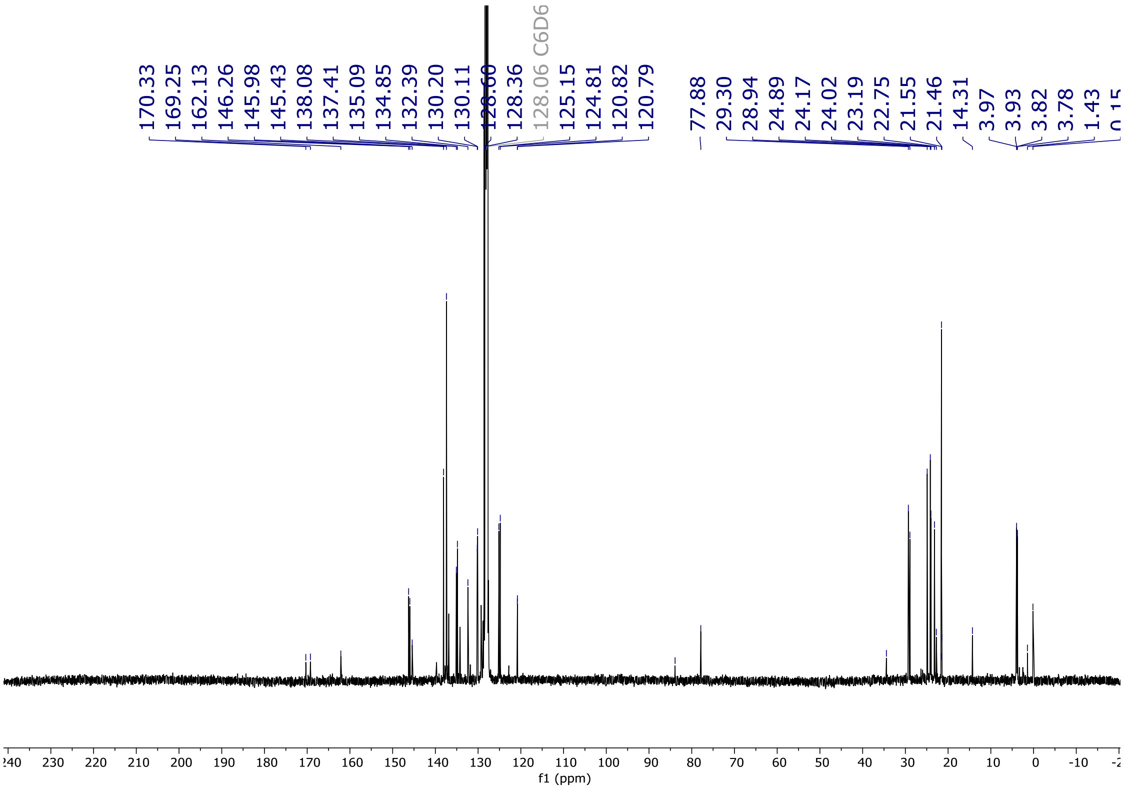

Supplementary Figure 33: ^{13}C NMR spectrum of **8a** (C_6D_6).

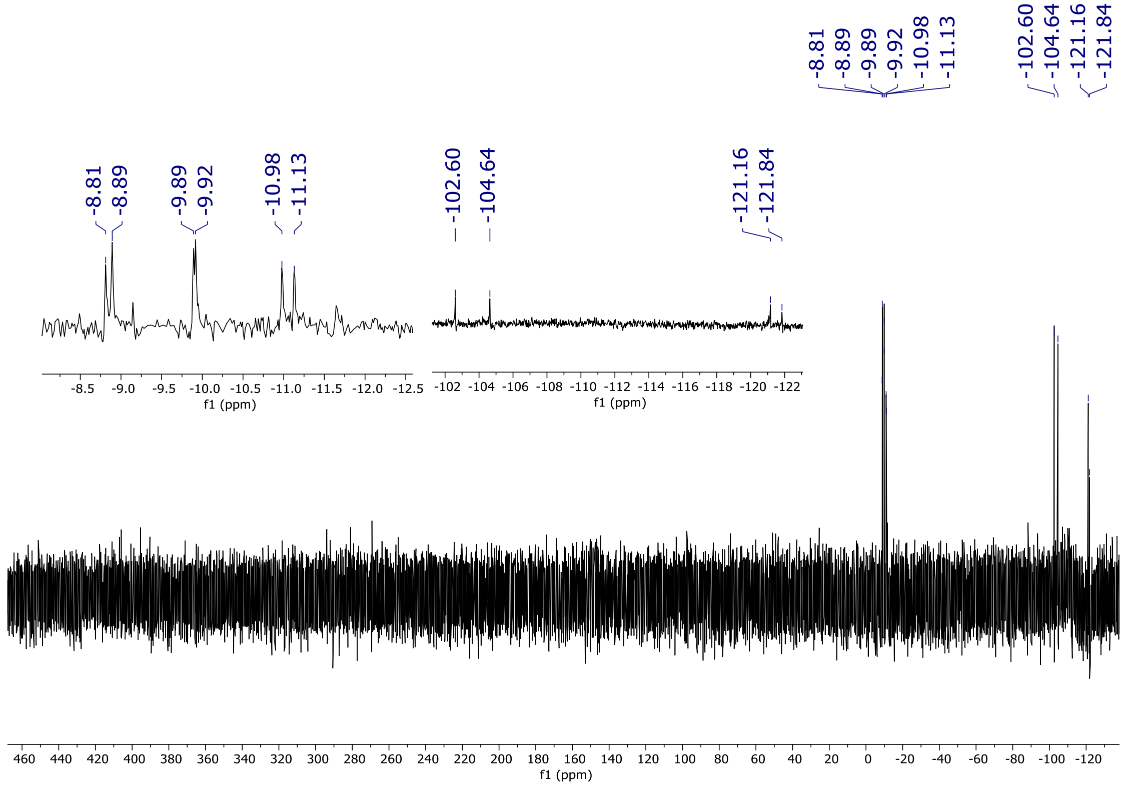

Supplementary Figure 34: ^{29}Si NMR spectrum of **8a** (C_6D_6).

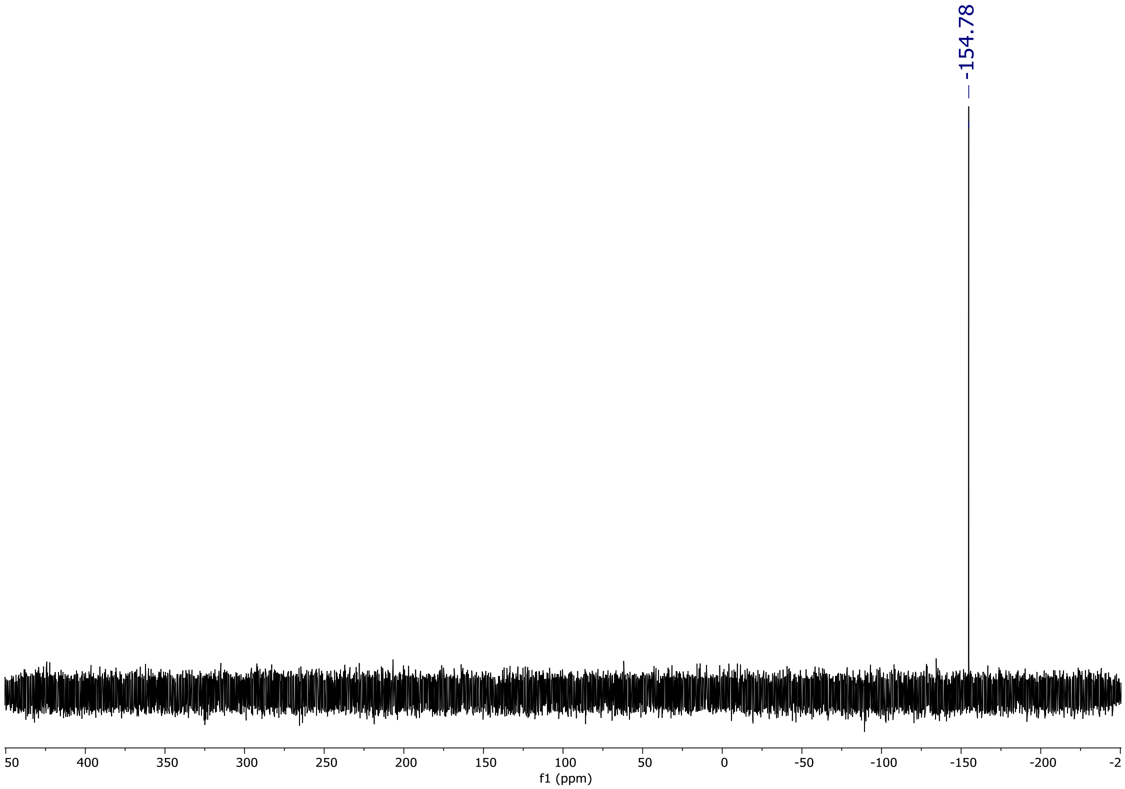

Supplementary Figure 35: ^{31}P NMR spectrum of **8a** (C_6D_6).

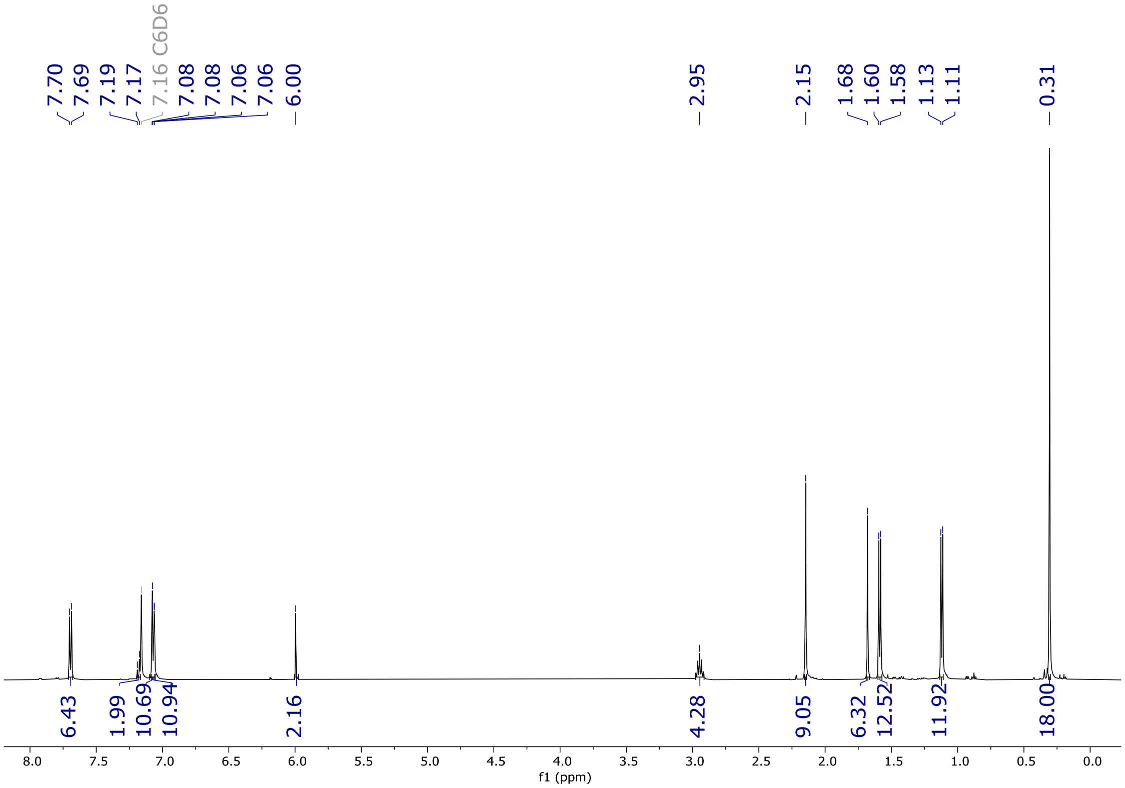

Supplementary Figure 36: ^{1}H NMR spectrum of **8b** (C_6D_6).

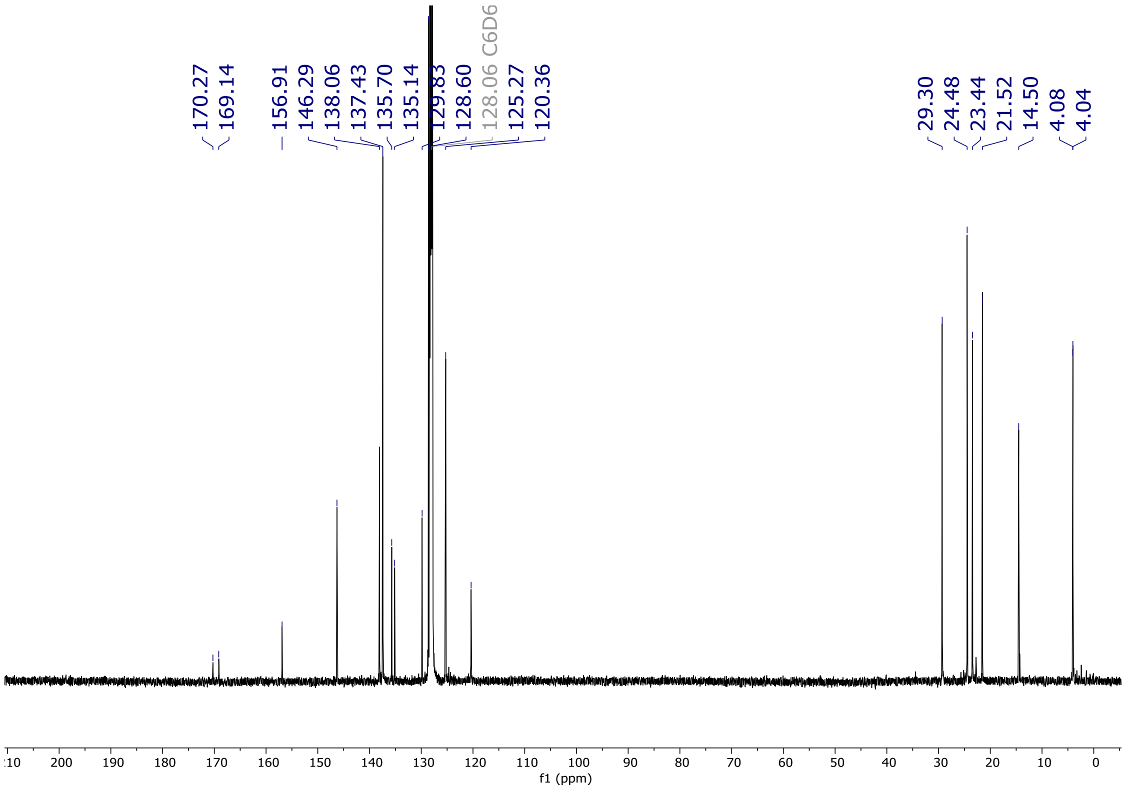

Supplementary Figure 37: ^{13}C NMR spectrum of **8b** (C_6D_6).

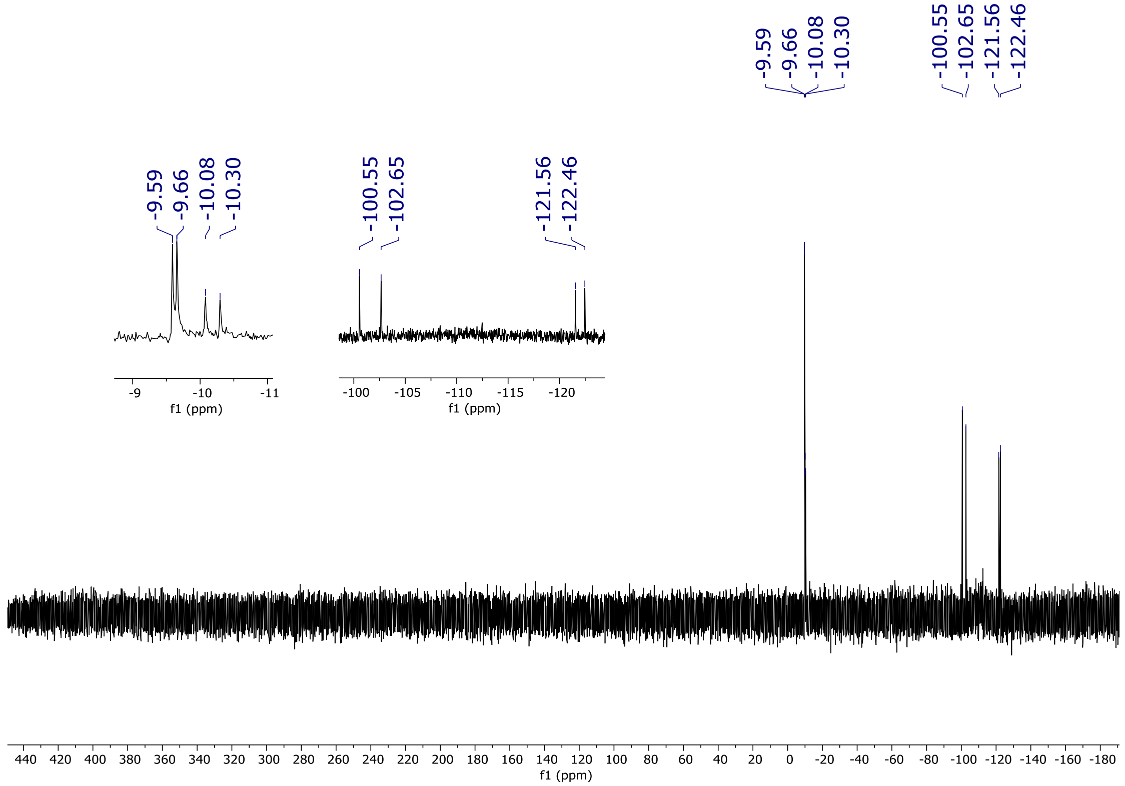

Supplementary Figure 38: ^{29}Si NMR spectrum of **8b** (C_6D_6).

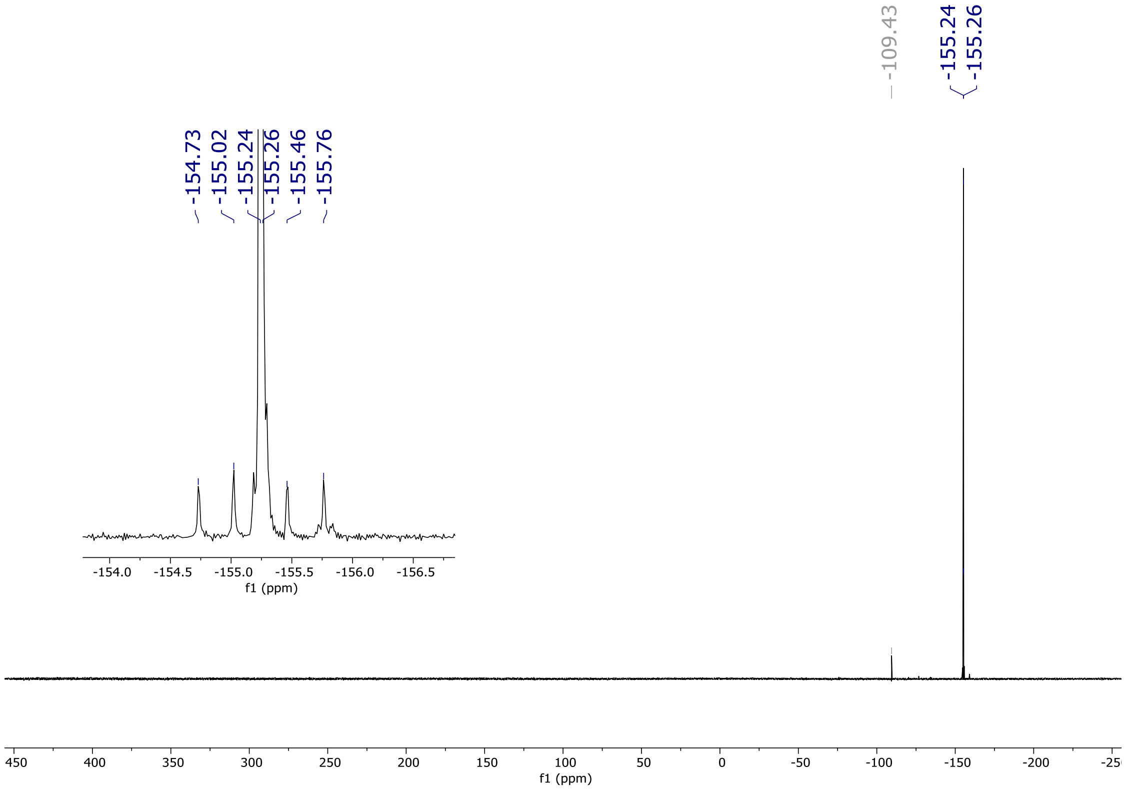

Supplementary Figure 39: ^{31}P NMR spectrum of **8b** (C_6D_6).

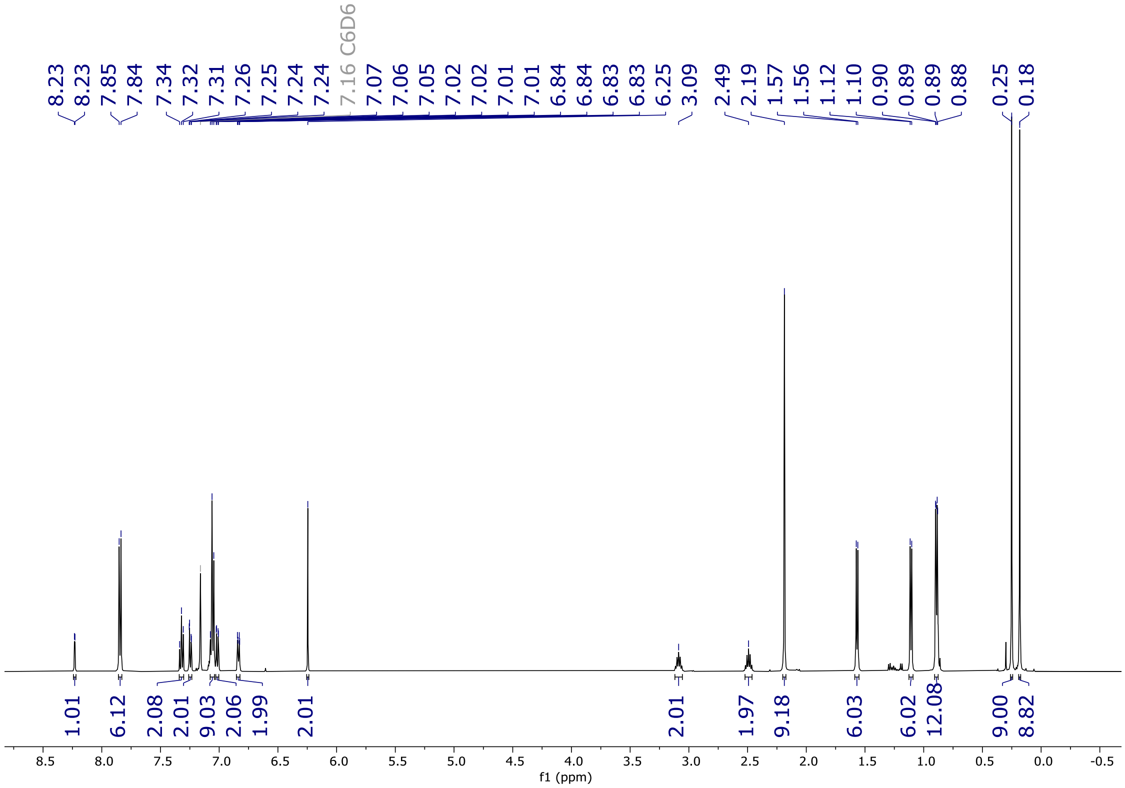

Supplementary Figure 40: ^{1}H NMR spectrum of **9a** (C_6D_6).

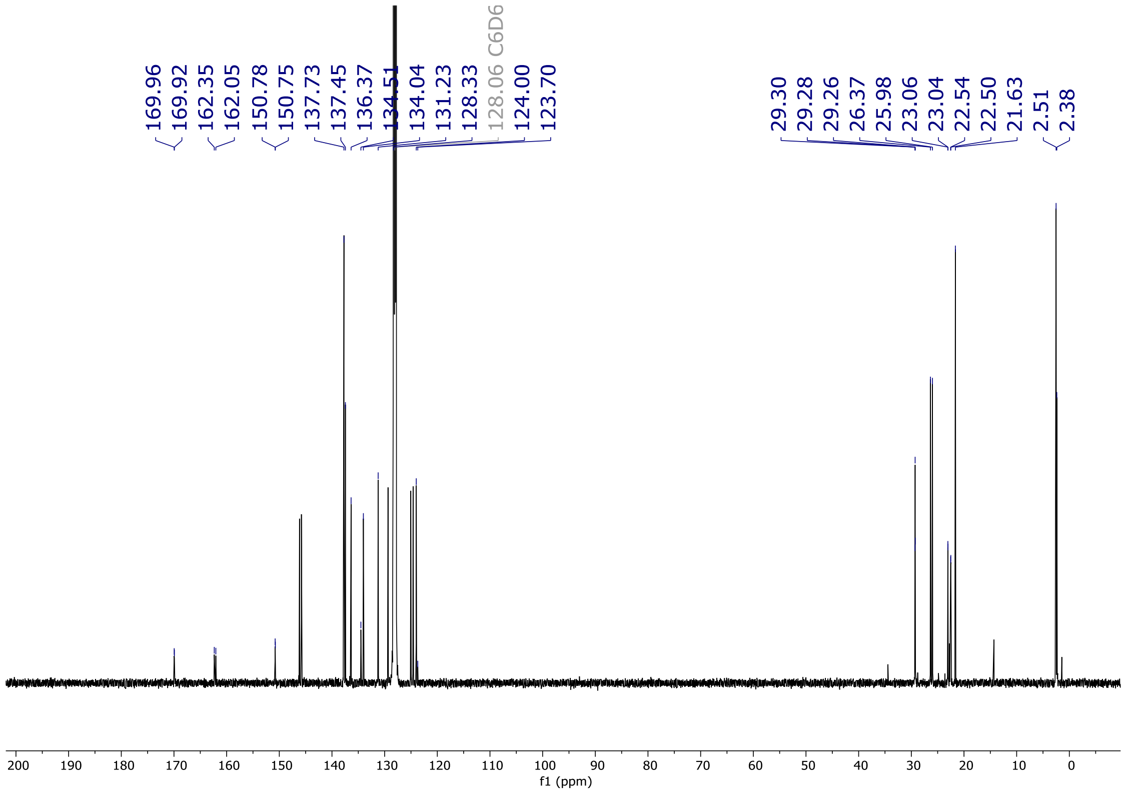

Supplementary Figure 41: ^{13}C NMR spectrum of **9a** (C_6D_6).

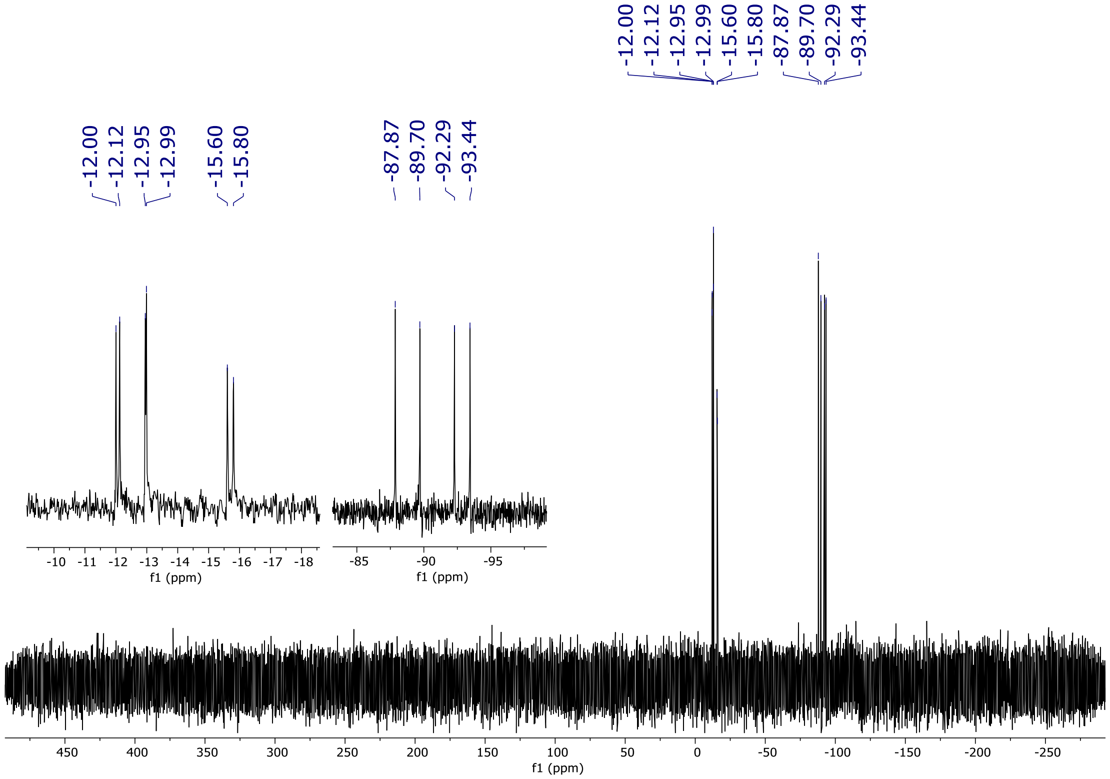

Supplementary Figure 42: ^{29}Si NMR spectrum of **9a** (C_6D_6).

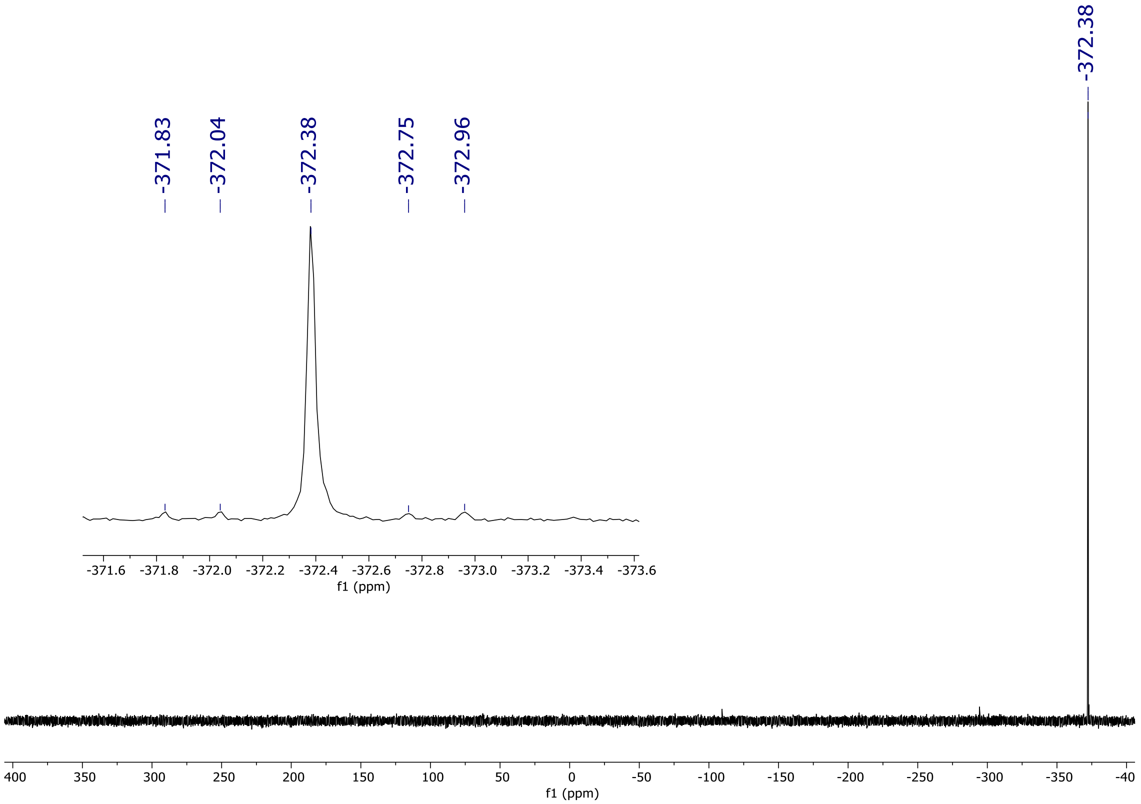

Supplementary Figure 43: ^{31}P NMR spectrum of **9a** (C_6D_6).

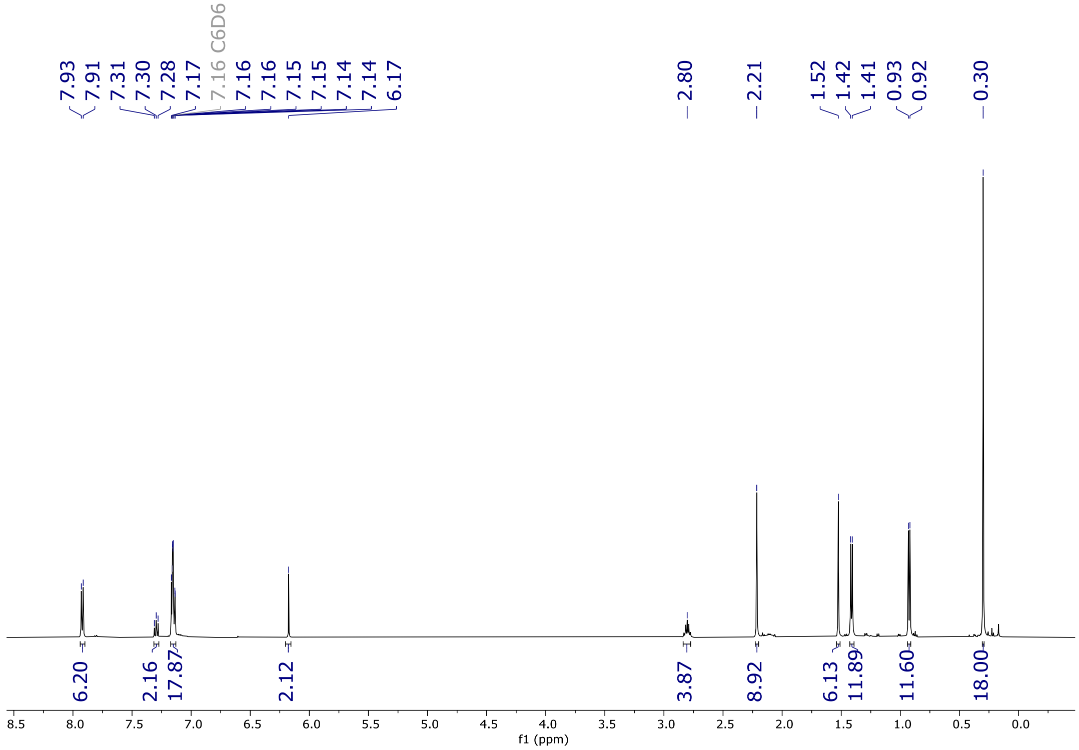

Supplementary Figure 44: ^{1}H NMR spectrum of **9b** (C_6D_6).

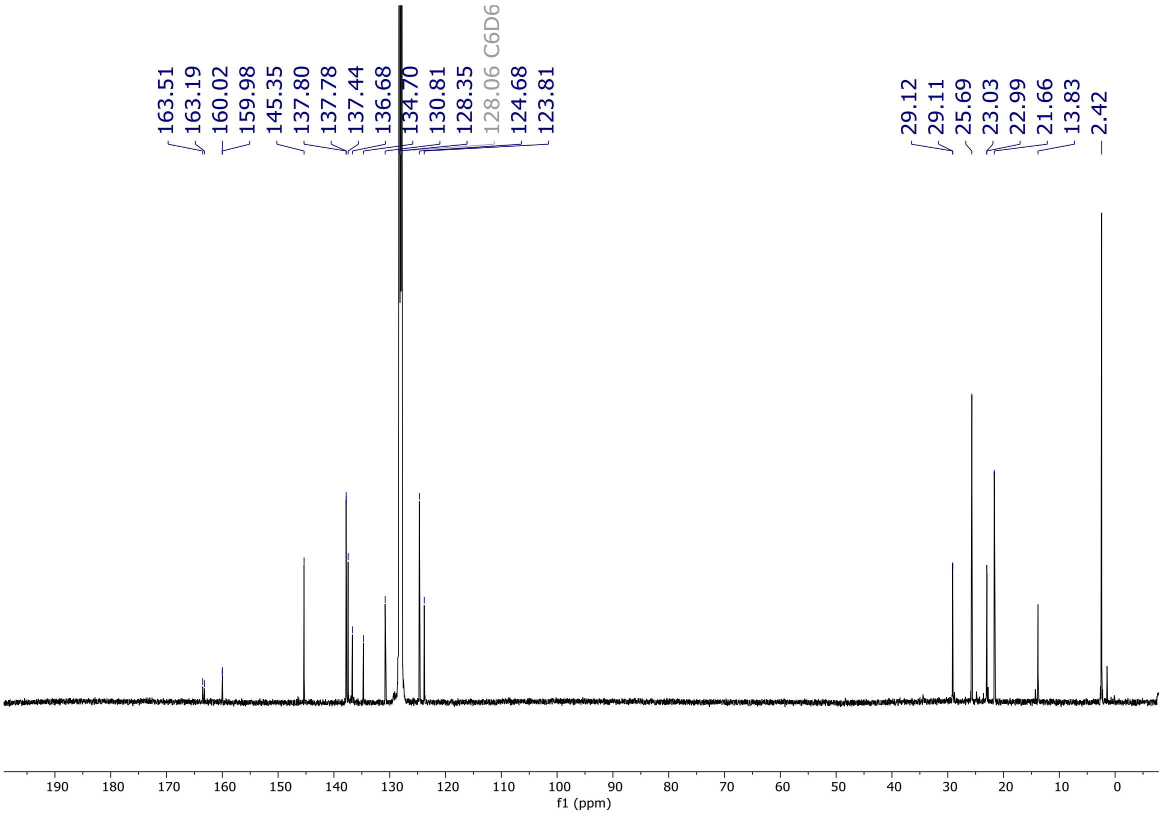

Supplementary Figure 45: ^{13}C NMR spectrum of **9b** (C_6D_6).

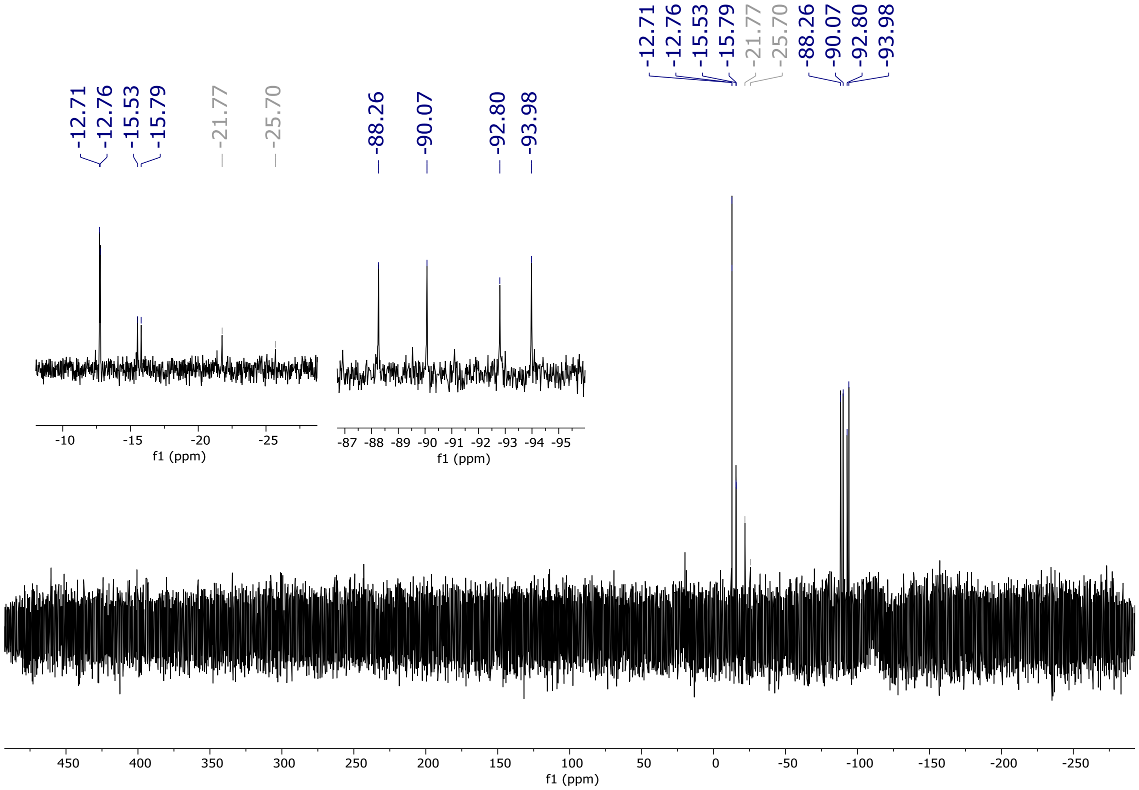

Supplementary Figure 46: ^{29}Si NMR spectrum of **9b** (C_6D_6).

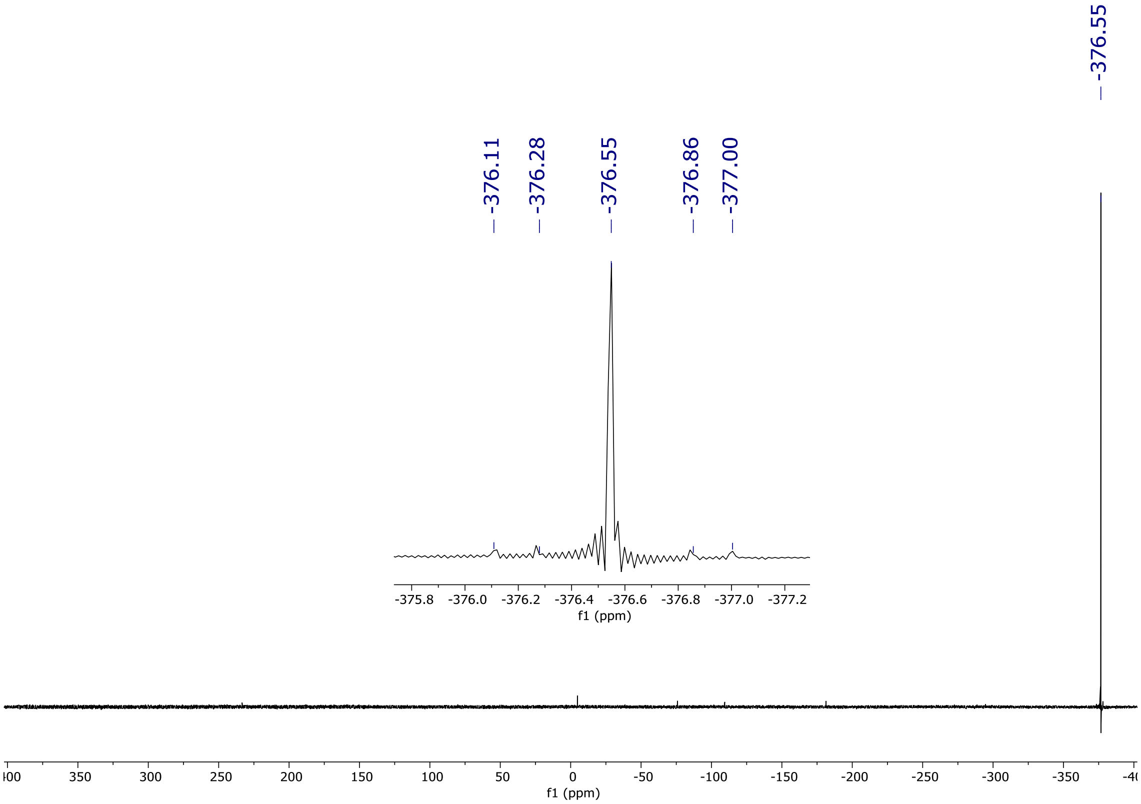

Supplementary Figure 47: ^{31}P NMR spectrum of **9b** (C_6D_6).

LIFDI-MS

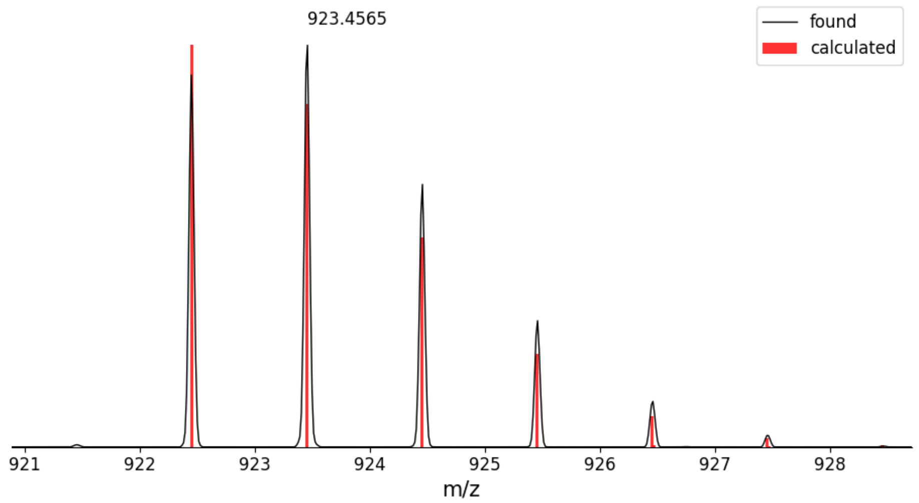

Supplementary Figure 48: LIFDI-MS spectrum for compound **2**.

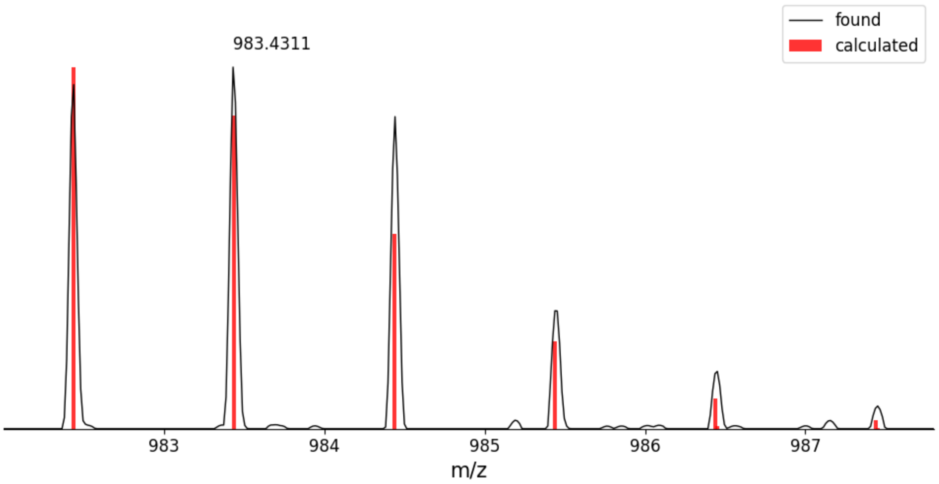

Supplementary Figure 49: LIFDI-MS spectrum for compound **3**.

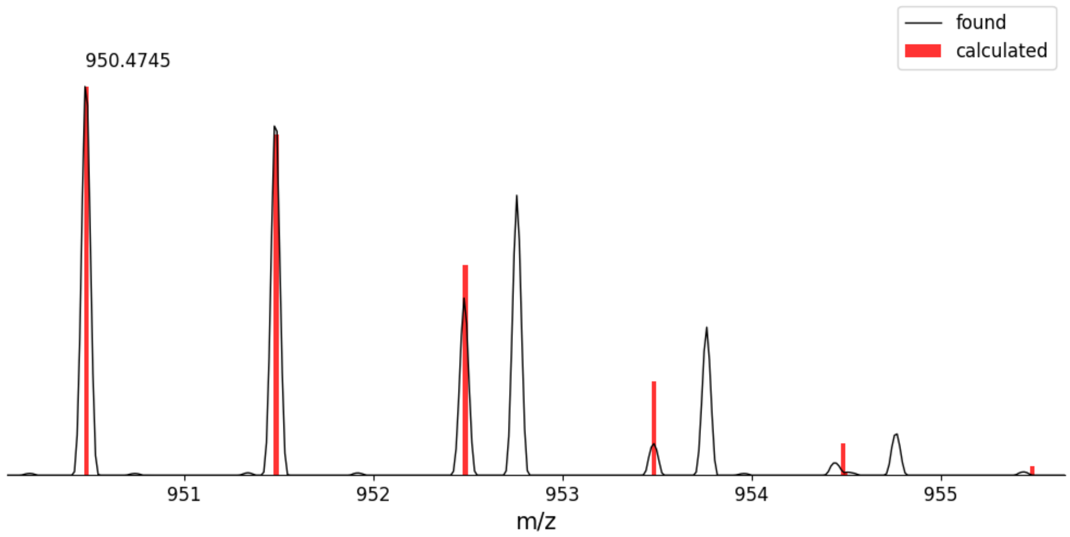

Supplementary Figure 50: LIFDI-MS spectrum for compound **4**.

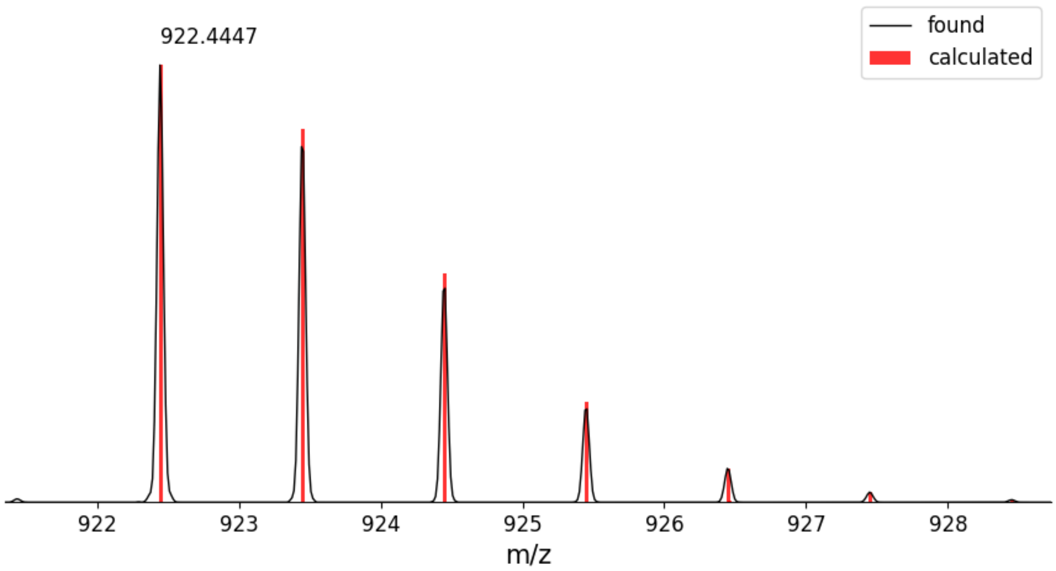

Supplementary Figure 51: LIFDI-MS spectrum for compound **5**.

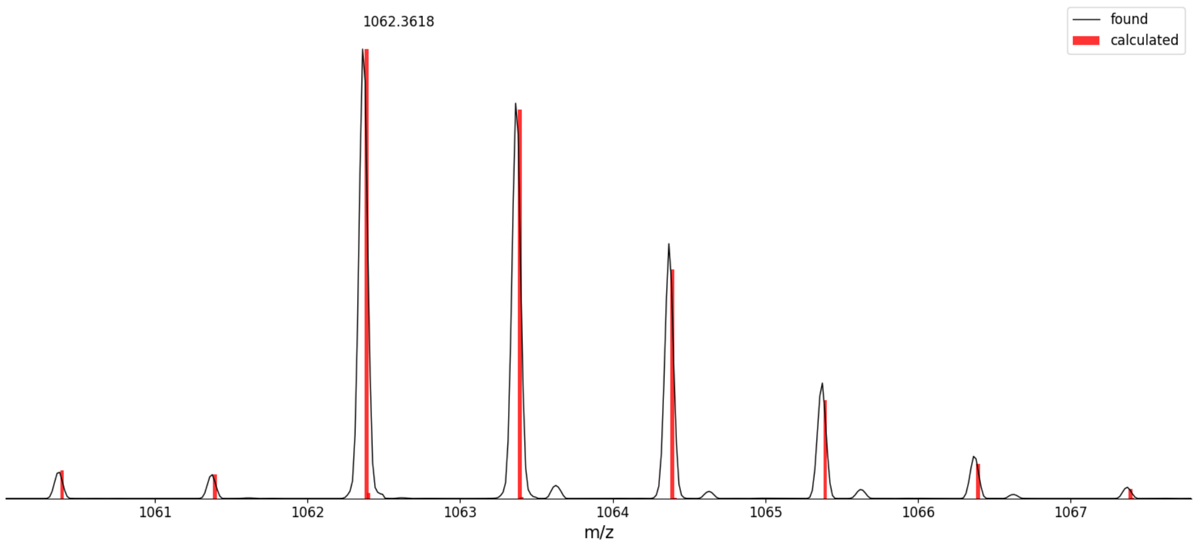

Supplementary Figure 52: LIFDI-MS spectrum for compound **6**.

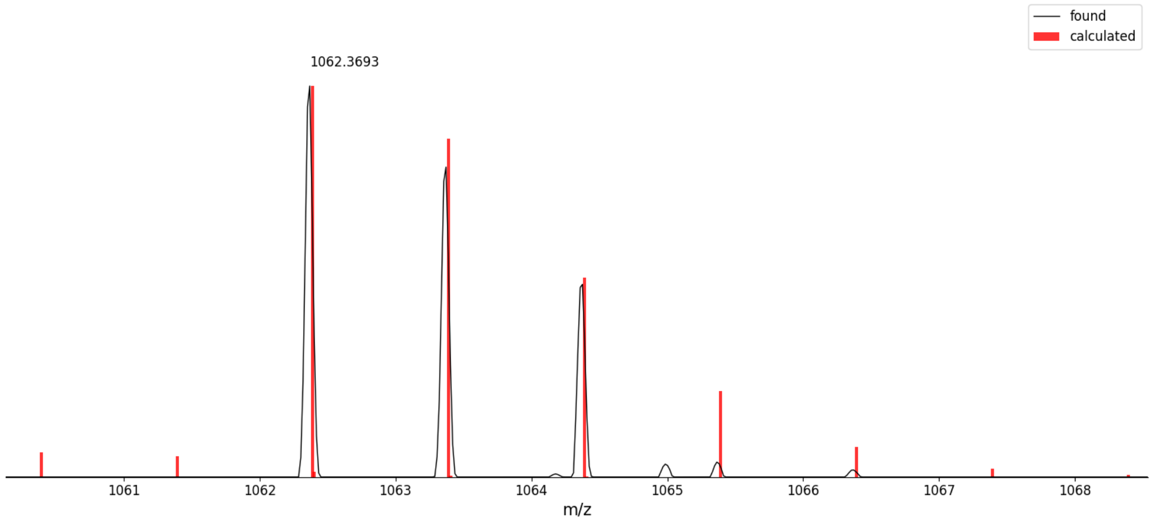

Supplementary Figure 53: LIFDI-MS spectrum for compound **7**.

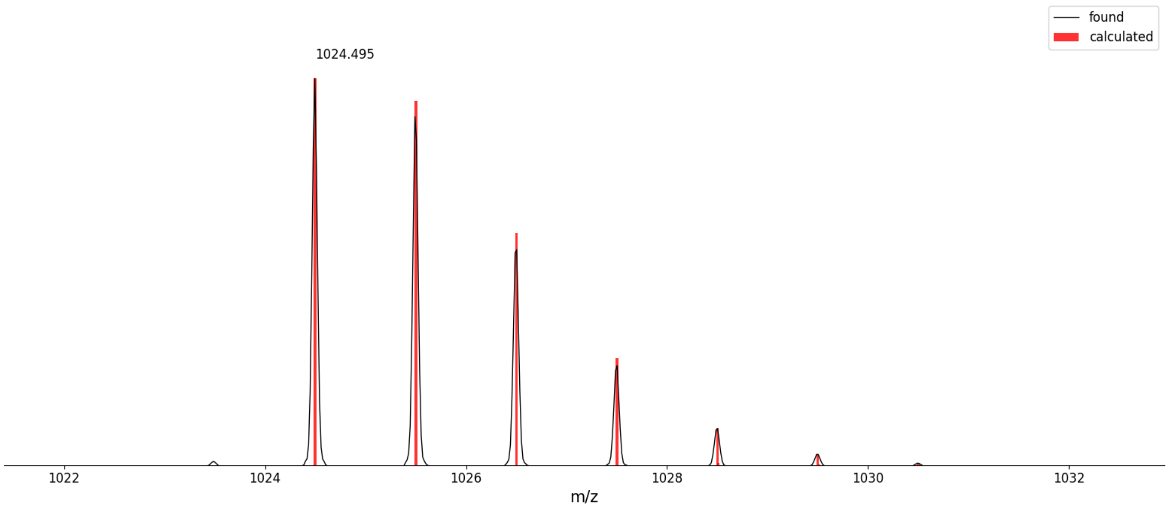

Supplementary Figure 54: LIFDI-MS spectrum for compound **8a**.

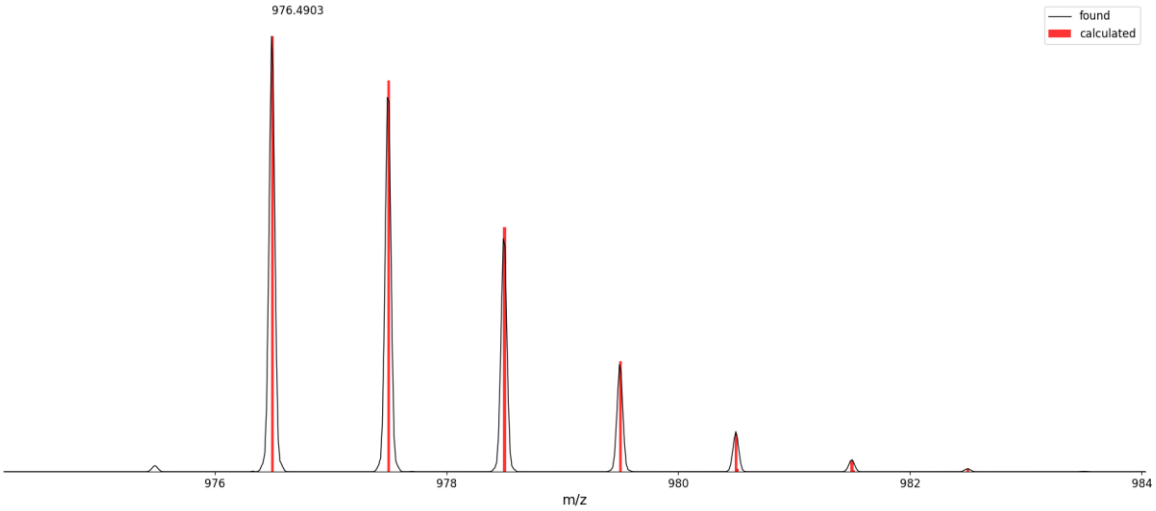

Supplementary Figure 55: LIFDI-MS spectrum for compound **8b**.

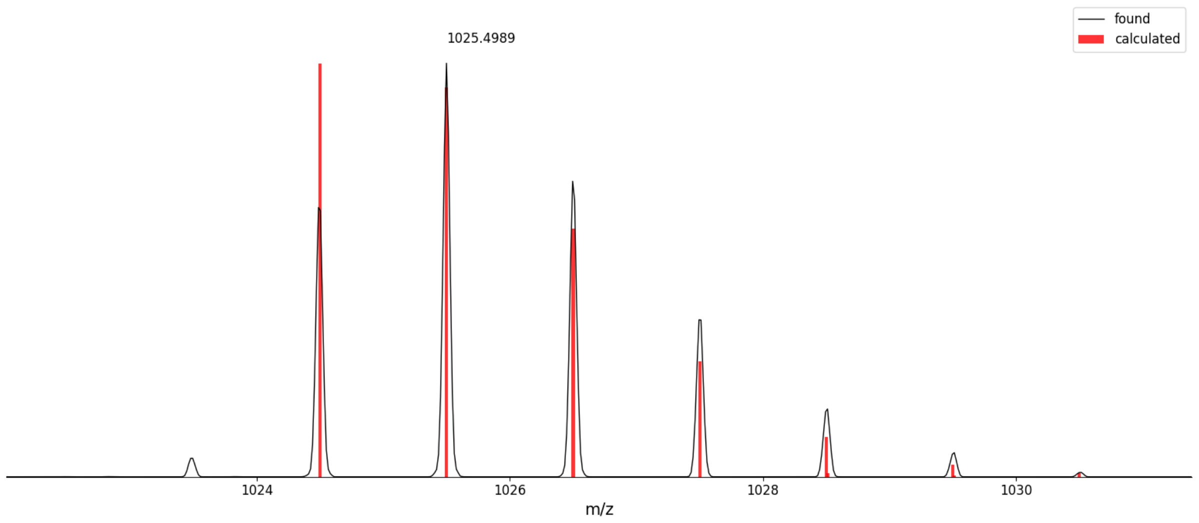

Supplementary Figure 56: LIFDI-MS spectrum for compound **9a**.

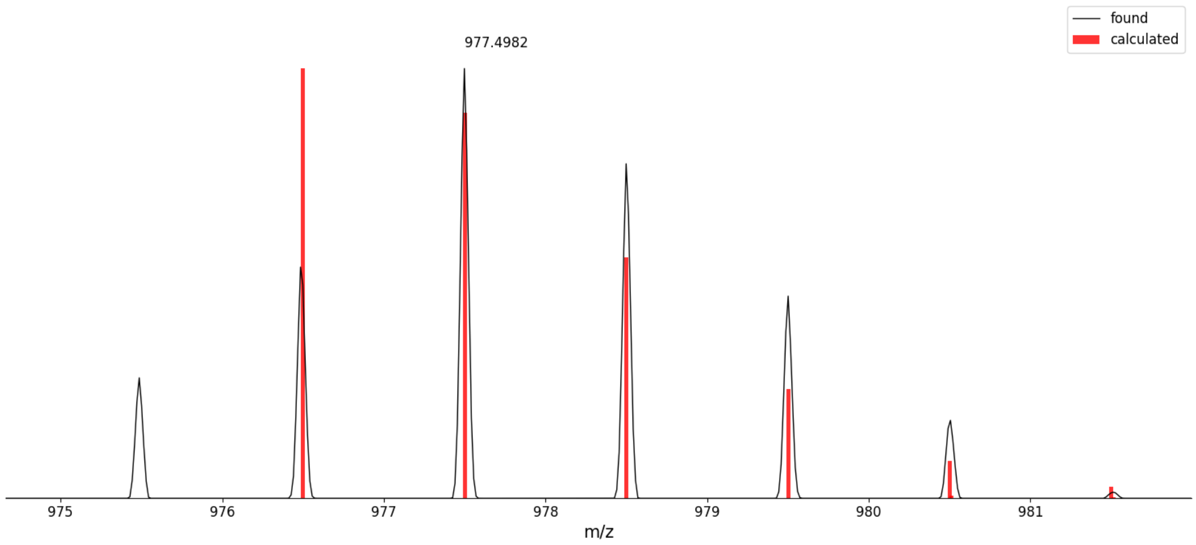

Supplementary Figure 57: LIFDI-MS spectrum for compound **9b**.

IR

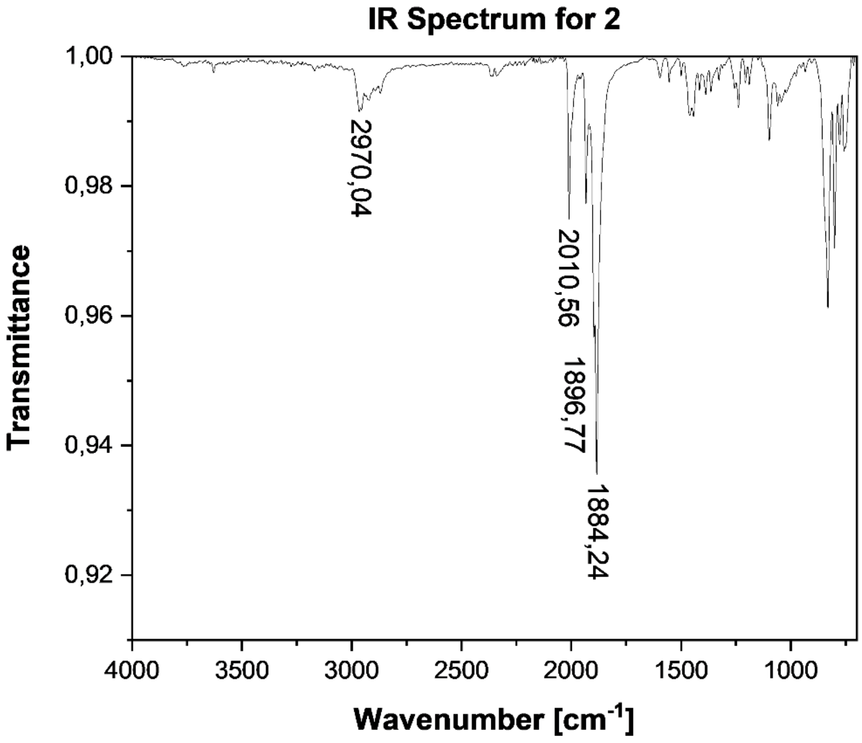

Supplementary Figure 58: IR spectrum of compound **2**.

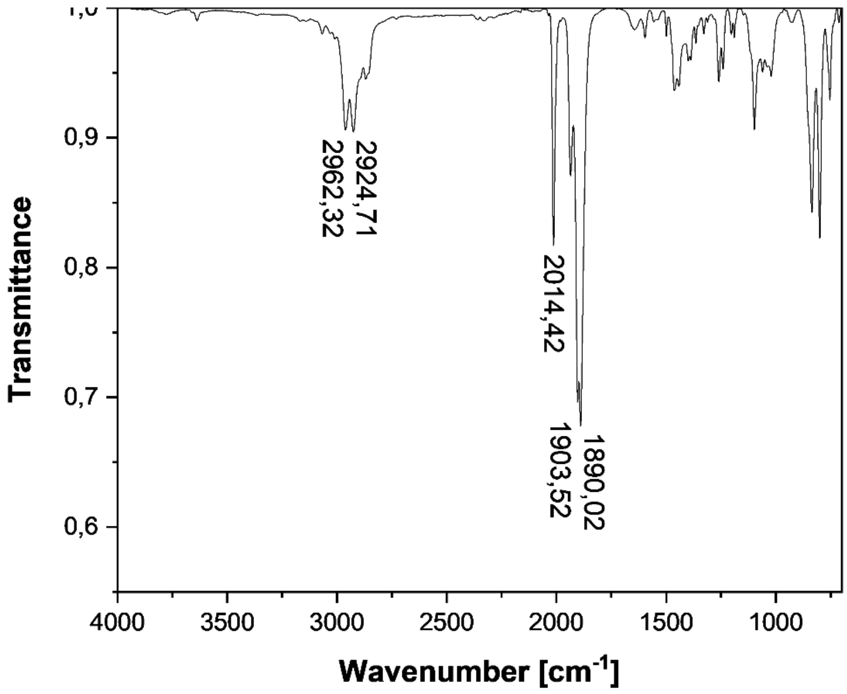

Supplementary Figure 59: IR spectrum of compound **5**.

UV-VIS

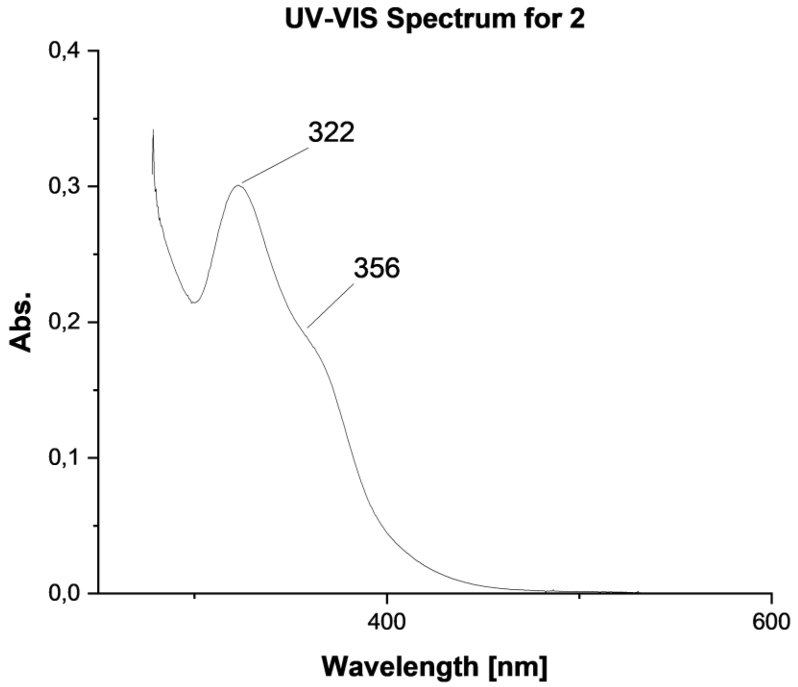

Supplementary Figure 60: UV-Vis spectrum of compound **2** (c = 5.4·10^{-4} M).

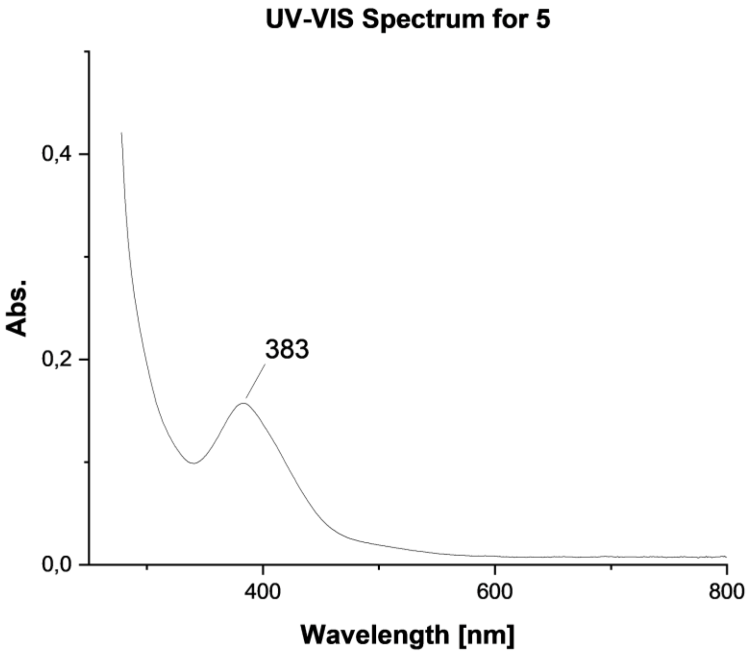

Supplementary Figure 61: UV-Vis spectrum of compound **5** (c = 5.4·10^{-4} M).

Additional Spectra of Precursors

$HSiTol_3$

^{1}H NMR (400 MHz, $CDCl_3$): δ 7.46 (d, *J* = 7.9 Hz, 6H), 7.18 (d, *J* = 7.5 Hz, 6H), 5.41 (s, 1H), 2.36 (s, 9H).

$ClSiTol_3$

^{1}H NMR (500 MHz, C_6D_6): δ 7.71 (d, *J* = 8.0 Hz, 1H), 7.00 (d, *J* = 7.6 Hz, 1H), 2.05 (s, 2H).

^{13}C NMR (126 MHz, C_6D_6): δ 140.89, 135.80, 130.44, 129.28, 21.47.

^{29}Si NMR (99 MHz, C_6D_6): δ 2.39.

$KSiTMS_2SiTol_3$

^{1}H NMR (500 MHz, THF): δ 7.47 (d, *J* = 8.0 Hz, 1H), 6.91 (d, *J* = 7.3 Hz, 0H), 2.24 (s, 2H), -0.08 (s, 3H).

^{13}C NMR (126 MHz, THF): δ 143.37, 137.65, 136.20, 129.82, 129.06, 127.99, 126.19, 21.71, 7.45.

^{29}Si NMR (99 MHz, THF): δ -0.18, -6.59, -189.78.

IDipp·HCl

^{1}H NMR (400 MHz, $CDCl_3$): δ 10.11 (s, 1H), 8.14 (d, *J* = 1.6 Hz, 5H), 7.57 (t, *J* = 7.8 Hz, 5H), 7.34 (d, *J* = 7.9 Hz, 8H), 2.44 (hept, *J* = 6.8 Hz, 7H), 1.28 (d, *J* = 6.9 Hz, 23H), 1.24 (d, *J* = 6.9 Hz, 25H).

IDippPH

^{1}H NMR (400 MHz, C_6D_6): δ 7.23 (dd, *J* = 8.5, 6.9 Hz, 1H), 7.14 (d, *J* = 7.7 Hz, 2H), 6.18 (s, 1H), 3.06 (hept, *J* = 6.9 Hz, 2H), 1.92 (d, *J* = 165.2 Hz, 1H), 1.47 (d, *J* = 6.9 Hz, 6H), 1.15 (d, *J* = 7.0 Hz, 6H).

^{31}P NMR (162 MHz, C_6D_6): δ -134.38.

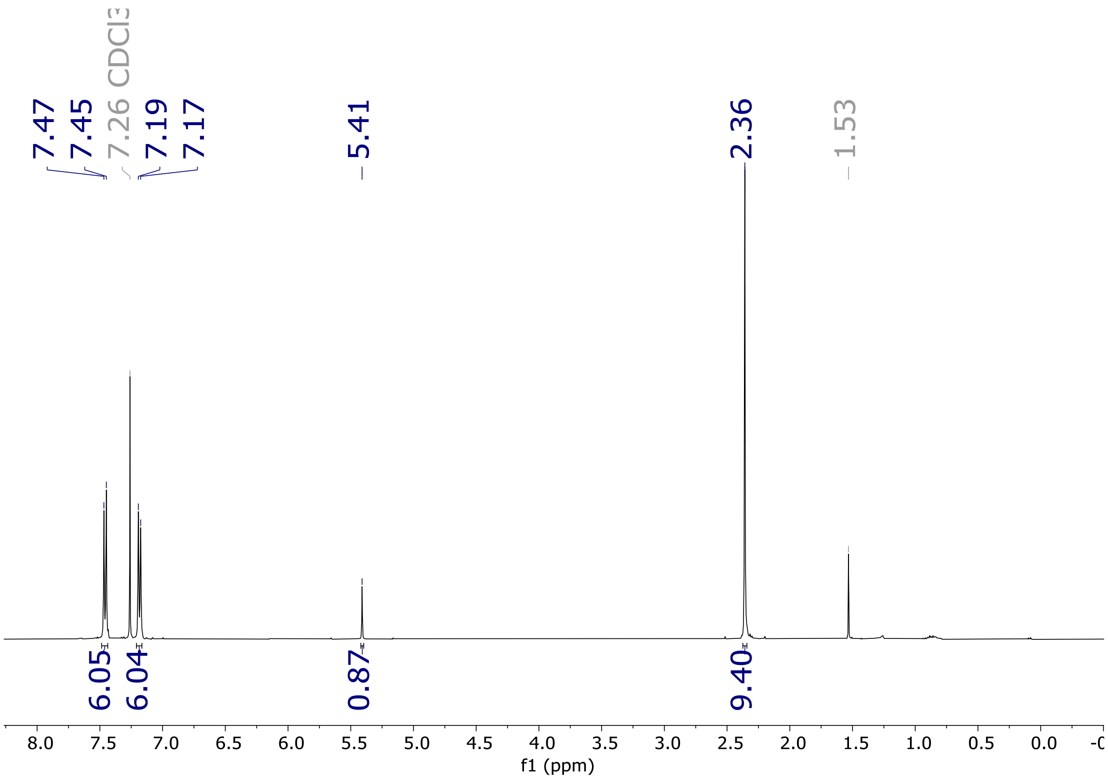

Supplementary Figure 62: ^{1}H NMR spectrum of $HSiTol_3$.

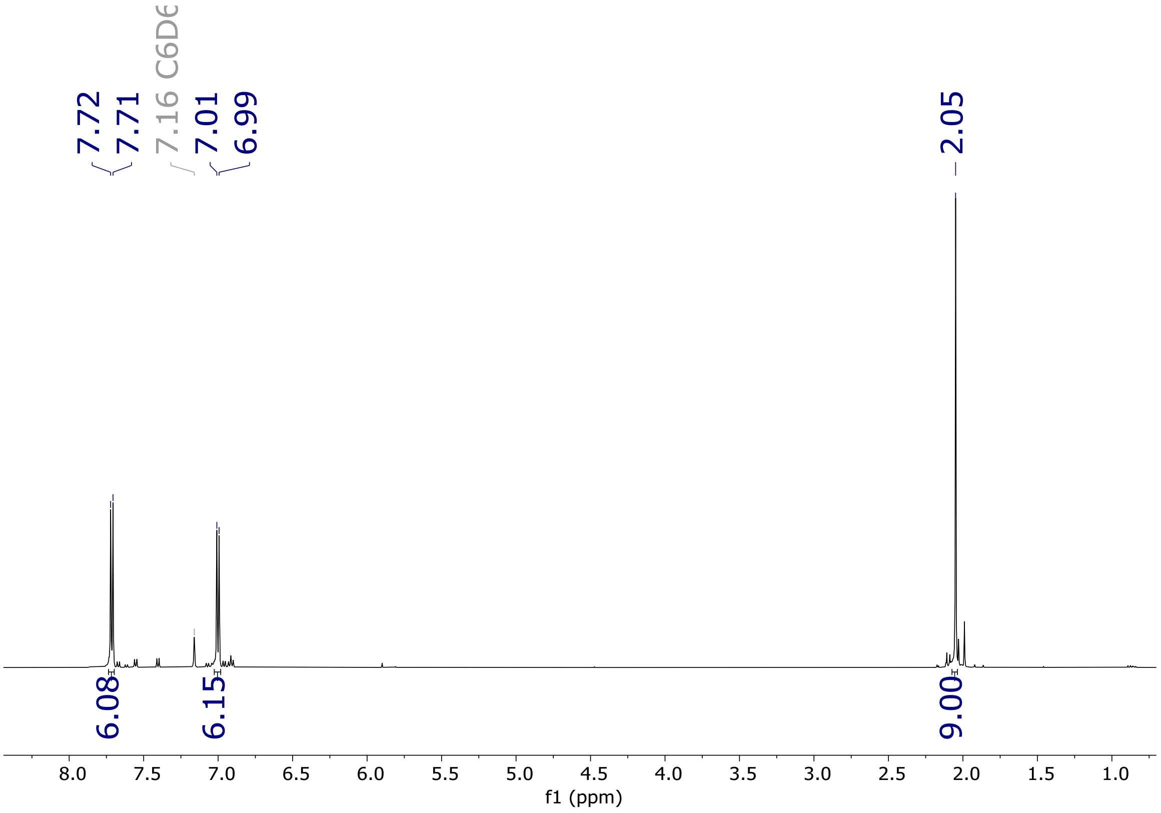

Supplementary Figure 63: ^{1}H NMR spectrum of $ClSiTol_3$.

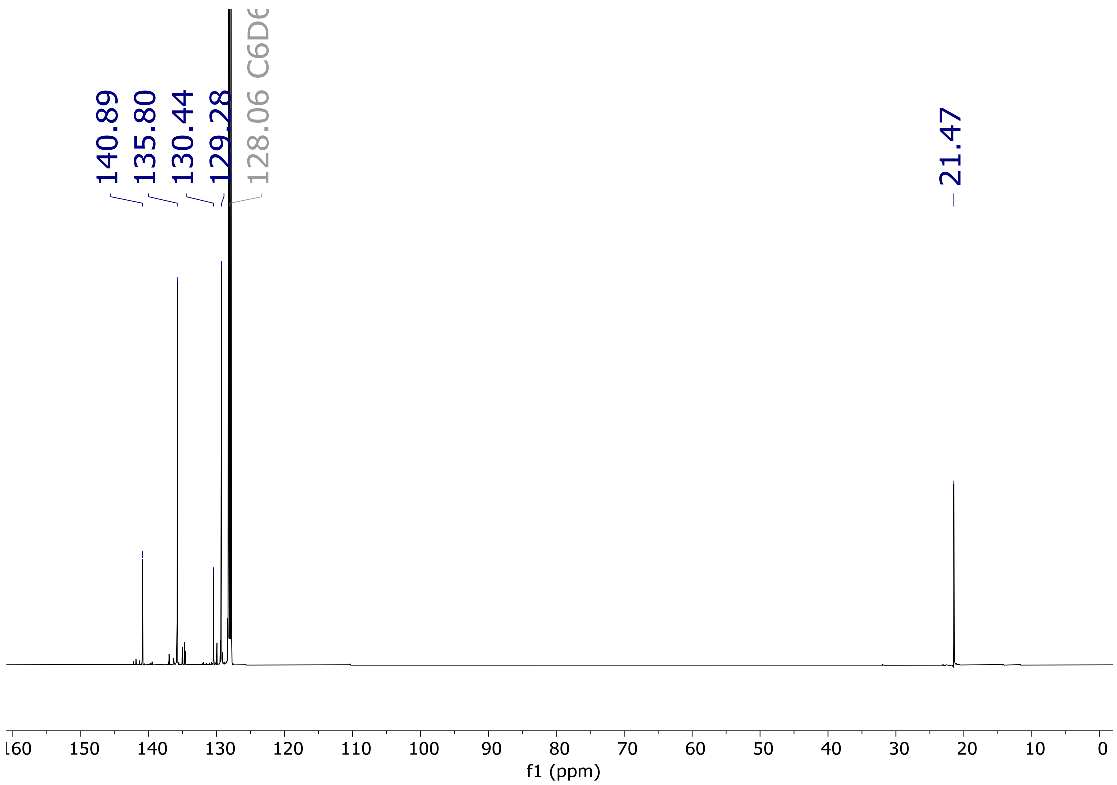

Supplementary Figure 64: ^{13}C NMR spectrum of $ClSiTol_3$.

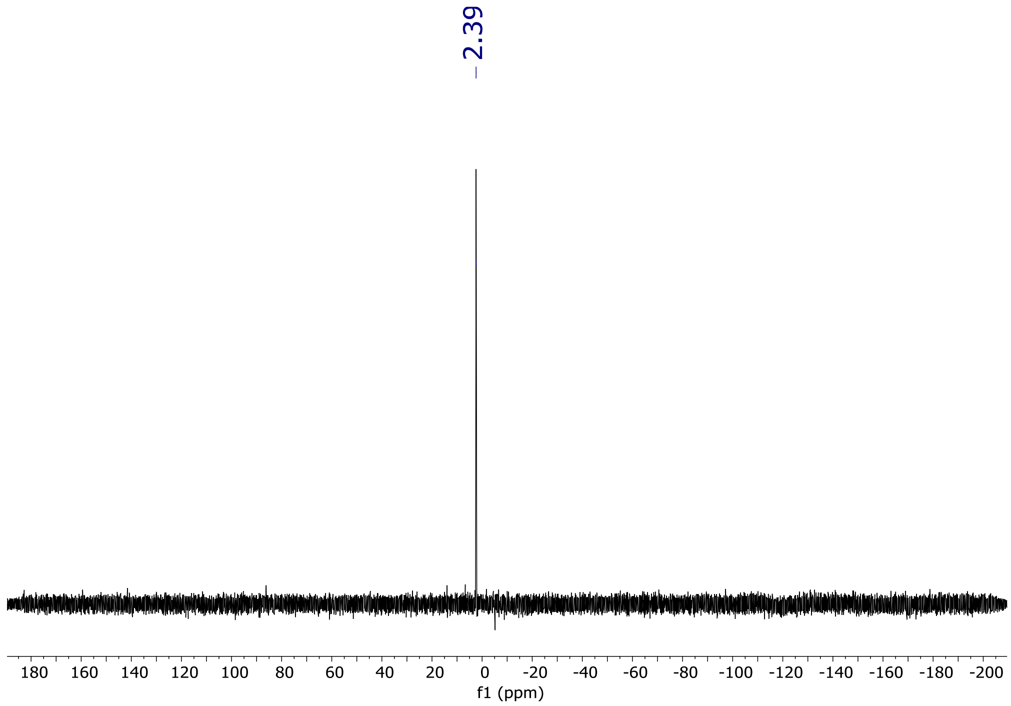

Supplementary Figure 65: ^{29}Si NMR spectrum of $ClSiTol_3$.

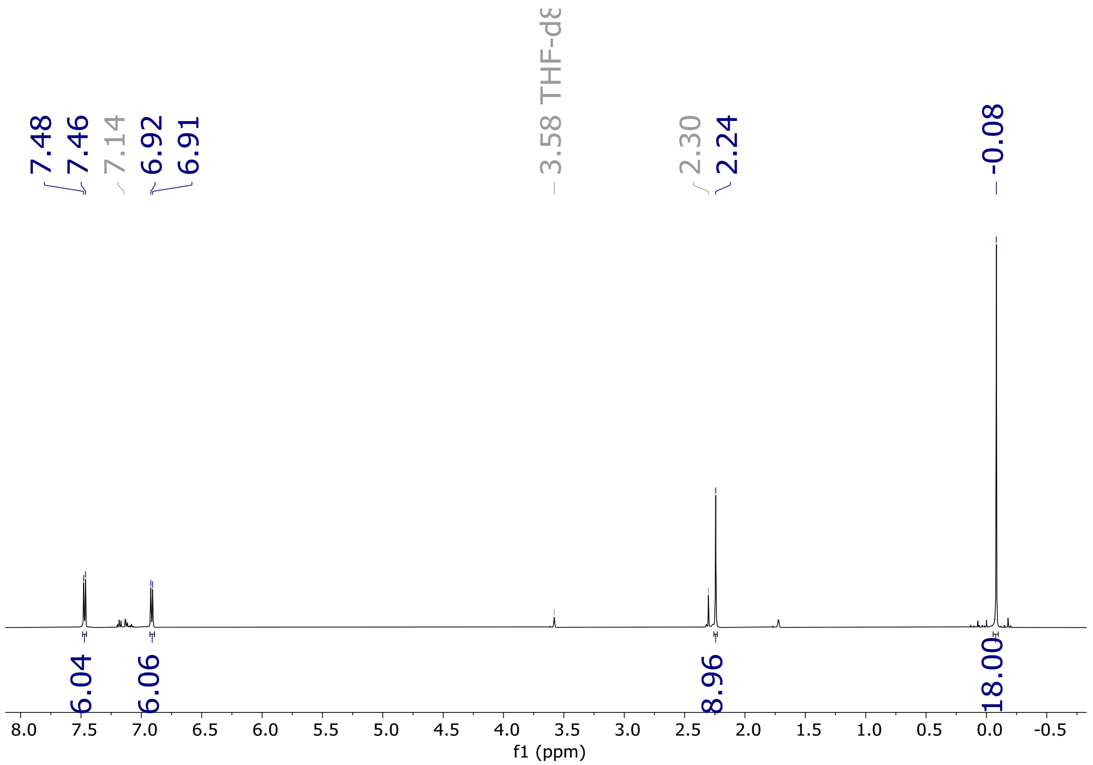

Supplementary Figure 66: ^{1}H NMR spectrum of $KSiTMS_2SiTol_3$. (grey signals are co-crystallized toluene)

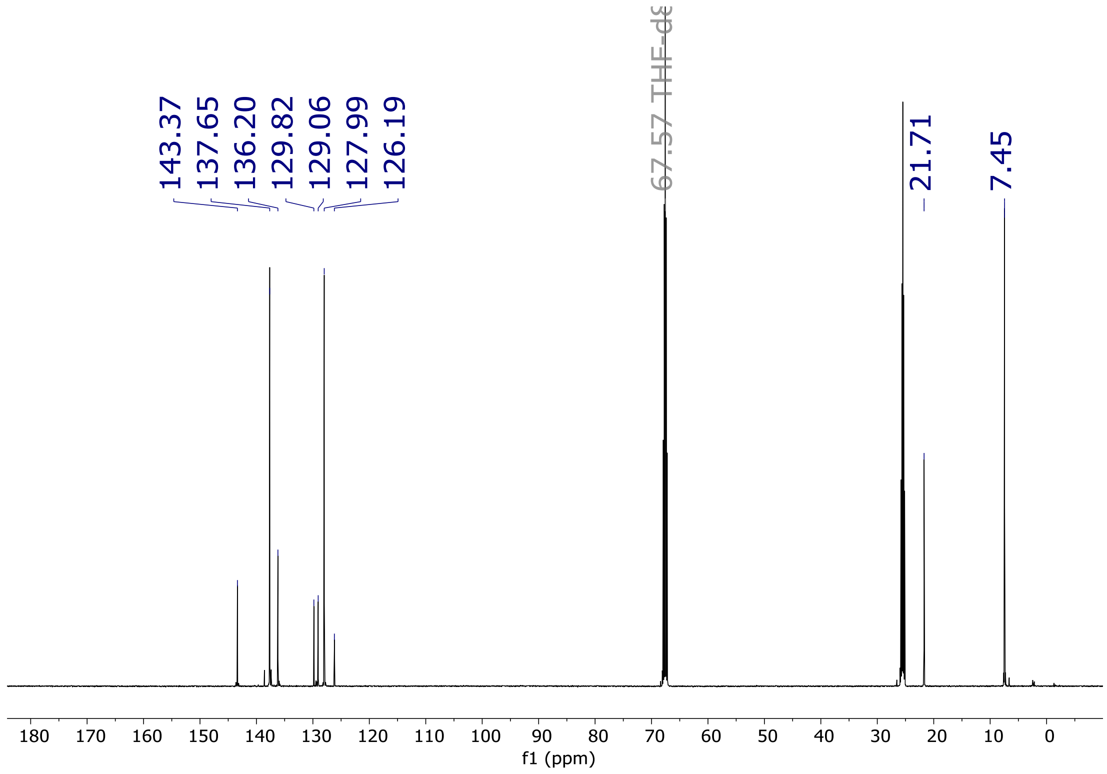

Supplementary Figure 67: ^{13}C NMR spectrum of $KSiTMS_2SiTol_3$ with some co-crystallized toluene.

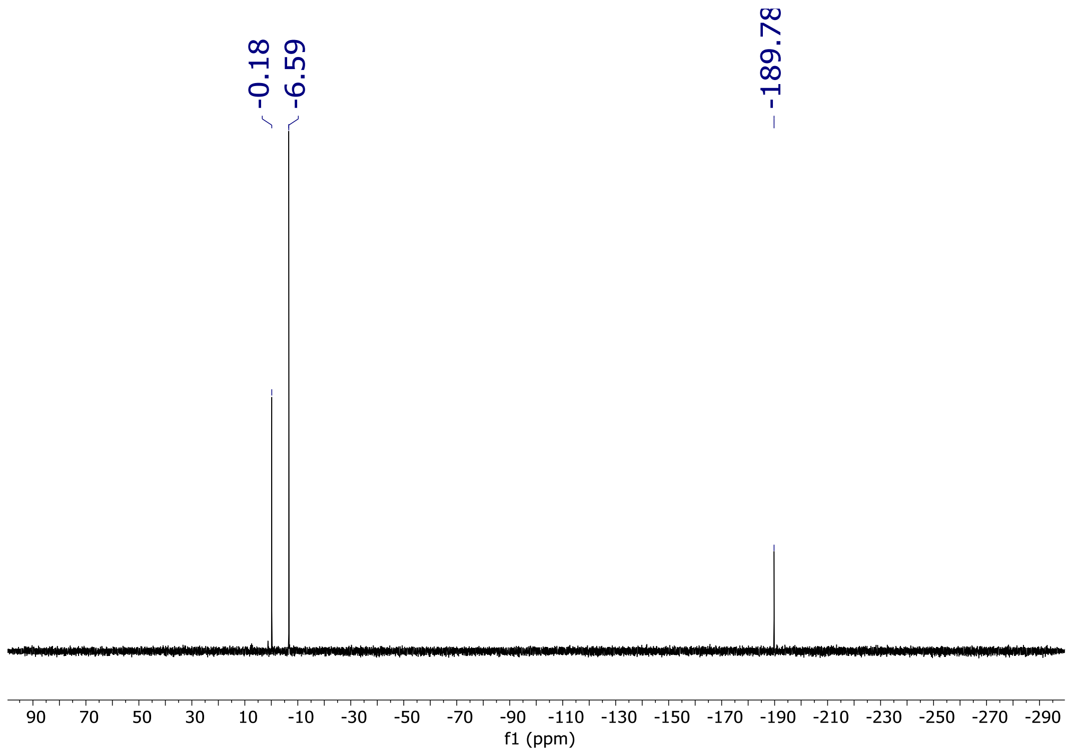

Supplementary Figure 68: ^{29}Si NMR spectrum of $KSiTMS_2SiTol_3$.

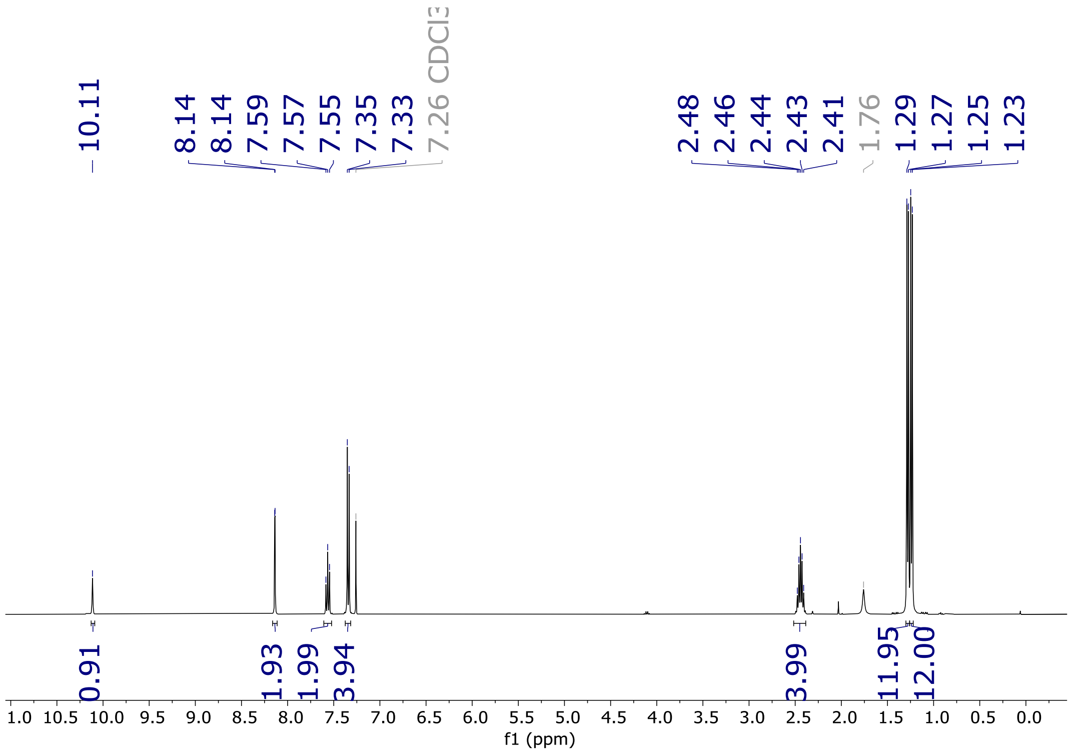

Supplementary Figure 69: ^{1}H NMR spectrum of IDippHCl.

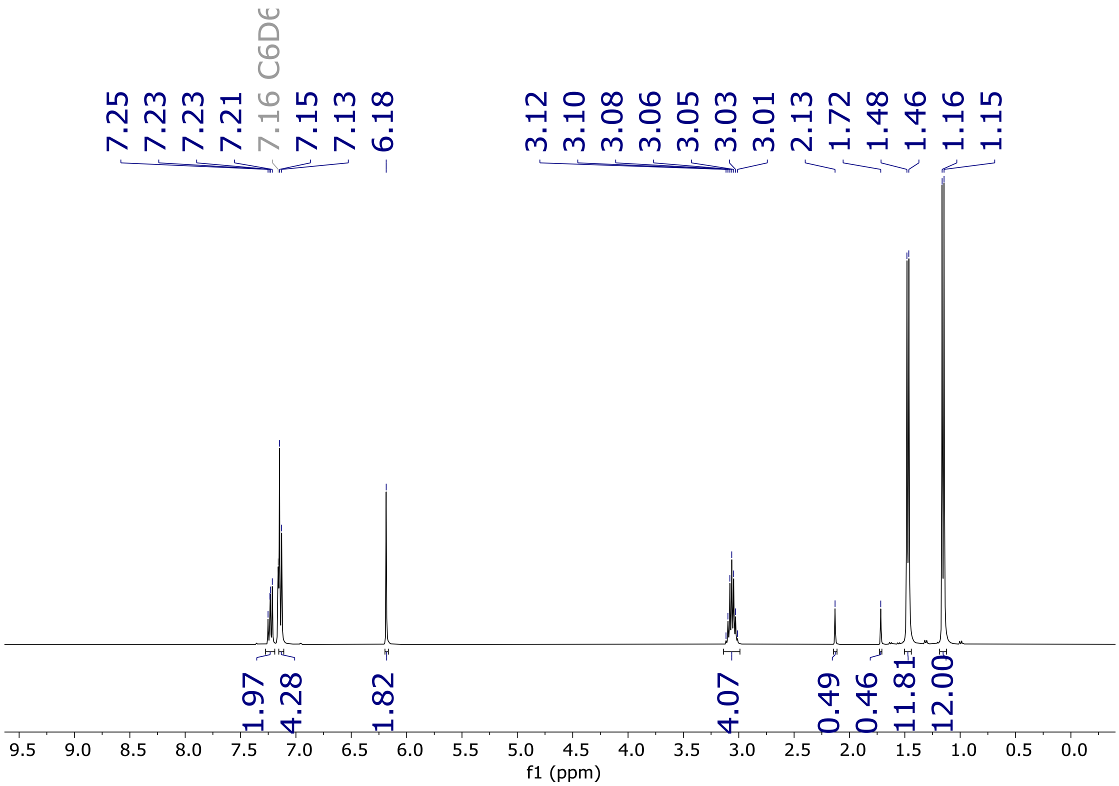

Supplementary Figure 70: ^{1}H NMR spectrum of IDippPH.

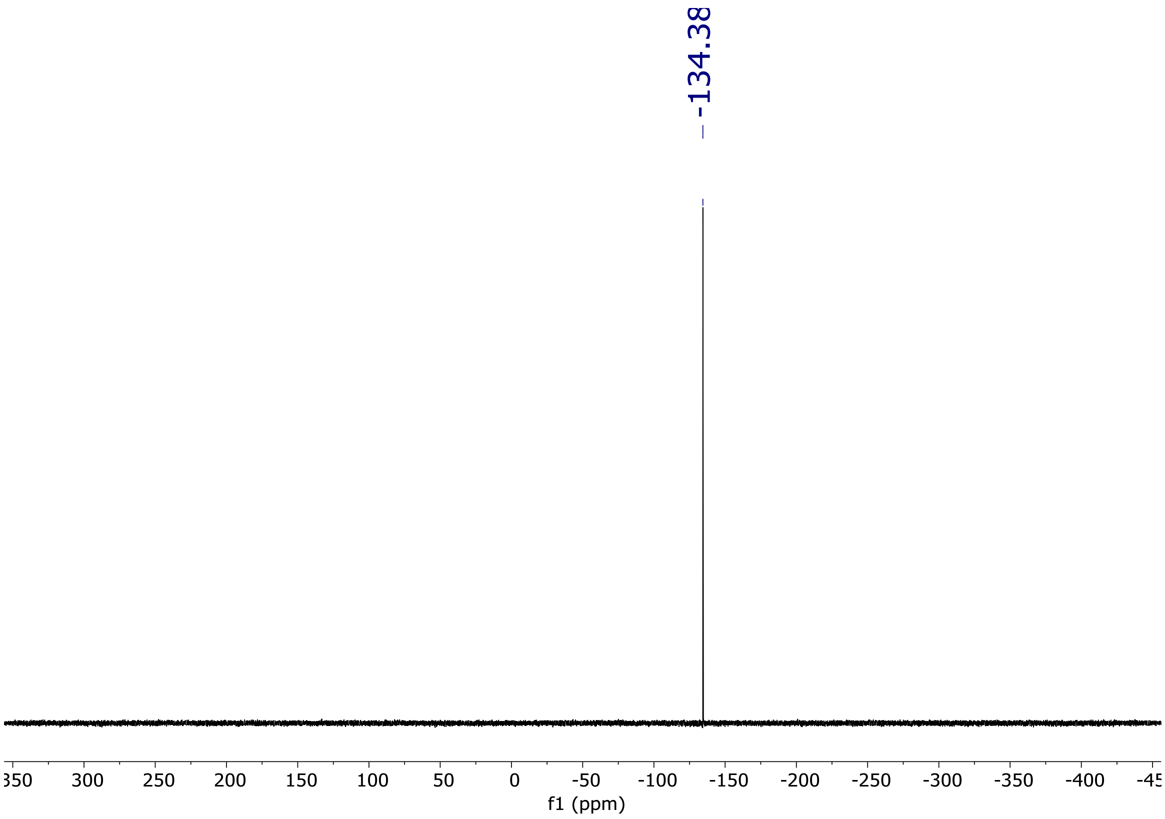

Supplementary Figure 71: ^{31}P NMR spectrum of IDippPH.

4. X-Ray Crystallographic Data

Compound **2** (CCDC 2325629)

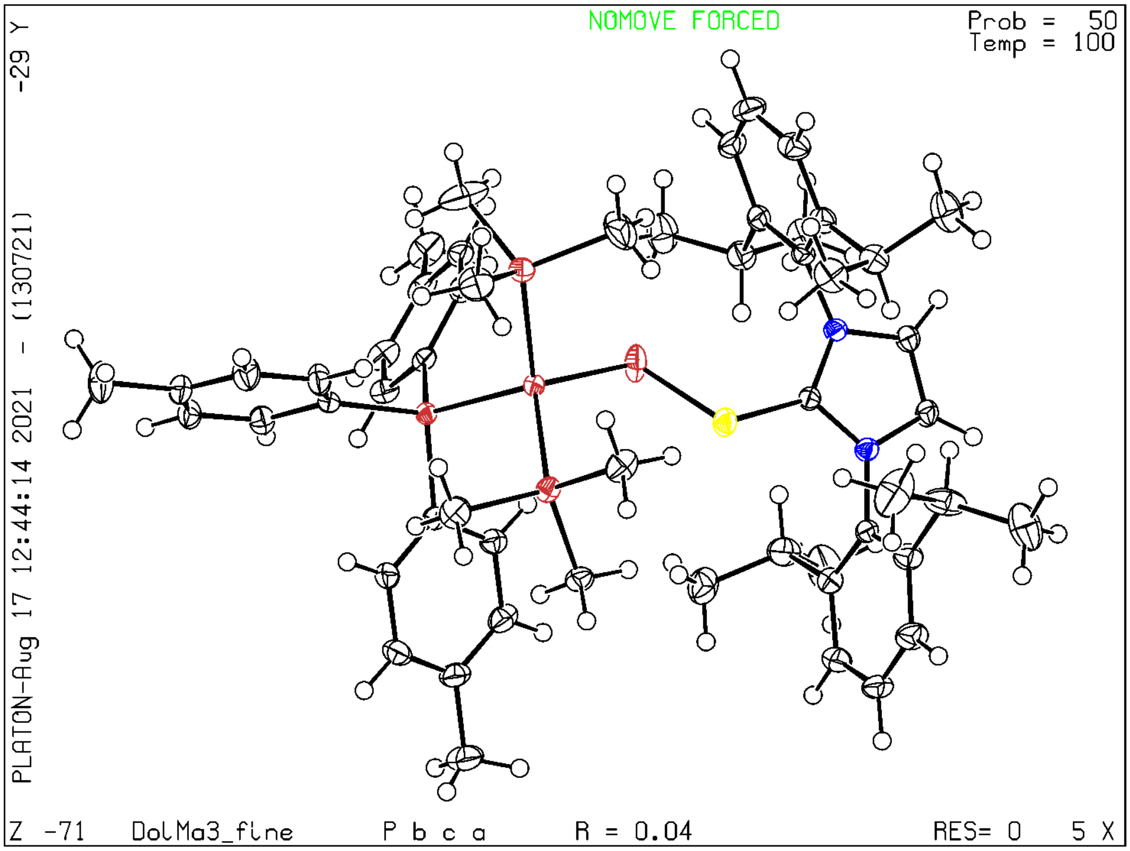

Supplementary Figure 72: Molecular structure of compound **2** generated in PLATON.

Diffractometer operator F. Hanusch
scanspeed 10s per frame dx 47
1414 frames measured in 9 data sets
phi-scans with delta_phi = 0.5
omega-scans with delta_omega = 0.5
shutterless mode

Crystal data

$C_{54}H_{75}N_2PSi_5$	
M_r = 923.58	D_x = 1.110 Mg m^{-3}
Orthorhombic, *Pbca*	
Hall symbol: -P 2ac 2ab	Mo *K*α radiation, λ = 0.71073 Å
a = 12.3178 (6) Å	Cell parameters from 9199 reflections
b = 24.1671 (11) Å	θ = 2.4–25.4°

c = 37.1392 (16) Å	μ = 0.19 mm^{-1}
V = 11055.8 (9) $Å^3$	T = 100 K
Z = 8	Fragment, clear green brown
$F(000)$ = 3984	0.59 × 0.48 × 0.27 mm

Data collection

Bruker Photon CMOS diffractometer	10131 independent reflections
Radiation source: IMS microsource	9151 reflections with $I > 2\sigma(I)$
Helios optic monochromator	R_{int} = 0.039
Detector resolution: 16 pixels mm^{-1}	θ_{max} = 25.4°, θ_{min} = 1.9°
phi– and ω–rotation scans	h = −14 14
Absorption correction: multi-scan *SADABS* 2016/2, Bruker, 2016	k = −29 29
T_{min} = 0.692, T_{max} = 0.745	l = −44 44
126574 measured reflections	

Refinement

Refinement on F^2	
Least-squares matrix: full	Hydrogen site location: inferred from neighbouring sites
$R[F^2 > 2\sigma(F^2)]$ = 0.041	H-atom parameters constrained
$wR(F^2)$ = 0.100	W = $1/[\Sigma^2(FO^2) + (0.040P)^2 + 11.0092P]$ WHERE $P = (FO^2 + 2FC^2)/3$
S = 1.06	$(\Delta/\sigma)_{max}$ = 0.001
10131 reflections	$\Delta\rho_{max}$ = 1.34 e $Å^{-3}$
576 parameters	$\Delta\rho_{min}$ = −0.40 e $Å^{-3}$
36 restraints	
Primary atom site location: iterative	

Compound **3** (CCDC 2325631)

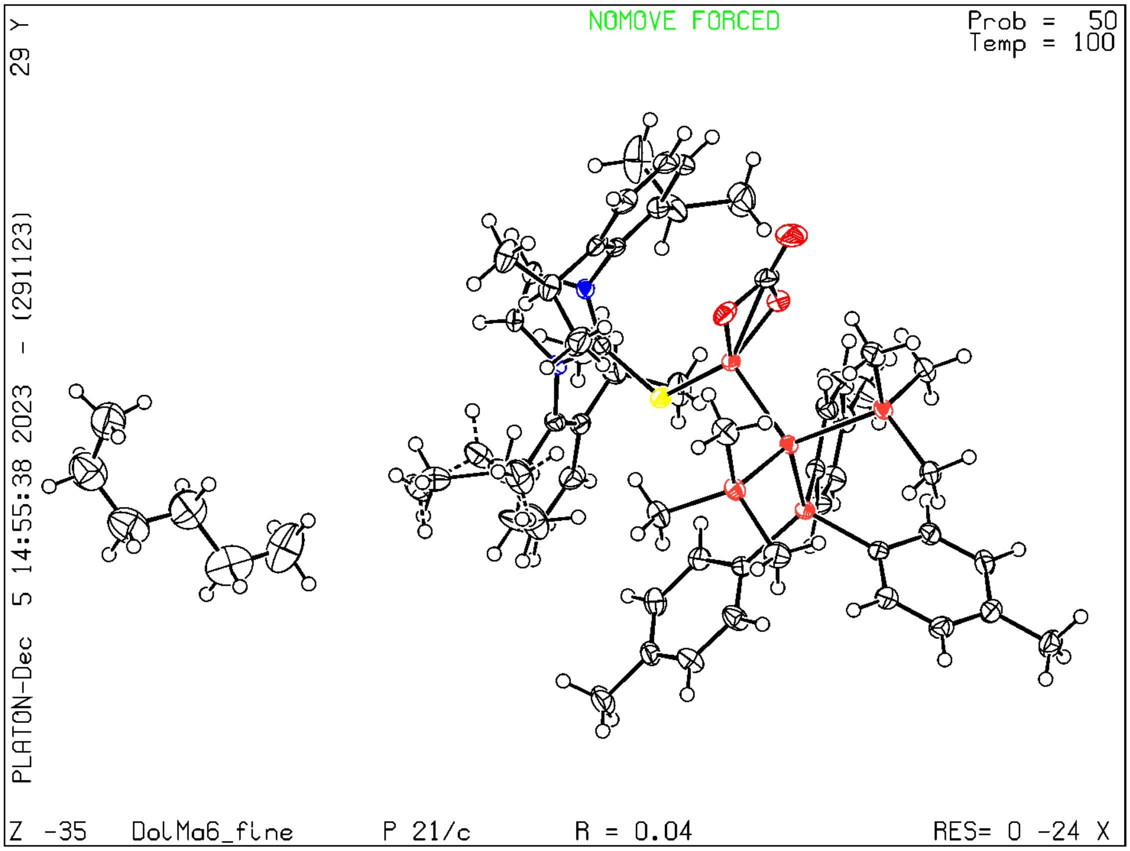

Supplementary Figure 73: Molecular structure of compound **2** generated in PLATON.

Diffractometer operator F. Hanusch
scanspeed 3s per frame dx 42
2283 frames measured in 6 data sets
phi-scans with delta_phi = 0.5
omega-scans with delta_omega = 0.5
shutterless mode

Crystal data

$C_{55}H_{75}N_2O_3PSi_5{\cdot}C_6H_{14}$	
M_r = 1069.76	D_x = 1.145 Mg m^{-3}
Monoclinic, $P2_1/c$	
Hall symbol: -P 2ybc	Mo *Kα* radiation, λ = 0.71073 Å
a = 13.7986 (10) Å	Cell parameters from 9964 reflections
b = 21.0946 (13) Å	θ = 2.5–25.4°
c = 21.4492 (16) Å	μ = 0.18 mm^{-1}
β = 96.305 (3)°	*T* = 100 K

V = 6205.6 (8) Å^3	Fragment, clear orange brown
Z = 4	0.25 × 0.23 × 0.18 mm
F(000) = 2312	

Data collection

Bruker Photon CMOS diffractometer	11430 independent reflections
Radiation source: TXS rotating anode	9689 reflections with $I > 2\sigma(I)$
Helios optic monochromator	R_{int} = 0.056
Detector resolution: 16 pixels mm^{-1}	θ_{max} = 25.4°, θ_{min} = 2.1°
phi– and ω–rotation scans	h = −16 16
Absorption correction: multi-scan *SADABS* 2016/2, Bruker, 2016	k = −25 24
T_{min} = 0.720, T_{max} = 0.745	l = −25 25
140630 measured reflections	

Refinement

Refinement on F^2	
Least-squares matrix: full	Hydrogen site location: inferred from neighbouring sites
$R[F^2 > 2\sigma(F^2)]$ = 0.044	H-atom parameters constrained
$wR(F^2)$ = 0.121	W = 1/[Σ^2(FO^2) + (0.0589P)^2 + 11.0017P] WHERE P = (FO^2 + 2FC^2)/3
S = 0.89	$(\Delta/\sigma)_{max}$ = 0.007
11430 reflections	$\Delta\rho_{max}$ = 0.93 e Å^{-3}
698 parameters	$\Delta\rho_{min}$ = −0.50 e Å^{-3}
162 restraints	
Primary atom site location: structure-invariant direct methods	

Compound **4** (CCDC 2325630)

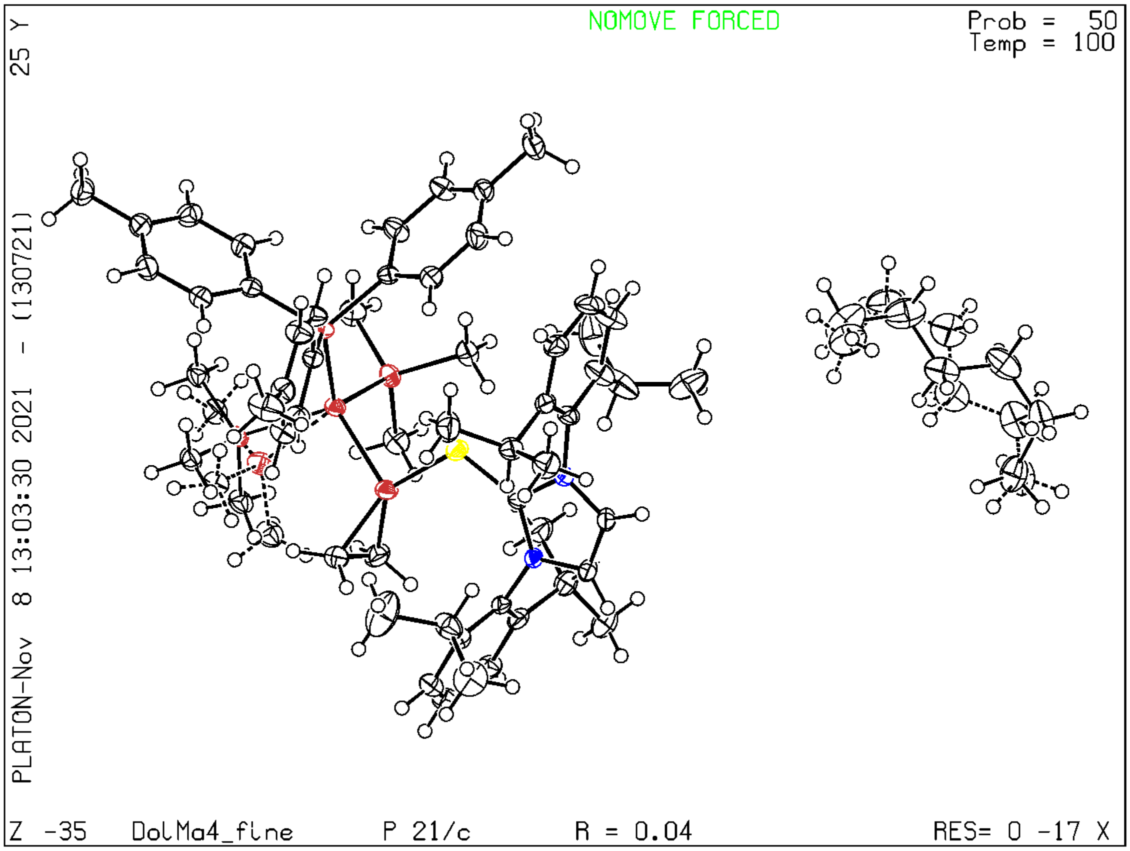

Supplementary Figure 74: Molecular structure of compound **4** generated in PLATON.

Diffractometer operator F. Hanusch
scanspeed 3s per frame dx 40
5866 frames measured in 20 data sets
phi-scans with delta_phi = 0.5
omega-scans with delta_omega = 0.5
shutterless mode

Crystal data

$C_{56}H_{79}N_2PSi_5{\cdot}C_6H_{14}$	
M_r = 1037.80	D_x = 1.113 Mg m^{-3}
Monoclinic, $P2_1/c$	
Hall symbol: -P 2ybc	Cu $K\alpha$ radiation, λ = 1.54178 Å
a = 13.7643 (4) Å	Cell parameters from 9765 reflections
b = 21.3586 (6) Å	θ = 3.0–68.3°
c = 21.1733 (6) Å	μ = 1.60 mm^{-1}
β = 95.956 (1)°	T = 100 K

V = 6191.1 (3) Å^3	Fragment, clear brown
Z = 4	0.36 × 0.28 × 0.23 mm
F(000) = 2256	

Data collection

Bruker Photon CMOS diffractometer	11324 independent reflections
Radiation source: IMS microsource	10548 reflections with $I > 2\sigma(I)$
Helios optic monochromator	R_{int} = 0.036
Detector resolution: 16 pixels mm^{-1}	θ_{max} = 68.2°, θ_{min} = 3.0°
phi– and ω–rotation scans	h = −16 16
Absorption correction: multi-scan *SADABS* 2016/2, Bruker, 2016	k = −25 25
T_{min} = 0.561, T_{max} = 0.753	l = −25 25
130990 measured reflections	

Refinement

Refinement on F^2

Least-squares matrix: full	Hydrogen site location: inferred from neighbouring sites
$R[F^2 > 2\sigma(F^2)]$ = 0.042	H-atom parameters constrained
$wR(F^2)$ = 0.121	W = $1/[\Sigma^2(FO^2) + (0.068P)^2 + 4.8225P]$ WHERE $P = (FO^2 + 2FC^2)/3$
S = 1.02	$(\Delta/\sigma)_{max}$ = 0.001
11324 reflections	$\Delta\rho_{max}$ = 0.82 e Å^{-3}
737 parameters	$\Delta\rho_{min}$ = −0.57 e Å^{-3}
255 restraints	

Primary atom site location: iterative

Compound **5** (CCDC 2325632)

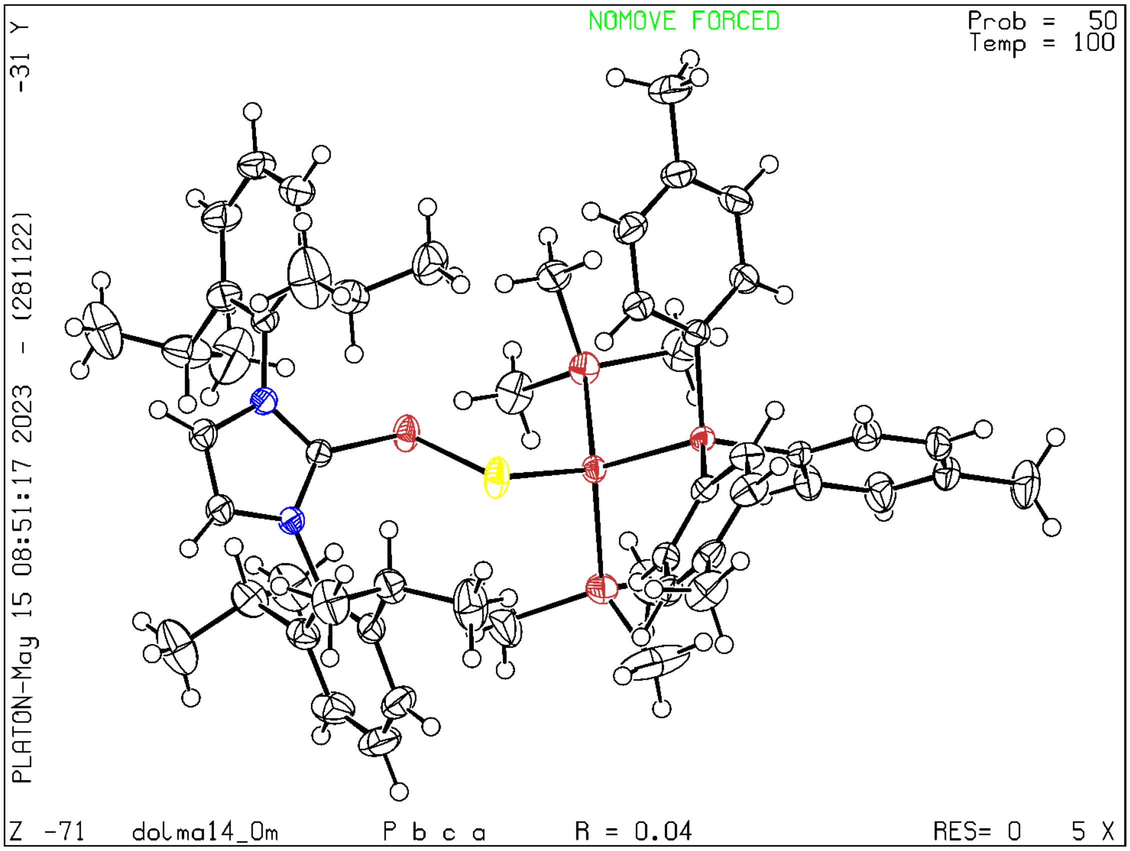

Supplementary Figure 75: Molecular structure of compound **5** generated in PLATON.

Diffractometer operator Jin Yu Liu
scan speed 5s per frame dx 40 mm
2480 frames measured in 7 data sets
phi-scans with delta phi = 0.5
omega-scans with delta omega = 0.5
shutterless mode

Crystal data

$C_{54}H_{75}N_2PSi_5$

M_r = 923.58	D_x = 1.097 Mg m^{-3}
Orthorhombic, *Pbca*	
Hall symbol: -P 2ac 2ab	Mo *K*α radiation, λ = 0.71073 Å
a = 12.3808 (8) Å	Cell parameters from 9564 reflections

b = 24.1218 (14) Å	θ = 2.5–32.0°
c = 37.441 (2) Å	μ = 0.19 mm^{-1}
V = 11181.7 (11) Å^3	T = 100 K
Z = 8	Fragment, colorless
F(000) = 3984	0.48 × 0.26 × 0.25 mm

Data collection

Bruker Photon CMOS diffractometer	10597 independent reflections
Radiation source: TXS rotating anode	9839 reflections with $I > 2\sigma(I)$
Helios optic monochromator	R_{int} = 0.059
Detector resolution: 16 pixels mm^{-1}	θ_{max} = 25.7°, θ_{min} = 1.1°
phi– and ω–rotation scans	h = −15 15
Absorption correction: multi-scan *SADABS* 2016/2, Bruker, 2016	k = −29 29
T_{min} = 0.699, T_{max} = 0.746	l = −45 45
300272 measured reflections	

Refinement

Refinement on F^2	Secondary atom site location: difference Fourier map
Least-squares matrix: full	Hydrogen site location: inferred from neighbouring sites
$R[F^2 > 2\sigma(F^2)]$ = 0.036	H-atom parameters constrained
$wR(F^2)$ = 0.111	W = 1/[Σ^2(FO^2) + (0.0639P)^2 + 5.8156P] WHERE P = (FO^2 + 2FC^2)/3
S = 1.03	$(\Delta/\sigma)_{max}$ = 0.002
10597 reflections	$\Delta\rho_{max}$ = 0.44 e Å^{-3}
576 parameters	$\Delta\rho_{min}$ = −0.35 e Å^{-3}

Primary atom site location: iterative

Compound **7** (CCDC 2325633)

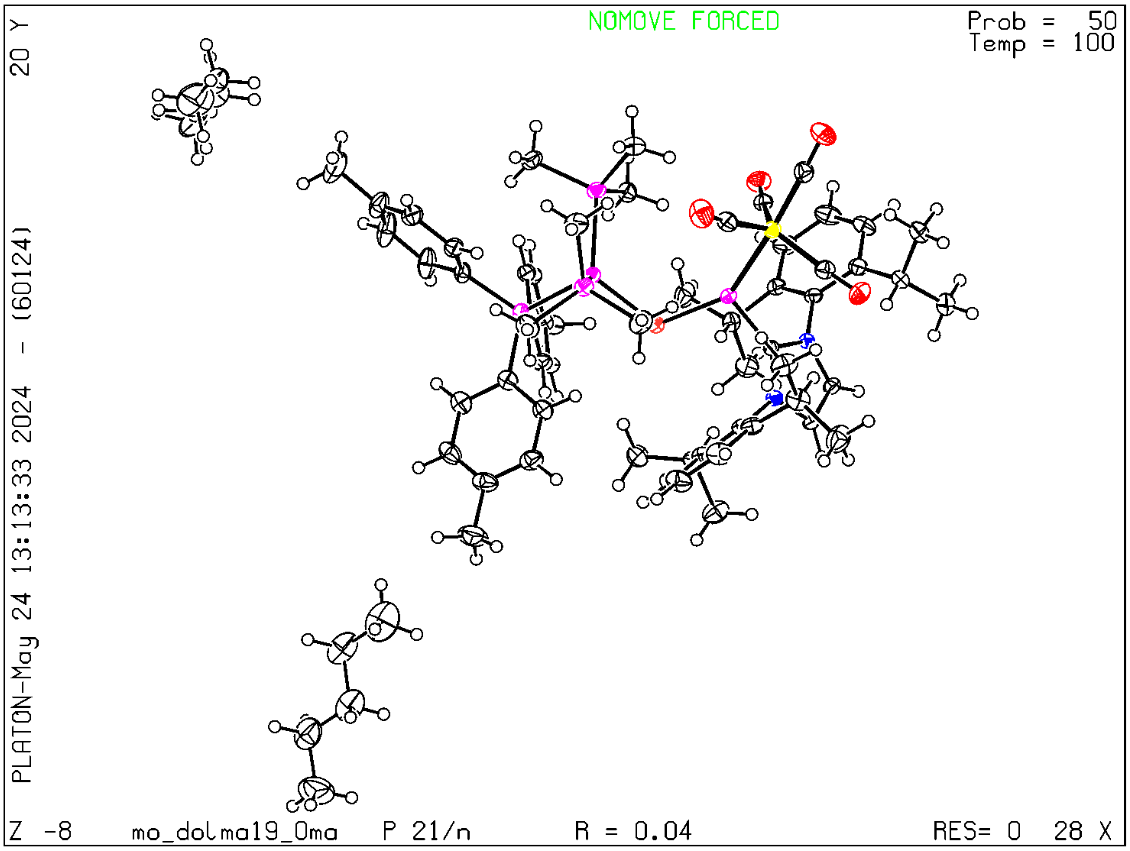

Supplementary Figure 76: Molecular structure of compound **7** generated in PLATON.

Diffractometer operator Jin Yu Liu
scanspeed 5s per frame dx 40mm
2936 frames measured in 7 data sets
phi-scans with delta_phi = 0.5
omega-scans with delta_omega = 0.5
shutterless mode

Crystal data

$C_{58}H_{75}FeN_2O_4PSi_5 \cdot C_5H_{12}$

M_r = 1163.62	D_x = 1.171 Mg m^{-3}
Monoclinic, $P2_1/n$	
Hall symbol: -P 2yn	Mo $K\alpha$ radiation, λ = 0.71073 Å
a = 14.9029 (6) Å	Cell parameters from 9056 reflections

b = 27.6947 (13) Å	θ = 2.5–26.4°
c = 16.1648 (7) Å	μ = 0.39 mm^{-1}
β = 98.276 (2)°	T = 100 K
V = 6602.2 (5) $Å^3$	Fragment, orange
Z = 4	0.31 × 0.28 × 0.17 mm
F(000) = 2488	

Data collection

Bruker Photon CMOS diffractometer	13606 independent reflections
Radiation source: TXS rotating anode	12464 reflections with $I > 2\sigma(I)$
Helios optic monochromator	R_{int} = 0.058
Detector resolution: 16 pixels mm^{-1}	θ_{max} = 26.5°, θ_{min} = 2.0°
phi– and ω–rotation scans	h = −18 18
Absorption correction: multi-scan *SADABS* 2016/2, Bruker, 2016	k = −34 34
T_{min} = 0.723, T_{max} = 0.745	l = −20 20
321157 measured reflections	

Refinement

Refinement on F^2	Secondary atom site location: difference Fourier map
Least-squares matrix: full	Hydrogen site location: inferred from neighbouring sites
$R[F^2 > 2\sigma(F^2)]$ = 0.040	H-atom parameters constrained
$wR(F^2)$ = 0.100	W = $1/[\Sigma^2(FO^2) + (0.0407P)^2 + 5.095P]$ WHERE $P = (FO^2 + 2FC^2)/3$
S = 1.14	$(\Delta/\sigma)_{max}$ = 0.001
13606 reflections	$\Delta\rho_{max}$ = 0.43 e $Å^{-3}$
751 parameters	$\Delta\rho_{min}$ = −0.34 e $Å^{-3}$
108 restraints	
Primary atom site location: iterative	

Compound **9a** (CCDC 2325634)

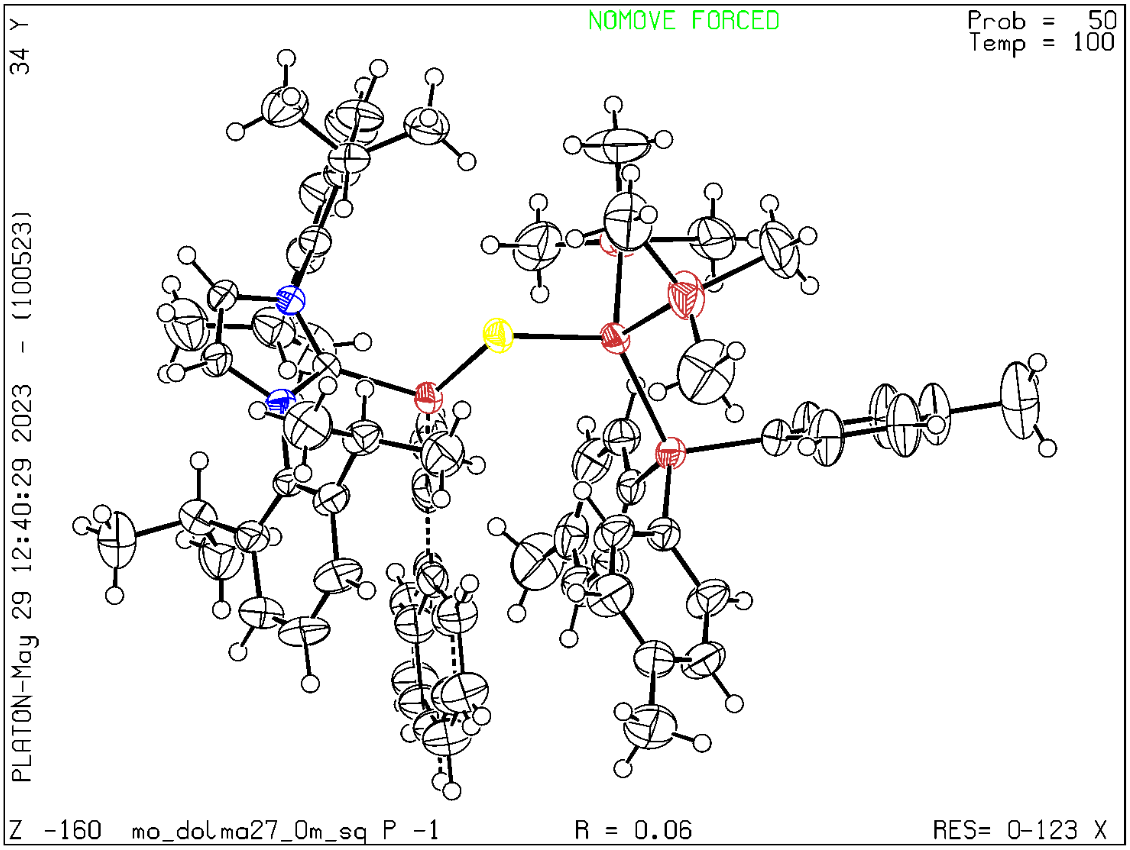

Supplementary Figure 77: Molecular structure of compound **9a** generated in PLATON.

The P1 unit cell contains 4 toluene molecules, that could not be modelled reasonably, hence, they were treated as a diffuse contribution to the overall scattering without specific atom positions by the PLATON_SQUEEZE routine.

Diffractometer operator Jin Yu Liu
scanspeed 15s per frame dx 38mm
2628 frames measured in 12 data sets
phi-scans with delta phi = 0.5
omega-scans with delta omega = 0.5
shutterless mode

Crystal data

$C_{62}H_{81}N_2PSi_5$	$F(000) = 1104$
$M_r = 1025.71$	
Triclinic, *P*	$D_x = 0.914$ Mg m^{-3}

Hall symbol: -P 1

a = 12.3857 (14) Å	Mo $K\alpha$ radiation, λ = 0.71073 Å
b = 12.9608 (13) Å	Cell parameters from 9873 reflections
c = 23.846 (3) Å	θ = 2.2–26.4°
α = 82.886 (4)°	μ = 0.15 mm^{-1}
β = 78.880 (4)°	T = 100 K
γ = 89.947 (4)°	Fragment, orange
V = 3726.1 (7) Å^3	0.47 × 0.28 × 0.27 mm
Z = 2	

Data collection

Bruker Photon CMOS diffractometer	15306 independent reflections
Radiation source: IMS microsource	11219 reflections with $I > 2\sigma(I)$
Helios optic monochromator	R_{int} = 0.087
Detector resolution: 16 pixels mm^{-1}	θ_{max} = 26.4°, θ_{min} = 2.0°
phi– and ω–rotation scans	h = −15 15
Absorption correction: multi-scan *SADABS* 2016/2, Bruker, 2016	k = −16 16
T_{min} = 0.691, T_{max} = 0.745	l = −29 29
118630 measured reflections	

Refinement

Refinement on F^2	Secondary atom site location: difference Fourier map
Least-squares matrix: full	Hydrogen site location: inferred from neighbouring sites
$R[F^2 > 2\sigma(F^2)]$ = 0.056	H-atom parameters constrained
$wR(F^2)$ = 0.133	W = $1/[\Sigma^2(FO^2) + (0.0494P)^2 + 1.7844P]$ WHERE $P = (FO^2 + 2FC^2)/3$
S = 1.02	$(\Delta/\sigma)_{max}$ = 0.001
15306 reflections	$\Delta\rho_{max}$ = 0.36 e Å^{-3}
697 parameters	$\Delta\rho_{min}$ = −0.25 e Å^{-3}
374 restraints	
Primary atom site location: iterative	

5. Computational Details

Calculations were carried out using ORCA 5.0.4 software.[15]

Geometry optimizations were carried using the r^2SCAN-3c composite method, utilizing the regularized and restored SCAN (r^2SCAN) functional,[16,17] geometrical counterpoise correction gCP,[18] the atom-pairwise dispersion correction based on tight binding partial charges (D4),[19-21] the def2-mTZVPP basis set and def2-mTZVPP/J auxiliary basis set.[22]

The optimized geometries were verified as minima or transition states by analytical frequency calculations. The transition states were additionally verified by IRC calculations. Single point calculations of the optimized geometries were carried out at the r^2SCAN-3c level using the SMD solvation module[23] to obtain electrostatic contribution and the cavity term to account for the solvent effects. For more accurate electronic energies, single point calculations of the r^2SCAN-3c optimized geometries were carried using the ωB97M-V[24] functional, the def2-QZVP[25] basis set and def2/J[26] auxiliary basis set. The method is denoted as ωB97M-V(SMD)/def2-QZVP//r^2SCAN-3c. The NBO analysis was done using the NBO7 software,[27] at the PBE0[28] /def2-TZVP[25]/r^2SCAN-3c level of theory.

Supplementary Table 4: Calculated energies (Eh). $E_{ωB97M\text{-}V}$ - electronic energy at the ωB97M-V/def2-QZVPP//r^2SCAN-3c level; G-E_{el} - Gibbs energy minus the electronic energy at the r^2SCAN-3c// r^2SCAN-3c level; G_{cds} (cavity term) and G_{enp} (electrostatic contribution) at r^2SCAN-3c (SMD=Benzene)// r^2SCAN-3c level; G_{conc} - concentration-induced free-energy shift (G_{conc} = RTln(24.5)); G – free energy at the ωB97M-V/def2-QZVPP//r^2SCAN-3c level, G = $E_{ωB97M\text{-}V}$ + [G-E_{el}] + G_{cds} + G_{enp} + G_{conc}. Thermochemistry at 298.15 K.

Compound	ID	$E_{ωB97M\text{-}V}$	G-E_{el}	G_{cds}	G_{enp}	G_{conc}	G
**2**	3513560	-4001.34323	1.0513956	-0.02585692	-0.02767	0.00302	-4000.34234
**2’**	3519997	-4001.34792	1.05124	-0.02633	-0.02620	0.00302	-4000.34618
**5**	3513633	-4001.35374	1.05066102	-0.02649254	-0.02696	0.00302	-4000.35351
**5’**	3519094	-4001.35622	1.05141	-0.02612	-0.02599	0.00302	-4000.35390
**TS(2-5)**	3578344	-4001.30707	1.04963	-0.02242	-0.02725	0.00302	-4000.30409
**10**	3695995	-2841.24374	-	-	-	-	-
**11**	4072606	-2841.22978	-	-	-	-	-
**TS(10-11)**	3695660	-2841.26463	-	-	-	-	-

The summary of the thermochemistry results is presented in Supplementary Table 4. The optimization of the X-ray structure of **2** converges to the geometry denoted as **2**. The properties calculations were carried out on that geometry. The IRC calculation of the **TS(2-5)** transition state and the subsequent optimization to the corresponding minima converge to an isomer of **2**, denoted in the table as **2'**, that this is 2.4 kcal mol^{-1} lower than **2**. Likewise, the geometry of **5** obtained from the X-ray structure slightly differs from that of **5'** obtained for the IRC calculation, and 0.2 kcal mol^{-1} higher in energy.

6. Side-By-Side Comparison of MOs and NBOs in 2/5

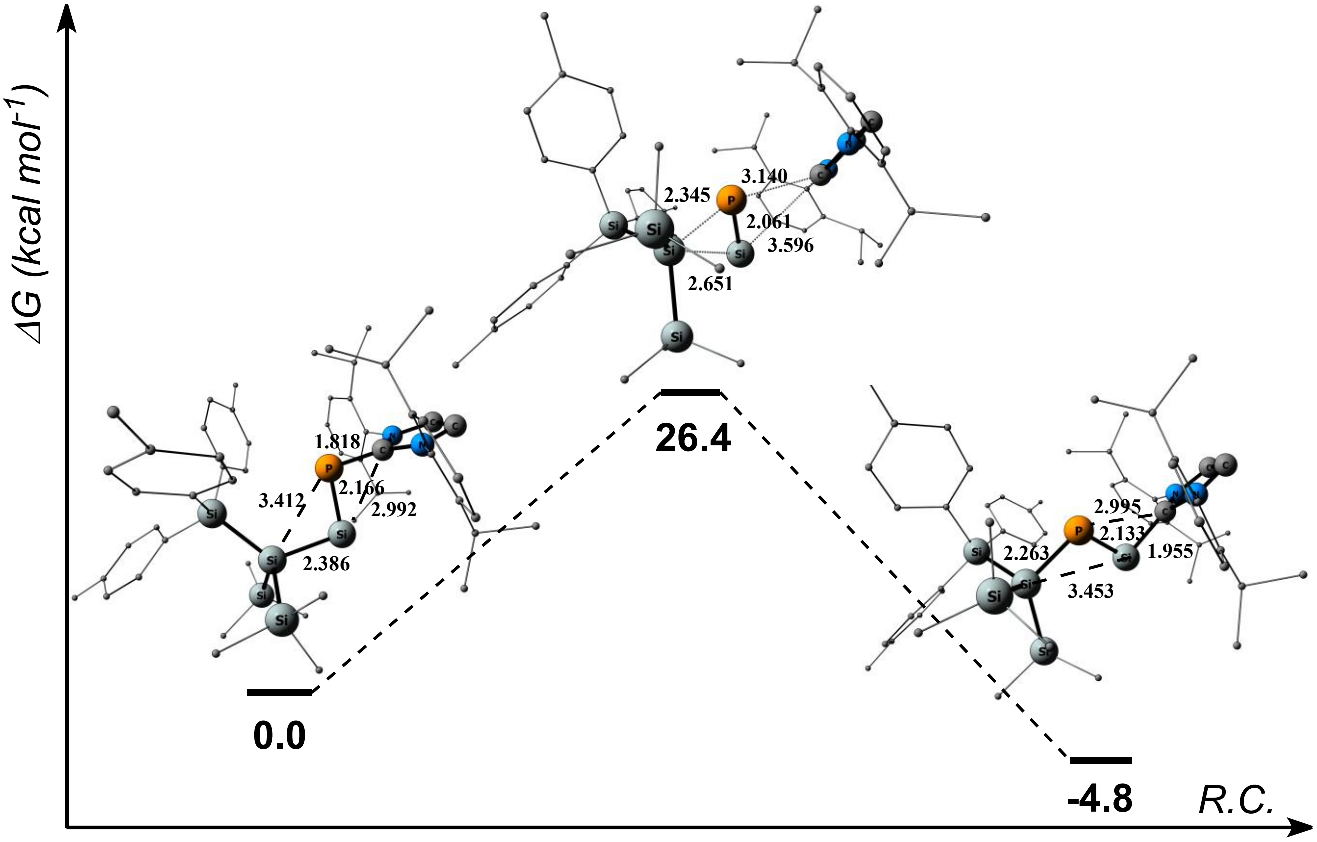

Supplementary Figure 78: Calculated reaction coordinate for the proposed mechanism of **2** → **5** isomerization. Phosphino-silylene **2** (0.0 kcal/mol) isomerizes to phosphasilenylidene **5** (-4.8 kcal/mol) with an energy barrier of 26.4 kcal/mol.

NPA-Charges (2)

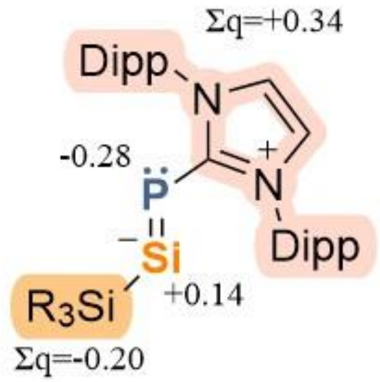

NPA-Charges (5)

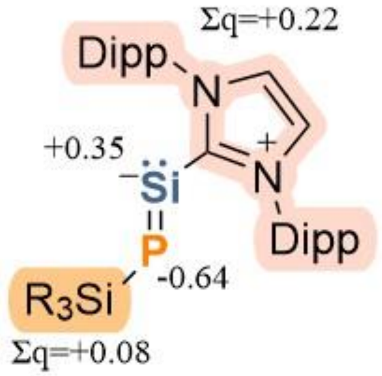

WBI, MBO (2)

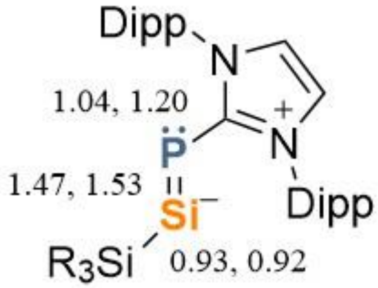

WBI, MBO (5)

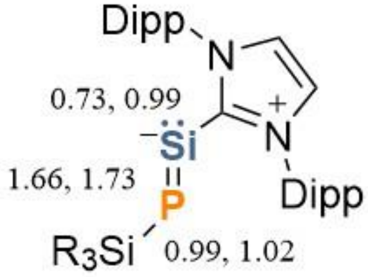

HOMO-1: -5.39 eV (2)

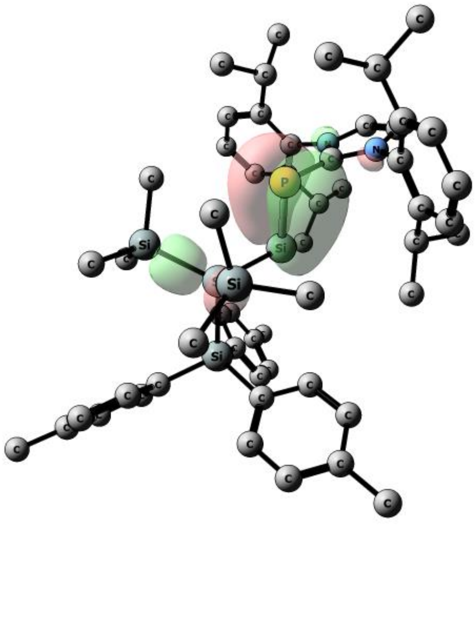

HOMO-1: -5.16 eV (5)

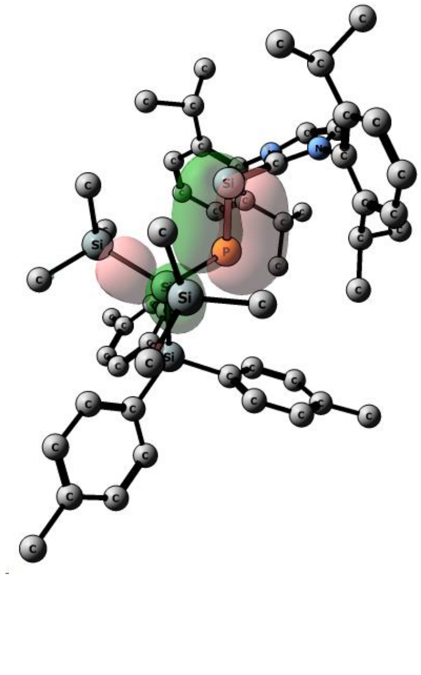

HOMO: -4.74 eV (2)

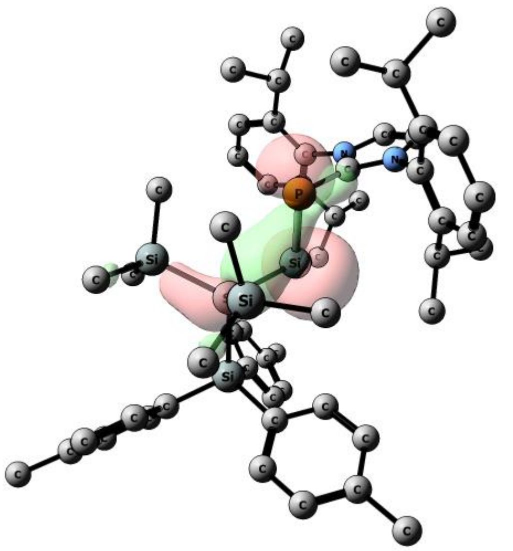

HOMO: -4.89 eV (5)

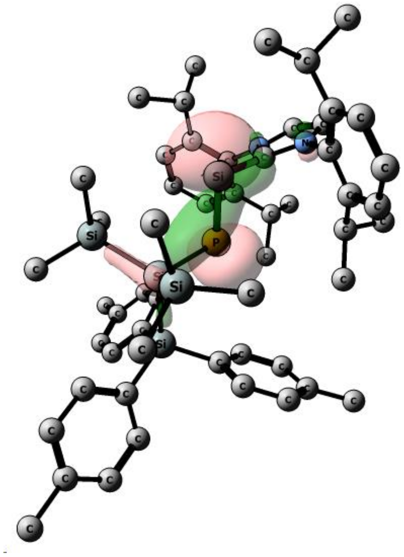

LUMO: -1.36 eV (2)

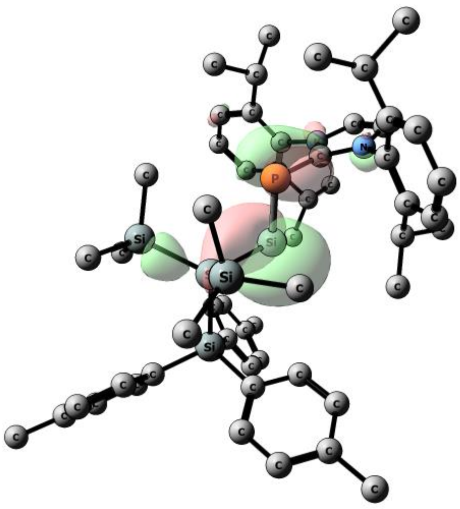

LUMO: -1.35 eV (5)

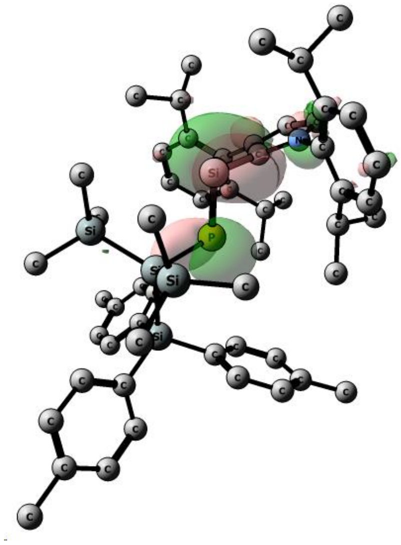

NBOs (2):

87. (1.89) LP P
s(67.62%)p 0.48(32.26%)

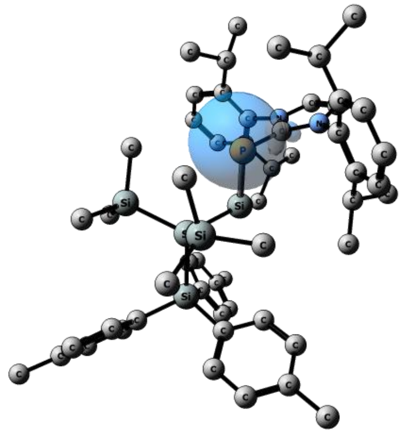

88. (1.95) LP Si
s(75.67%)p 0.32(24.23%)

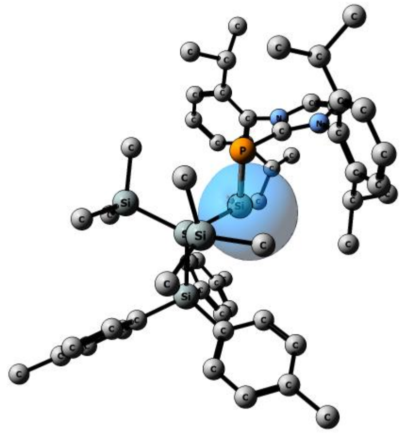

90. (1.91) BD(1) P-Si
70.6% P, 29.4% Si

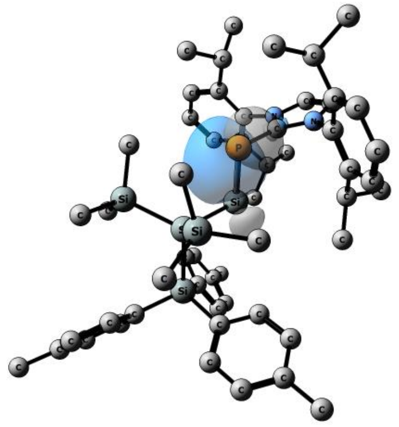

NBOs (5):

87. (1.89) LP Si
s(75.29%)p 0.33(24.62%)

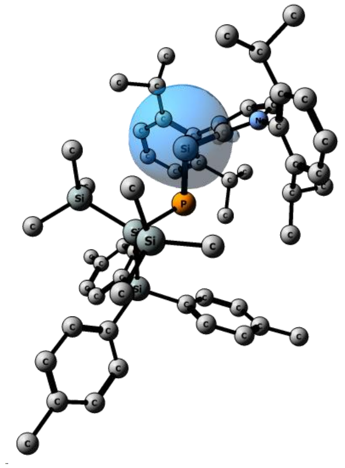

88. (1.95) LP P
s(61.74%)p 0.62(38.11%)

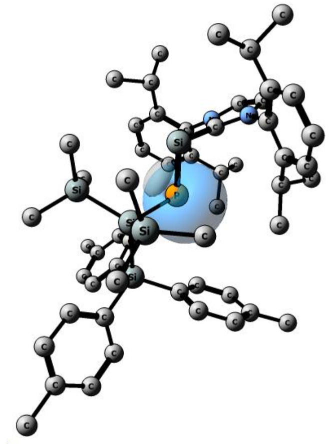

90. (1.93) BD(1) Si-P
34.5% Si, 65.5% P

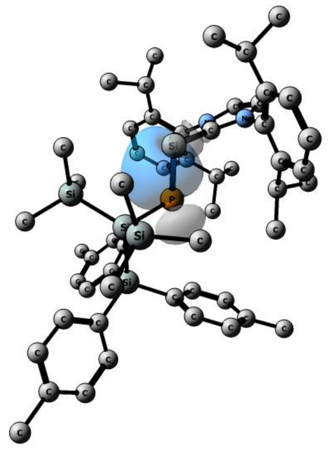

NBOs (2):

91. (1.85) BD(2) P-Si
72.7% P, 27.3% Si

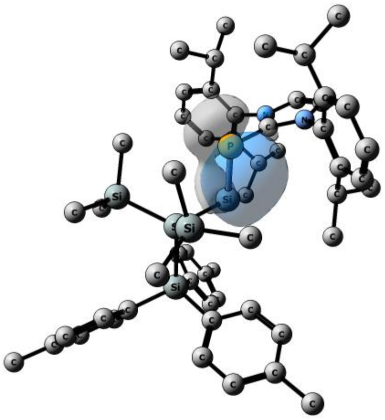

92. (1.96) BD(1) P-C
31.7% P, 68.3% C

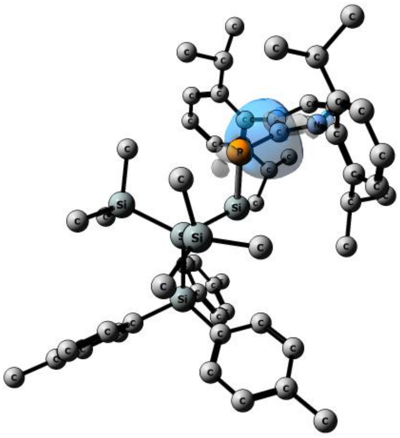

93. (1.92) BD(1) Si-Si’
39.8% Si, 62.2% Si

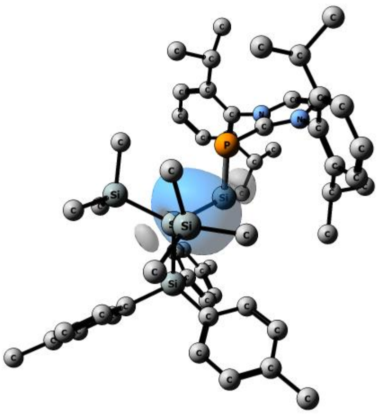

NBOs (5):

91. (1.90) BD(2) Si-P
32.5% Si, 67.5% P

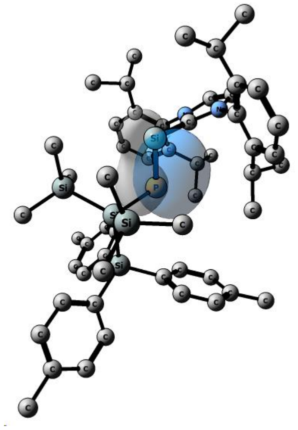

92. (1.95) BD(1) Si-C
20.2% Si, 79.8% C

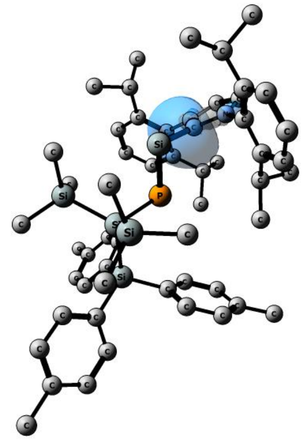

105. (1.95) BD(1) Si’-P
43.3% Si’, 56.7% P

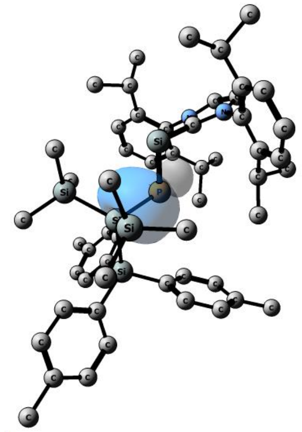

7. Calculations of NHC-Free Derivatives of 2 (10) and 5 (11)

To obtain insight into the electronic structures of compounds **2** and **5** in the absence of the NHC donor stabilization, we optimized the geometries of the corresponding NHC-stabilized phosphasilyne and phosphasilenyliedene (**10** and **11**).

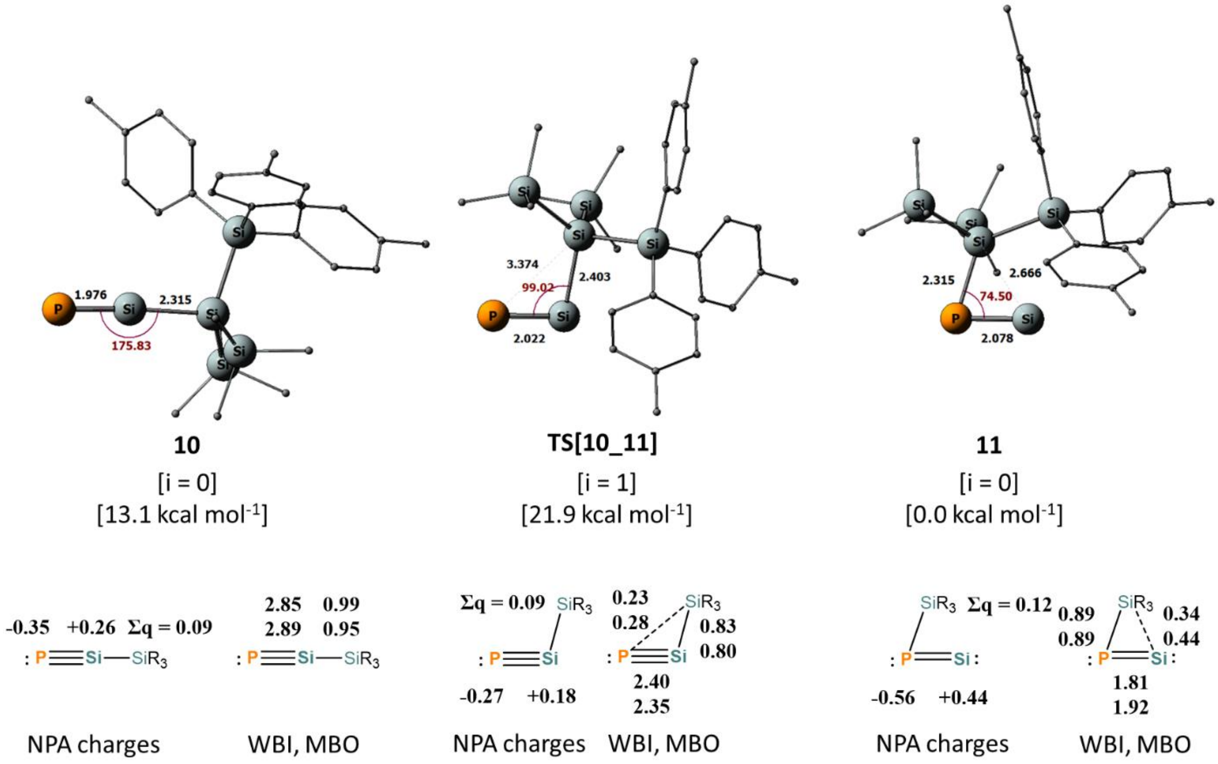

Supplementary Figure 79: Top: Geometries and relative energies of **10** (donor-free **2**), **11** (donor-free **5**) and the transition state of their isomerization **TS[10_11]**. Bottom: NPA-charges, Wiberg bond indices, and Mayer bond orders for the structures depicted above.

Compound **10** is the structural analogue of the linear P-Si-H (Figure 1c in the main text). Thus, **10** is essentially the phosphasilyne **2** without the NHC donor. The r^{2}SCAN-3c optimized structure of **10** shows almost a linear geometry along the P-Si-Si moiety (175.8 °), similar to the linear parent P-Si-H. In the absence of NHC stabilization phosphasilyne **10** exhibits a short P-Si bond length of 1.976 Å, like the previously calculated r(P-Si) = 1.973 in the parent system (Figure 1c in the main text), and an explicit triple bond character of P-Si interaction with WBI and MBO of 2.85 and 2.89 respectively. NBO analysis (@PBE0/def2-TZVP//r^{2}SCAN-3c) shows three bonding interactions P-Si interactions (BD(1) P-Si, 58.14% P, 41.86% Si, occ. 1.98 el.; BD(2) P-Si, 58.50% P, 41.50% Si, occ. 1.98 el.; BD(3) P-Si, 52.96% P, 47.04% Si, occ. 1.98 el.). The corresponding antibonding interactions are of low occupancies (0.04, 0.04. 0.04, respectively), demonstrating a lack of stabilization of the empty antibonding orbitals. This stabilization is achieved in **2** by the external donor (NHC), formally cleaving one of the π bonds and forming the polarized P-C bond (Figure 2a in the main text). The phosphasilyne conformation **10** is calculated to be energetically less favorable than the bent phosphasilenyliedene **11** (ΔE = 13.1 kcal mol-

[1], @ωB97MV/def2-QZVP//r^2SCAN-3c), similarly to the previously calculated parent system (ΔE = 10.3 kcal mol^{-1}).

Compound **11** is the structural analogue of the bent parent H-P-Si (Figure 1c in the main text) and shows the geometry of compound **5** without the NHC donor. In this case, the r^2SCAN-3c optimized structure also shows a bent geometry with α(Si-P-Si) of 74.5°. The Si-P bond length of 2.078 Å is comparable to the previously calculated Si-P bond length of the parent system (2.062 Å). NBO analysis shows the presence of one lone pair on both the low valent P and Si centres. The phosphorus and the silicon form a double bond with WBI = 1.81 and MBO = 1.92. The corresponding NBOs are slightly more polarized toward the Si centre (BD(1) P-Si, 66.80% P, 33.20% Si, occ. 1.96 el.; BD(2) P-Si, 69.29% P, 30.71% Si, occ. 1.91 el.) than those in **10**. This is also reflected in the NPA charges at Si and P in **11** of +0.44 el. and -0.56 el. , respectively, vs. +0.26 el. and -0.35 el. in **10**. Additionally, the low valent Si in **11** has a lone vacancy 3p orbital (LV Si, $sp^{45.29}d^{0.67}f^{0.01}$, occ. 0.36 el.) The stabilization of the low valent silicon centre is accomplished by donor-acceptor interactions between the R_3SiP moiety and the vacant 3p orbital. The predominant donor NBOs are the σ(P-SiR_3), with stabilization energy E(2) of 43.4 kcal mol^{-1}, and σ(Si-$SiPh_3$), with E(2)=31.6 kcal mol^{-1}. These bonding interactions are reflected in relatively high Si-SiR_3 WBI and MBO of 0.34 and 0.44, respectively. These types of interactions are absent in compound **5**. Instead, the interaction between the low valent silicon centre and the carbene is depicted as a polarized Si-C bond (Figure 4 in the main text, NBO 92). Thus, in the absence of an external base, the SiR_3 moiety acts as an intramolecular donor to stabilize the low valent Si centre of the phosphasilenyliedene.

Similarly to the parent system the nearly linear phosphasilyne **10** and the bent phosphasilenyliedene **11** are connected by a transition state **TS[10-11]** at ΔE = 21.9 kcal mol^{-1}. This relative energy is comparable to that previously calculated for the parent system (ΔE = 24.7) kcal mol^{-1} The transition state retains a high Si-P triple bond character (WBI = 2.40, MBO = 2.35). The Si-Si bond is being cleaved with WBI = 0.83, MBO = 0.80, in comparison to **10** (WBI = 0.99, MBO = 0.95), while the P-SiR_3 bond is being formed (WBI = 0.23, MBO = 0.28).

8. References

1 Hintermann, L. Expedient syntheses of the N-heterocyclic carbene precursor imidazolium salts IPr·HCl, IMes·HCl and IXy·HCl. *Beilstein J. Org. Chem.* **3**, 22, doi:10.1186/1860-5397-3-22 (2007).

2 Tondreau, A. M., Benkő, Z., Harmer, J. R. & Grützmacher, H. Sodium phosphaethynolate, Na(OCP), as a "P" transfer reagent for the synthesis of N-heterocyclic carbene supported P3 and PAsP radicals. *Chem. Sci.* **5**, 1545-1554, doi:10.1039/C3SC53140F (2014).

3 Schmidt, P., Fietze, S., Schrenk, C. & Schnepf, A. Bulky Phenyl Modifications of the Silanide Ligand Si(SiMe3)(3) - Synthesis and Reactivity. *Z. Anorg. Allg. Chem.* **643**, 1759-1765, doi:10.1002/zaac.201700253 (2017).

4 Muhr, M. *et al.* Enabling LIFDI-MS measurements of highly air sensitive organometallic compounds: a combined MS/glovebox technique. *Dalton Trans.* **50**, 9031-9036, doi:10.1039/D1DT00978H (2021).

5 *Bruker AXS Inc. APEX suite of crystallographic software; Madison, Wisconsin, USA*. (2021).

6 *SAINT, Version 8.40A and SADABS, Version 2016/2, Bruker AXS Inc., Madison, Wisconsin, USA*. (2016/2019).

7 Hubschle, C. B., Sheldrick, G. M. & Dittrich, B. ShelXle: a Qt graphical user interface for SHELXL. *Journal of Applied Crystallography* **44**, 1281-1284, doi:doi:10.1107/S0021889811043202 (2011).

8 Sheldrick, G. SHELXT - Integrated space-group and crystal-structure determination. *Acta Crystallographica Section A* **71**, 3-8, doi:doi:10.1107/S2053273314026370 (2015).

9 Sheldrick, G. Crystal structure refinement with SHELXL. *Acta Crystallographica Section C* **71**, 3-8, doi:doi:10.1107/S2053229614024218 (2015).

10 *International Tables for Crystallography, Vol. C (Ed.: A. J. Wilson), Kluwer Academic Publishers, Dordrecht, The Netherlands, Tables 6.1.1.4 (pp. 500–502), 4.2.6.8 (pp. 219–222), and 4.2.4.2 (pp. 193–199).* (1992).

11 Kratzert, D. & Krossing, I. Recent improvements in DSR. *Journal of Applied Crystallography* **51**, 928-934, doi:doi:10.1107/S1600576718004508 (2018).

12 Macrae, C. F. *et al.* Mercury CSD 2.0 - new features for the visualization and investigation of crystal structures. *Journal of Applied Crystallography* **41**, 466-470, doi:doi:10.1107/S0021889807067908 (2008).

13 Spek, A. PLATON SQUEEZE: a tool for the calculation of the disordered solvent contribution to the calculated structure factors. *Acta Crystallographica Section C* **71**, 9-18, doi:doi:10.1107/S2053229614024929 (2015).

14 Spek, A. Structure validation in chemical crystallography. *Acta Crystallographica Section D* **65**, 148-155, doi:doi:10.1107/S090744490804362X (2009).

15 Neese, F. Software update: The ORCA program system—Version 5.0. *WIREs Computational Molecular Science* **12**, e1606, doi:https://doi.org/10.1002/wcms.1606 (2022).

16 Furness, J. W., Kaplan, A. D., Ning, J., Perdew, J. P. & Sun, J. Correction to "Accurate and Numerically Efficient r2SCAN Meta-Generalized Gradient Approximation". *The Journal of Physical Chemistry Letters* **11**, 9248-9248, doi:10.1021/acs.jpclett.0c03077 (2020).

17 Furness, J. W., Kaplan, A. D., Ning, J., Perdew, J. P. & Sun, J. Accurate and Numerically Efficient r2SCAN Meta-Generalized Gradient Approximation. *The Journal of Physical Chemistry Letters* **11**, 8208-8215, doi:10.1021/acs.jpclett.0c02405 (2020).

18 Kruse, H. & Grimme, S. A geometrical correction for the inter- and intra-molecular basis set superposition error in Hartree-Fock and density functional theory calculations for large systems. *J. Chem. Phys.* **136**, doi:10.1063/1.3700154 (2012).

19 Caldeweyher, E., Bannwarth, C. & Grimme, S. Extension of the D3 dispersion coefficient model. *J. Chem. Phys.* **147**, doi:10.1063/1.4993215 (2017).

20 Caldeweyher, E. *et al.* A generally applicable atomic-charge dependent London dispersion correction. *J. Chem. Phys.* **150**, doi:10.1063/1.5090222 (2019).

21 Caldeweyher, E., Mewes, J.-M., Ehlert, S. & Grimme, S. Extension and evaluation of the D4 London-dispersion model for periodic systems. *Physical Chemistry Chemical Physics* **22**, 8499-8512, doi:10.1039/D0CP00502A (2020).

22 Grimme, S., Hansen, A., Ehlert, S. & Mewes, J.-M. r2SCAN-3c: A "Swiss army knife" composite electronic-structure method. *J. Chem. Phys.* **154**, doi:10.1063/5.0040021 (2021).

23 Marenich, A. V., Cramer, C. J. & Truhlar, D. G. Universal Solvation Model Based on Solute Electron Density and on a Continuum Model of the Solvent Defined by the Bulk Dielectric Constant and Atomic Surface Tensions. *The Journal of Physical Chemistry B* **113**, 6378-6396, doi:10.1021/jp810292n (2009).

24 Mardirossian, N. & Head-Gordon, M. ωB97M-V: A combinatorially optimized, range-separated hybrid, meta-GGA density functional with VV10 nonlocal correlation. *J. Chem. Phys.* **144**, doi:10.1063/1.4952647 (2016).

25 Weigend, F. & Ahlrichs, R. Balanced basis sets of split valence, triple zeta valence and quadruple zeta valence quality for H to Rn: Design and assessment of accuracy. *Physical Chemistry Chemical Physics* **7**, 3297-3305, doi:10.1039/B508541A (2005).

26 Weigend, F. Accurate Coulomb-fitting basis sets for H to Rn. *Physical Chemistry Chemical Physics* **8**, 1057-1065, doi:10.1039/B515623H (2006).

27 *NBO 7.0. E. D. Glendening, J, K. Badenhoop, A. E. Reed, J. E. Carpenter, J. A. Bohmann, C. M. Morales, P. Karafiloglou, C. R. Landis, and F. Weinhold, Theoretical Chemistry Institute, University of Wisconsin, Madison* (2018).

28 Adamo, C. & Barone, V. Toward reliable density functional methods without adjustable parameters: The PBE0 model. *J. Chem. Phys.* **110**, 6158-6170, doi:10.1063/1.478522 (1999).
